# The sensory representation of causally controlled objects

**DOI:** 10.1016/j.neuron.2020.12.001

**Published:** 2021-02-17

**Authors:** Kelly B. Clancy, Thomas D. Mrsic-Flogel

**Affiliations:** 1 Biozentrum, University of Basel, 70 Klingelbergstrasse, 4056 Basel, Switzerland

**Keywords:** brain machine interface, brain computer interface, widefield imaging, calcium imaging, sensorimotor learning, parietal cortex, decoder, causal control, visual cortex, neuroprosthetics

## Abstract

Intentional control over external objects is informed by our sensory experience of them. To study how causal relationships are learned and effected, we devised a brain machine interface (BMI) task using wide-field calcium signals. Mice learned to entrain activity patterns in arbitrary pairs of cortical regions to guide a visual cursor to a target location for reward. Brain areas that were normally correlated could be rapidly reconfigured to exert control over the cursor in a sensory-feedback-dependent manner. Higher visual cortex was more engaged when expert but not naive animals controlled the cursor. Individual neurons in higher visual cortex responded more strongly to the cursor when mice controlled it than when they passively viewed it, with the greatest response boosting as the cursor approached the target location. Thus, representations of causally controlled objects are sensitive to intention and proximity to the subject’s goal, potentially strengthening sensory feedback to allow more fluent control.

## Highlights

• Mice controlled a visual cursor using population neural activity in cortical areas
• Animals could decorrelate normally correlated cortical areas to perform the task
• Introduction of a new rule triggered stochastic exploration of activity patterns
• Controlled objects elicited more robust sensory responses in a goal-oriented manner

## Teaser

Clancy and Mrsic-Flogel investigate how the perception of a causally controlled object is different than one passively viewed. Mice learn to control a visual cursor using brain activity alone. Neural responses to the controlled cursor include more information about the present and future cursor positions than during passive cursor viewing.

## Introduction

How does the brain infer a causal relationship between its activity and the sensed world, and how does this affect the sensory encoding of controlled external objects? Actions and perceptions reciprocally affect one another in a mutual dialog (Dewey, 1896). The sense of control can be operationalized to inferring a causal relationship between a subject’s internally generated actions or activity and their outcome in the external world (Haggard, 2017). In motor learning, for example, the relationship between an action and its outcome can be learned and re-learned throughout adulthood as animals acquire new motor skills. Brain machine interfaces (BMIs) are a method for investigating how subjects learn arbitrary action-outcome relationships (Fetz, 1969; Bakay and Kennedy, 1998; Nicolelis, 2001; Donoghue, 2002; Carmena et al., 2003; Sitaram et al., 2017). When learning to control a BMI, rodents have been found to use the same mechanisms as implicated in motor learning (Koralek et al., 2012; Neely et al., 2018). However, unlike motor learning, wherein animals learn a task and researchers must search for correlates of the behavior in patterns of neural activity, BMIs allow the experimenter to precisely control sensory feedback and prescribe the requisite activity patterns necessary for successful task execution, which can then be changed day to day. Thus, animals learning neuroprosthetic control of external objects must engage in continuous self-monitoring to assess the contingency between their neural activity and its outcome, preventing them from executing a habitual or fixed motor pattern, and encouraging animals to learn arbitrary new sensorimotor mappings on the fly.

A key aspect of this self-monitoring is the sensory feedback from the object being controlled by the agent. However, little is known about how causally controlled objects are represented in the brain. Studies have implicated the parietal cortex in intention and in the subjective assessment of agency over outcome. In human subjects, disrupting activity in the parietal cortex temporarily ablates self-reported agency (Chambon et al., 2015). Parietal activity has been found to be involved in representing task rules, the value of competing actions, and visually guided real-time motor plan updating, both in humans (Pisella et al., 2000; Kahnt et al., 2014; Wisniewski et al., 2015; Zapparoli et al., 2020) and non-human primates (Andersen et al., 1997; Sugrue et al., 2004). Motor plans can be decoded from parietal activity, and its responses are task, expectation, and goal dependent, in humans, (Rushworth et al., 2001; Desmurget et al., 2009; Aflalo et al., 2015), non-human primates (Mountcastle et al., 1975; Gnadt and Andersen, 1988; Lacquaniti et al., 1995; Johnson et al., 1996; Churchland et al., 2008), and rodents (Licata et al., 2017; Pho et al., 2018; Mohan et al., 2018, 2019). All of this evidence suggests that, across multiple species, the parietal cortex plays a role in intentional, goal-directed behaviors (Rizzolatti et al., 1997; Andersen and Buneo, 2002; Andersen and Cui, 2009). However, previous studies of the role of the parietal cortex in intention have not examined how causal control affects sensory representations across different sensorimotor contingencies.

To address this, we devised a mouse model of adaptive causal control. Control is traditionally studied through the lens of motor actions, which makes comparing sensory responses across control and passive conditions difficult, given that the former involves movement-related signals. By using a BMI task, we minimized movement-related differences in the neural responses between the conditions. Animals learned to guide a visual feedback cursor to a target location to obtain a reward using neural activity in experimenter-defined cortical areas, recorded with wide-field imaging. This had the added benefit of acting as an unbiased screen to identify dorsal cortical areas involved in learning the task. We found that higher visual areas, including the anteromedial cortex (AM), were more engaged when expert animals controlled the BMI. These higher areas are considered by some to be a putative homolog of parietal cortex in mice (Harvey et al., 2012; Licata et al., 2017; Mohan et al., 2018; Pho et al., 2018; Lyamzin and Benucci, 2019; Gilissen et al., 2020). To gain insight into what this task-related activity looks like on an individual neuron level, we targeted single-cell recordings to the functionally identified task-relevant region AM, and found that the visual cursor elicited larger responses when an animal was controlling it in a closed-loop configuration than when passively viewing it in an open-loop configuration (Bagur et al., 2018). Responses were highest when the cursor was closest to the target zone and were sensitive to the cursor’s instantaneous trajectory: they were greater when the cursor was moving toward the target than away from it. Thus, the sensory representation of the visual object was sensitive to the subject’s intention and its perception of the object’s instantaneous trajectory with respect to its goal. Given that animals had to relearn a changing sensorimotor contingency on the fly, we surmised that the heightened sensory representation of the cursor may serve to strengthen the signal to adjudicating areas for informing fluent control over external objects. The neural activity in AM during the task condition carried significantly more information about the cursor identity than during the passive playback condition.

## Results

### Goal-directed control of a visual cursor using areal signals

**Figure 1:**
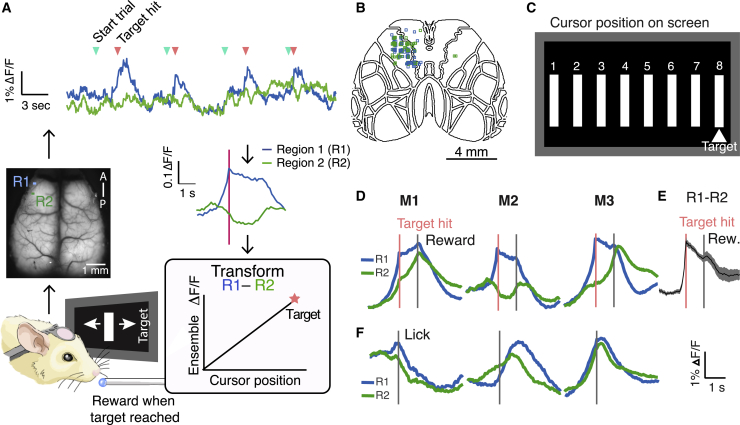
A widefield-imaging-based brain machine interface (A) Task schematic. Clockwise starting from illustration of mouse: wide-field signals were imaged from head-fixed animals in real time and transmuted into the position of a visual cursor. Two small regions (R1 and R2) were used for controlling the cursor, and activity recorded from these areas was fed into a decoder such that their activity opposed one another. Example dF/F for the 2 regions is shown at top, with blue arrows denoting trial starts, and pink arrows denoting target hits. Activity averaged around hits for 1 example animal, 1 day, shown for R1 and R2. Animals had to increase activity in R1 relative to R2 to bring the cursor to a rewarded position at the center of their visual field, at which point they could collect a reward after a 1-s delay. (B) Positions of control ROIs (R1 in blue, R2 in green) for all 7 animals over the course of training (averaging 15 days each), superimposed on the Allen Brain Atlas (totaling 104 pairs). (C) Feedback schematic: the cursor could take 1 of 8 potential positions on screen, with position 8, the target, rewarded. (D) ΔF/F in control regions triggered around hits for 3 example animals on 1 day of training, indicating different strategies that animals use to achieve reward. Pink line indicates the time of target hit and gray line indicates reward delivery. (E) Activity in R1 subtracted by activity in R2, averaged around target hits for all mice on a day of training (n = 7 mice, shading represents SEM). (F) Animals could not control cursor using lick alone. ΔF/F triggered around lick bouts in spontaneous activity for the same 3 example animals on 1 day of training, indicating animals could not achieve the differential activation of R1 and R2 using lick alone. Gray line indicates time of lick.

To investigate how causal control over external objects is effected and encoded in mammalian cortex, we trained mice to control a visual feedback cursor using real-time calcium signals recorded with wide-field imaging (largely reflecting the summed spiking of local cells; Makino et al., 2017; Clancy et al., 2019). We imaged the dorsal cortex in transgenic mice expressing the calcium indicator GCamp6s in CaMKII^+^ pyramidal neurons (Wekselblatt et al., 2016), assigning two small frontal regions to control the cursor (Figures 1A and 1B; Videos S1, S2, and S3), similar to a task described previously (Clancy et al., 2014; Koralek et al., 2012). Animals were head-fixed under the wide-field microscope, and free to run on a styrofoam wheel. The animal’s goal was to bring the cursor (a copy of which was presented to both eyes on two separate monitors flanking each side of the mouse) to a target position in the center of its visual field (Figure 1C). Animals could achieve this by increasing activity in control region 1 (R1) relative to control region 2 (R2). If activity in R2 was greater than R1, then the cursor moved toward the back of the animal’s visual field. Thus, in this design, animals could not hit the target simply by generally increasing or decreasing activity across the cortex, but had to differentially modulate the activity of these specific regions (Figures 1D–1F, S1A, and S1B). The control regions were usually placed over ipsilateral motor areas and were changed from day to day (Figure 1B; Table S1). Control regions were deliberately small (∼0.1 mm^2^) as we reasoned that it would be easier for animals to control a smaller area, but this was not systematically tested. We avoided using the anterior lateral motor cortex (ALM) in the control regions, to minimize the effect of licking.

Video S1. Activity averaged around hits, related to Figure 1 Video of cortical activity averaged around hits. Time from hit (in seconds) is indicated at the top left, and control regions are indicated (region 1 with a blue box, and region 2 with a green box). Animal performs task by sweeping activity laterally from region 1 toward region 2 at the time of the hit. Lick and forepaw activity are then evident as animal consumes reward.

Video S2. Activity averaged around hits, related to Figure 1 Video of cortical activity averaged around hits. Time from hit (in seconds) is indicated at the top, and control regions are indicated (region 1 with a blue box, and region 2 with a green box). Activity in region 1 increases compared to region 2 at the time of the hit. Lick and forepaw activity are then evident as animal consumes reward.

**Figure 2:**
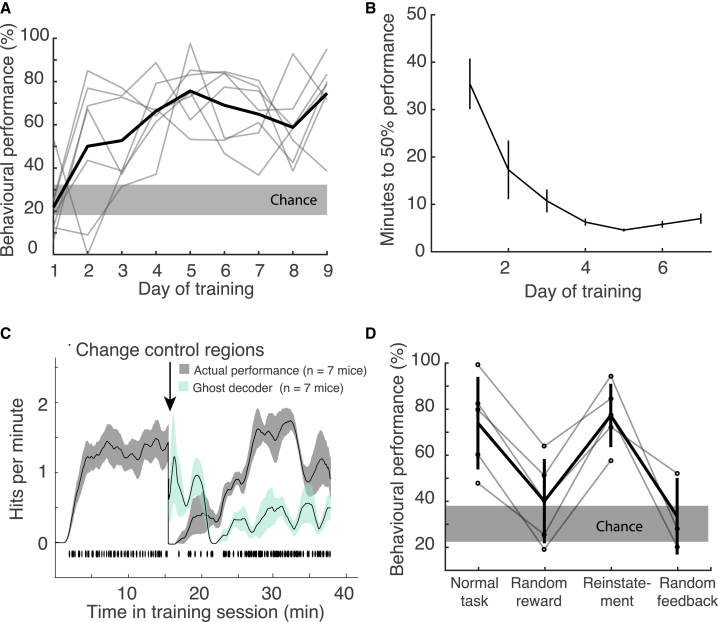
Animals learn to control a visual cursor using areal neural activity (A) Behavioral performance (percentage of trials the animal successfully reached the target within the 30-s trial window) increased above chance over the course of days. Shaded region denotes chance SEM, assessed as how often spontaneous activity would achieve hits, averaged across 7 mice. (B) Animals achieve 50% performance faster over the course of days of training, calculated by taking a moving average of the number of times the animal successfully reached the target per trial. Error bars denote SEM averaged across 7 mice. (C) Average hits per minute (gray) increased over the course of a training session and recovered within minutes when control regions were changed (n = 7 mice on day 6 of training; shading denotes 95% confidence interval, see Table S1). Individual hit times, pooled for all animals, shown below rate curves. At chance baseline, animals should perform ~0.3 hits/min (here, animals started at slightly worse than chance, likely due to the fact that some of the control region positions had been changed from the previous day). After R1 and R2 were swapped mid-session, the estimate of how well the animals would have done using the original control regions is shown in teal: the performance of the previous (“ghost”) decoder starts near the hit rate prior to the switch, suggesting that mice persist with their original strategy, but this drops off as animals discover the new rewarded contingency and recover their performance. (D) Performance dropped to near chance when rewards were given randomly but recovered when target and reward was again coupled on a subsequent day (n = 5 mice). Performance also dropped to chance when the visual feedback cursor was uncoupled from neural activity and presented at random (n = 3 mice), even though animals could still achieve reward using the previously learned neural activity pattern (i.e., reward was no longer linked to cursor position). Error bars denote standard deviation. Shaded region denotes chance SEM, assessed as how often spontaneous activity would achieve hits (n = 7 mice).

Video S3. Discovery of successful activity patterns on a day of training where new control regions were introduced, related to Figures 1 and 2 (Top) Animals had to sufficiently increase activity in control region 1 (R1) relative to region 2 (R2) to bring the visual cursor to position 8 for 300 ms to receive reward. Video of continuous recordings from R1 (blue trace) and R2 (green trace) on a day of training when new control regions were introduced. If the animal failed to do this within 30 s, it was considered a failure trial (only one of every 3 frames is shown). Cyan vertical bars indicate trial starts; magenta vertical bars indicate a successful target hit; black vertical bars indicate the end of a failure trial. Top traces in black indicate running velocity (top trace) and lick bouts (second trace). The R1-R2 trace is indicated in the bottommost black trace. (Middle) Reconstruction of the visual feedback cursor position at the time in the recording indicated by the vertical red bar in the top panel. The visual cursor disappeared from the screens in between trials. (Bottom) Activity in dorsal cortex as animal performs the task. The location of control region 1 is denoted with a blue box, control region 2 with a green box.

The visual feedback cursor could take one of eight positions on the monitors (Figure 1C), and the cursor had to be held at the target for 300 ms to count as a hit. When animals succeeded in holding the cursor at the target position, a soya milk reward was delivered after a 1-s delay. If animals failed to bring the cursor to the target position within 30 s, then the trial was a miss, and a white noise miss-cue was followed by a time out. Chance performance was assessed using spontaneous activity recorded before the task began and represented the estimated hit rate that the animal would have achieved using spontaneous fluctuations of neural signals alone. Mice improved their performance over training (Figure 2A; n = 7 mice) and took less time to reach a criterion performance of 50% hit trials over days (Figure 2B).

Animals could perform the task without overt movements, licking, or eye saccades (Figures S1C–S1E). Several recent papers have shown that many motor activities can influence cortical activity (Stringer et al., 2019; Musall et al., 2019; Orsolic et al., 2019; Salkoff et al., 2020), raising the possibility that mice may adopt a motor strategy to control the activation of cortical regions used for BMI. Our analyses show that animals could perform the task without gross overt movements that were detected by monitoring licking, eye saccades, or running speed (Figures S1C–S1E), although it is possible that subtler movement were not detected. This is a question common to all BMI studies that is ultimately unanswerable without recordings from every muscle in the body. We did not take body videos of the animals performing the task, but future work that included video monitoring would offer insights on subtler motor outputs. Nevertheless, animals in this study could discover arbitrary activation patterns of different brain areas within and between training sessions and use these activations to control a visual cursor in a manner dependent on sensory feedback: that is, they could flexibly work out the arbitrary coupling of action patterns (whether purely confined to the brain, or involving peripheral muscle contractions) with sensory feedback to achieve a goal. The flexibility with which the animals could readjust their control of arbitrary brain areas suggests some degree of adaptability in reducing correlations of otherwise highly correlated brain areas (see below), although this varied for different regions.

Control regions could be changed from day to day or within a session: hit rates improved over time within a session and recovered after control regions were changed, indicative of learning (Figures 2C, S2A, and S2B). After recovery, the animals’ hit rates before and after the switch were unchanged (Figure S2C). The decoder transformation for both the pre- and post-switch conditions was calculated using the same spontaneous baseline taken before the training session. To test whether the behavior was goal directed, we dissociated the reward from the target position. On day 8 of training, animals were allowed to perform the task as usual, but the training session was constrained to 30 min. Thereafter, the visual feedback was coupled to the animal’s neural activity as before, but rewards were given randomly, at the same rate as an expert animal engaged in the task (∼1.3 hits/min). Given random rewards, the animals’ target hit rate dropped to chance, indicating that target hits were goal oriented (Figure 2D). The fluorescence signals representing the difference between R1 and R2 increased over the normal training session, indicative of the increased efficacy of control and decreased when the reward was randomized, again suggesting that animals were effecting the requisite neural patterns in a goal-directed manner (Figures S2D and S2E). Animals were able to recover their performance following reinstatement of the normal task on the next day of training (Figure 2D). For a subset of animals, the visual feedback was then randomized: the visual cursor was presented at random positions, although animals could still achieve the target with the appropriate neural activity patterns. The animals’ ability to bring the cursor to the target dropped to chance levels without meaningful visual feedback (Figure 2D). Activity leading to hits did not resemble activity induced by licking around reward collection, as evidenced in both the normal task and random-reward conditions (Figure S2F). This suggests that animals performed the task in a flexible, goal-directed, visual feedback-dependent manner.

### Exploration and exploitation in neural activity space

**Figure 3:**
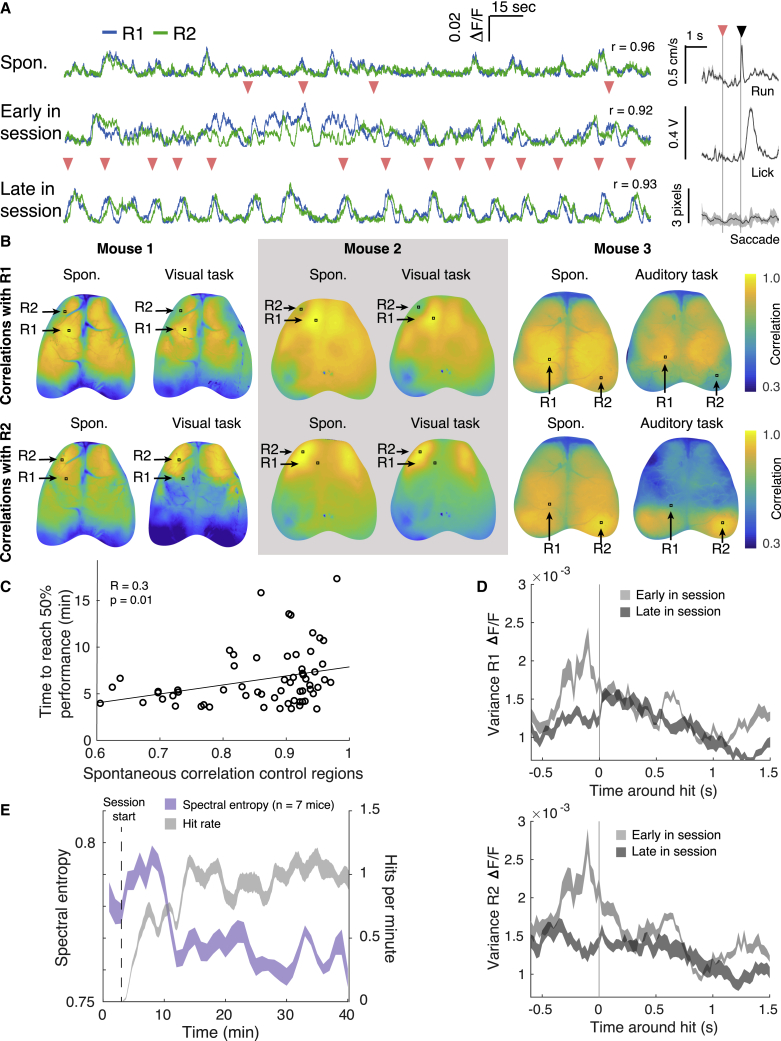
Exploration and exploitation of neural activity patterns (A) Areal signals were highly spontaneously correlated before the training session (top trace). Animals explored different activation patterns early in the training session (center trace) to discover and exploit successful patterns by the end of the session (bottom trace). Pink arrows denote target hits. Pearson’s correlation between R1 and R2 indicated on the right of each trace. Right: run, lick, and saccade averaged around hits for this training session; gray shading denotes 95% confidence interval around mean. Pink arrow denotes target hits, black arrow denotes reward delivery. (B) Correlation map across cortex for 3 animals using activity in R1 (top row) and R2 (bottom row) as seeds, during spontaneous activity and during the BMI task. Mouse 3 was part of a separate cohort trained on an auditory task (see Method Details). Animals could decorrelate normally correlated areas for task performance. (C) Animals took longer to reach criterion performance (50% hits/attempt) if control regions were highly spontaneously correlated (linear regression on data from 7 mice, 9 days of training starting on day 4). (D) The average variance of activity for R1 (top panel) and R2 (bottom panel) was greater around hits early in a training session than late in the session (n = 7 mice, day 8 of training; shading indicates 95% confidence interval around mean), indicating that mice honed in on more reliable and reproducible control strategies over the course of a single training session. (E) Early in the session, neural activity around the control regions had high spectral entropy (a proxy of signal randomness) as animals used stochastic bursts of activity to explore the neural patterns that would yield reward. By late in the session, animals had discovered a successful activity pattern to exploit, and spectral entropy in control area activity decreased. Shaded area indicates 95% confidence interval around mean.

As control regions were changed day to day, the activity patterns necessary for successful BMI control had to be re-learned each session. Example fluorescence traces from control regions indicate that the areas were initially highly spontaneously correlated (Figures 3A [top trace], S1A, and S1B; Video S3). Early in the training session, hits were preceded by diverse activity patterns (Figure 3A, center trace). By late in the training session, activity patterns leading to hits were more consistent (Figure 3A, bottom trace). Animals found different ways to achieve this consistency, sometimes by sweeping activity through R1 toward R2, or by depressing R2 while activating R1 (Figure 1D; Videos S1, S2, and S3).

To achieve the prescribed activity pattern, animals had to functionally decorrelate the two control regions, which were usually spontaneously correlated. Activity in the dorsal cortex was globally correlated pre-task, as indicated by correlation maps using R1 and R2 as seed pixels (Figures 3B and S1B). Correlations between these areas decreased during task performance (Figures 3B and S3B), as did the correlations between the control regions and the primary visual cortex, V1 (Figure S3C). Correlations between the control regions and the primary somatosensory cortex, S1, as well as between S1 and V1, were unchanged between spontaneous activity and the task (Figures S3C and S3D). Only periods of task performance, and not reward collection or inter-trial waiting periods, were included in these analyses. Animals could arbitrarily reduce correlations between different regions sufficient to perform the task. In a separate cohort of animals trained using auditory feedback instead of visual feedback, animals could also decorrelate visual control areas (Figures 3B, rightmost panel, and S4). Interestingly, the task-induced correlation patterns were invariably bilateral, even when control regions were ipsilateral to each other. Not every training session resulted in the kind of decorrelated maps evident in Figure 3B; reflecting that some areas are harder for the animals to decorrelate than others. Animals took longer to reach criterion 50% performance when control regions were spontaneously highly correlated (Figure 3C). The distance between control regions did not affect the time animals required to reach 50% performance (Figure S3A), suggesting that areas that were the most spontaneously correlated, irrespective of their distance from one another, were the hardest to use to perform the task.

The variance in R1 and R2 activity peaked around hits early in a training session as animals explored strategies that would yield reward, as has been shown previously for the activity of individual cells learning to control a BMI device using a fixed decoder (Zacksenhouse et al., 2007; Athalye et al., 2017). This variance decreased later in the session as animals discovered and exploited reliable, reproducible strategies (Figure 3D). Early in the session, the spectral entropy of activity (a measure of the spectral power distribution of a signal, and a proxy for its complexity; see Method details) around the control regions increased as animals explored activity patterns that would yield reward, then dramatically dropped as they discovered successful patterns and reliably exploited them (Figure 3E). At the start of a session, or when control regions were changed, animals faced with uncertain task rules “explored” activity space stochastically, and gradually switched to effecting stereotyped patterns they could reliably exploit after having probed the rules of their environment (Tervo et al., 2014).

### Expert performance correlated with increased activity in higher visual areas

**Figure 4:**
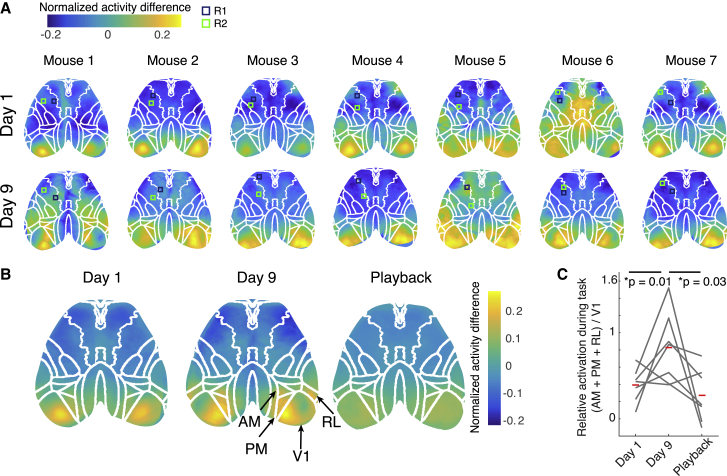
Higher visual areas were more active during expert task performance (A) Activation maps for individual animals on day 1 of training (top row), when animals were naive, versus day 9 of training (bottom row), when animals performed the task expertly, calculated using the normalized activity difference for task-on minus task-off periods. Each map has been registered to the Allen Brain Atlas (overlaid) using stereotaxic marks. Control regions are shown as slightly larger than they actually were for better visibility. (B) Activation maps during task performance on day 1, day 9, and during passive playback of a previous session (representing the normalized activity difference for task-on minus task-off periods). Each map has been registered to the Allen Brain Atlas by stereotaxic marks and then averaged across 7 mice. (C) The relative ratio of task activation in higher visual areas versus V1 increased over training. When animals passively viewed playback of the same session, higher visual areas were less active. Red bars indicate mean ratios (n = 7 mice, paired t test, Bonferroni corrected).

We sought to determine what cortical areas were most active during task performance, and how this changed over learning. On the first day of training, the primary visual cortex was most active during the task, but as animals became expert in the task, higher visual areas were recruited (Figures 4A–4C): in particular, AM, posteromedial cortex (PM), and rostrolateral visual cortex (RL), similar to previous studies of learning visually guided tasks in mice (Wekselblatt et al., 2016; Orsolic et al., 2019). Areas AM and RL are considered by some to be parietal cortex homologs in the mouse cortex, although there is no widespread agreement on this point (Harvey et al., 2012; Licata et al., 2017; Mohan et al., 2018; Pho et al., 2018; Lyamzin and Benucci, 2019; Gilissen et al., 2020). After the final day of training, animals were shown the playback of the cursor positions using their previous task performance (hereafter referred to as the passive playback condition). Activity in these higher areas was not evident in animals passively watching the cursor, suggesting that their recruitment was specific to goal-oriented task engagement (Figure 4). A separate cohort of animals trained using an auditory feedback cursor had variable task-active areas (Figure S4A, n = 4 mice), but, as with the visual task, higher activity was also evident in RL, which is a multimodal associative area with neurons responsive to visual, touch, and auditory stimuli (Mohan et al., 2018).

### Population tuning of neurons shifted toward target position

**Figure 5:**
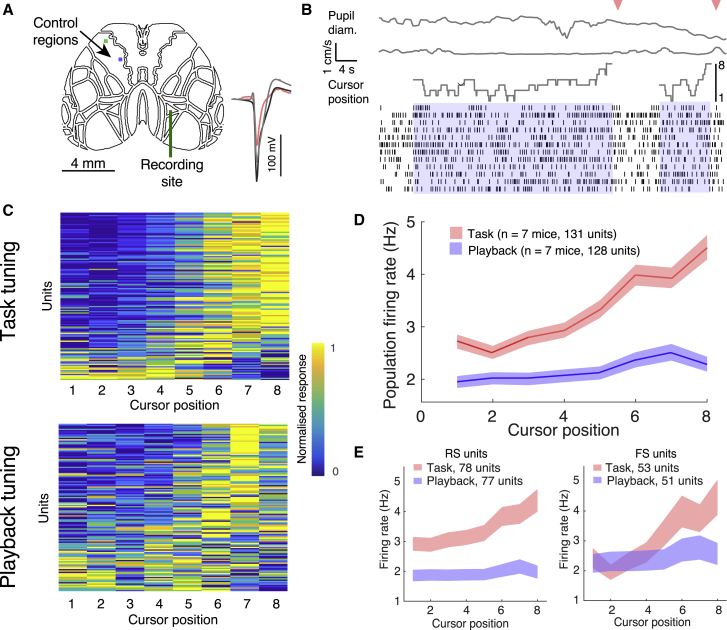
Cursor tuning shifts to target location (A) Electrophysiological recordings were targeted to AM while animals were performing the imaging-based BMI task, with control regions in the anterior motor cortex. Inset shows example waveforms from 3 isolated units (fast spiking unit in red). (B) Example spiking during 2 successful trials (trials denoted in blue, hits denoted with pink arrows). At top, traces, from top to bottom, are pupil diameter, running velocity, and visual cursor position. (C) Normalized tuning to cursor positions for all single units. Each row represents the normalized firing responses to each of 8 cursor positions for every recorded unit in the task (top, N = 131 units) and playback (bottom, N = 128 units) conditions. Firing responses were taken as the average firing rate for a period from 80 to 200 ms from the onset of the cursor presentation. (D) Average population firing rates for each cursor position during task performance (red) and passive playback (blue). Shaded regions indicate 95% confidence levels. (E) Left: mean firing rate for regular spiking (RS) units to different cursor positions during task performance (95% confidence interval indicated by shading, n = 7 mice). Right: mean firing rate for fast spiking (FS) units to different cursor positions during task performance (95% confidence interval indicated by shading, n = 7 mice).

**Figure 6:**
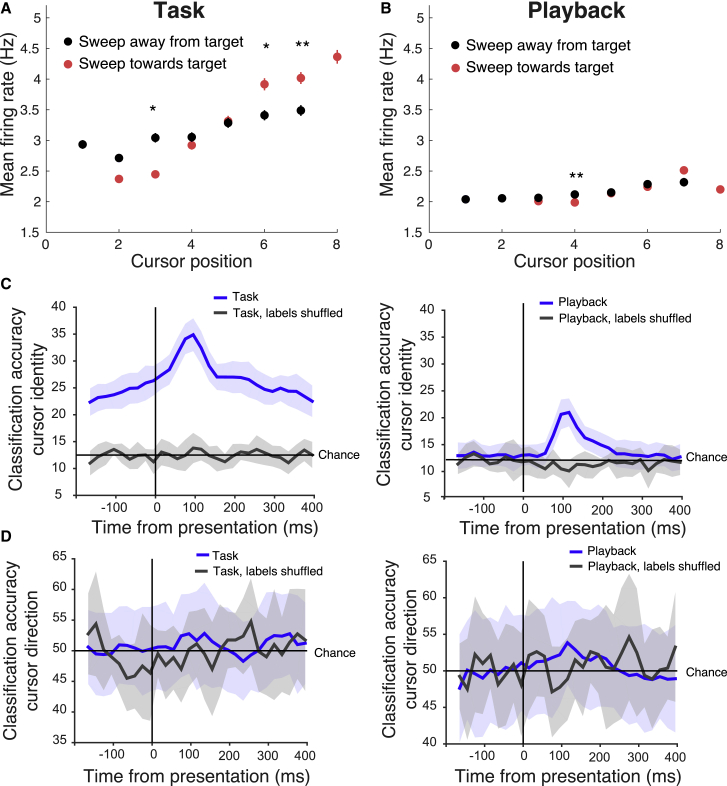
Cursor identity was more decodable from neural activity during task performance (A) Mean firing rate to different cursor positions depended on whether the preceding cursor position was sweeping toward (red) or away (black) from the direction of the target during task (t test, Bonferroni corrected, ^∗^p < 0.05 and ^∗∗^p < 0.01; error bars indicate SEM; n = 131 units). (B) Cursor sweep direction had little effect on firing rates when animals were passively viewing playback (t test, Bonferroni corrected, ^∗∗^p < 0.01; error bars indicate SEM; n = 128 units.) (C) Classification accuracy for cursor identity, from population responses trained on real (blue) and shuffled data (gray), for neural responses during task performance (left panel) and passive playback (right panel). Shaded regions denote 95% confidence interval (n = 7 mice). During task performance, the classifier could infer the upcoming cursor position even before presentation (chance level at 12.5%), and rose higher after presentation, suggesting that neural responses encode expectation. This was not true of the passive playback condition, in which case the classifier only performed above chance during the cursor presentation period. (D) Classification accuracy for cursor direction, from population responses trained on real (blue) and shuffled labels data (gray), for neural responses during task performance (left panel) and passive playback (right panel). Shaded regions denote 95% confidence interval (n = 7 mice). A trained classifier could not perform above chance (50%) in predicting, from firing alone, the direction that the visual cursor was moving, in either the task or playback conditions.

Having identified brain areas implicated in BMI control, we recorded spiking from individual units while animals performed the task, to investigate the task-dependent increase in calcium signals with cellular resolution. We chose to record from functionally identified area AM, due to its recruitment over learning, and used multi-channel silicon probes to record spikes from individual neurons while simultaneously imaging the rest of the dorsal cortex (Figure 5A; see Method Details; Xiao et al., 2017; Clancy et al., 2019; Barson et al., 2020; Peters et al., 2019). We obtained 16–49 single units per recording, spanning all cortical layers. Units could be classified as regular-spiking (RS) or fast-spiking, putative interneurons (FS), depending on spike width (see Method Details; Figure 5A). We recorded from 131 units in 7 mice performing the task (example recording shown in Figure 5B), and 128 units from the same animals passively viewing a playback of the previous training session’s cursor positions. Due to electrode shift over the recording sessions, we do not have recordings of the same units across both conditions, thus our analyses are limited to population response differences. During the task, population firing was significantly increased for cursor positions closer to the target, and this was true both of FS and RS units (Figures 5C–5E and S5A–S5C). The target-dependent increased spiking could not be explained by reward expectation, as a subset of animals were also given rewards at the target position during passive playback (Figure S5C). Responses were dependent on the preceding cursor position—firing to the cursor positions closest to the target was higher if the cursor swept toward the target, but lower if the cursor swept away from it. The opposite was true for the cursor positions farther from the target: firing was higher if the cursor swept away from the target (Figures 6A and 6B).

We surmised that the heightened neural responses around the target location could reflect a strengthening of relevant sensory feedback, disambiguating neural activity representing different cursor positions, and making the cursor identify more interpretable-by-recipient areas. This may allow downstream areas to more effectively decode the cursor position for better behavioral performance. To test this idea, we performed a classifier analysis on the population neural responses to confirm whether this was the case (Meyers, 2013). The cursor identity was much more effectively decoded from AM neural responses during task performance versus playback (Figure 6C). During the task, the classifier performed above chance (12.5%) even before the cursor was present, suggesting that the neural responses also encode intention or expectation. In the passive playback condition, the classifier only performed above chance after the cursor had been presented. However, the classifier failed to decode the travel direction of the cursor (toward or away from the target) in either the task or playback conditions (Figure 6D). Thus, the cursor direction-dependent difference in neural activity may reflect a role other than improving the decodability of the cursor movement direction—reflecting some interplay of the animal’s expectations and goal-direction (e.g., whether the cursor was moving in the direction the animal expected and/or intended).

Pupil diameter and running velocity were significantly decorrelated during task performance compared to playback (Figure S5F), suggesting that distinct mechanisms underlie pupil size modulation in the two conditions. At the population level, firing rates were uncorrelated with both pupil diameter and pupil position during the task, while firing rates were weakly correlated with pupil diameter during playback (Figures S5G and S5H). The fluorescence activity of the control regions was also uncorrelated with running velocity and pupil diameter (Figures S5I and S5J).

To understand the relationship between the firing rate of individual neurons and dorsal cortex-wide activity, we correlated the spike trains of individual units with fluorescence activity across the brain to build affiliation maps for each unit (Xiao et al., 2017; Clancy et al., 2019; Barson et al., 2020; Peters et al., 2019). We aligned these maps to the common coordinate framework of the Allen Brain Atlas using stereotaxic marks on the skull and sorted these maps by units’ preference for different cursor locations. During task performance, the cells most responsive to the target and target-adjacent cursor position were significantly more correlated with activity across the dorsal cortex (Figure S6A). This could mean that the boosting around the target may be the result of a cortex-wide signal, or that units tuned to the target correlate more strongly with the rest of dorsal cortex during task performance, but not playback (Figures S6B and S6C).

## Discussion

The idea of representation is fundamental to the idea of computation, and the neocortex appears to use hierarchies of transformed representations. It has become increasingly clear that cortical sensory representations are not strictly veridical reproductions of the outside world but are shaped by a subject’s internal states and goals. We sought to understand how having causal control over an external object affects the cortical sensory representation of that object, given that fluent control must be informed by a constant dialog between action and sensation (Haggard, 2017). Animals learned to causally control an external object using neural calcium signals recorded by widefield imaging and did so by discovering and exploiting experimentally defined mappings between their neural activity and the visual feedback that led to reward. This technique enabled us to identify cortical areas involved in task performance and then to target recordings from individual cells in these areas while animals were engaged in the task. We found that higher visual cortical areas, including area AM, were recruited during expert BMI control, and that single units in AM encoded the same visual cursor differently, depending on whether the animal was causally controlling it or passively viewing it. These results lend evidence to the idea that activity in AM, a potential homolog of the parietal cortex, encodes a subject’s intention and self-monitoring of sensorimotor transformations (Andersen and Buneo, 2002; Andersen and Cui, 2009; Desmurget et al., 2009; Aflalo et al., 2015; Cui, 2016).

Previous work indicates that subjects can learn to control neuroprosthetic devices using single cells or bulk electrophysiological signals (Fetz, 1969; Bakay and Kennedy, 1998; Nicolelis, 2001; Serruya et al., 2002; Carmena et al., 2003; Weiskopf et al., 2003; Sitaram et al., 2007; Koralek et al., 2012; Hochberg et al., 2012; Collinger et al., 2013; Clancy et al., 2014; Sadtler et al., 2014; Prsa et al., 2017; Sitaram et al., 2017; Trautmann et al., 2019), but this is the first work, to our knowledge, to apply control using population calcium signals. This technique allowed us to monitor much of the dorsal cortical network as animals learned neuroprosthetic control, whereas previous BMI work has been limited to recording from neighboring neurons. This lends evidence to the idea that manipulating neuroprosthetic devices using aggregate population signals (e.g., from infrared imaging or ultrasound; Shevelev, 1998; Abdelnour and Huppert, 2009), rather than electrophysiological recordings from individual neurons, may afford more stable and minimally invasive control that is robust to losing signals from or damage to individual control cells.

To learn the arbitrary action-outcome relationships required to perform BMI tasks, animals must match internally generated actions or activity with their sensory consequences. To probe how animals learned these contingencies, we changed the regions that controlled the BMI between and within training sessions, meaning that animals could not rely on a habitual activity pattern or strategy, but had to continually explore different neural patterns to achieve reward on different training days. Animals did so by “exploring” with variable neural activity patterns early in a training session, until they discovered a successful activity pattern to exploit (Figure 3). Target hit rates dropped when the reward was dispensed randomly, unlinked to the target zone, indicating that the animals’ task performance was goal directed and not habitual.

The spontaneous activity correlations between the control regions were predictive of the animals’ fluency of performance, which is instructive for BMI design considerations (Figure 3C; Sadtler et al., 2014; Clancy et al., 2014; Oby et al., 2019). Animals could modestly decorrelate normally correlated brain areas during task execution, although spontaneous activity was widely correlated across the cortex, as seen in prior work (Ledochowitsch et al., 2013). We found this to be true of both anterior and posterior cortical areas—in mice trained to control an auditory cursor, for example, posterior visual control areas could also be decorrelated during the task (Figure 3B). Pupil and locomotion also became decorrelated during task performance, indicating that task engagement and locomotion may engage distinct arousal mechanisms (Figure S5; Vinck et al., 2015; Reimer et al., 2016; Clancy et al., 2019).

By imaging dorsal cortex as animals performed this task, we were able to screen for cortical areas engaged during expert BMI control. On the first day of training, V1 was most active during the task, but as animals learned the task over days, higher visual areas, including the AM, PM, and RL, became more active as animals controlled the cursor. When the same cursors were played back to animals in an open-loop fashion (e.g., not controlled by animals), activation was again mainly evident in V1, suggesting that these higher areas were involved in the goal-directed aspect of task performance. AM and RL are considered by some to be rodent homologs of the parietal cortex (Glickfeld and Olsen, 2017; Wang et al., 2011), which has been shown in humans to be involved in intention and monitoring the mapping between action and outcome (Andersen and Buneo, 2002; Desmurget et al., 2009; Andersen and Cui, 2009; Aflalo et al., 2015; Cui, 2016). However, it was unclear whether the recruitment of these higher visual areas over learning was related to the fact that they are involved in sensorimotor transformations generally or because they are involved more specifically in planning and intentional control.

We targeted extracellular recordings in one of the functionally identified task-active areas, AM, to probe task-related changes in the spiking of single units. We found that units were more active during task performance than passive cursor playback of the animal’s recent performance. In particular, units were more responsive to the cursor when it was at the target and target-adjacent positions during the BMI task compared to passive playback, similar to the spatial attention boosting evident in previous work (Moran and Desimone, 1985; Engel et al., 2016), and in accordance with evidence that attention can reshape stimulus representations in a manner that more effectively guides decisions (Ruff and Cohen, 2019). A classifier trained on neural responses to the different cursor positions could effectively classify responses to the visual cursor and could do so with better accuracy when the animal was actively engaged in the task versus passively viewing a playback of the same cursor. In the task condition, the classifier ramped up in accuracy even before the cursor was presented, suggesting that neural responses were prospective, but whether this represents an intention (e.g., a control signal) or an expectation (e.g., a passive prediction) remains to be determined in future work. This boosting did not reflect reward expectation, and the animal did not use saccades or overt movements to perform the task. This boosting was also sensitive to the task goal: if the cursor was positioned close to the target and sweeping toward it, responses were boosted relative to when it was sweeping away. If the cursor was far from the target and sweeping away from it, then responses were also boosted relative to the cursor at the same position sweeping toward the target. This suggests that firing rates reflect intention and may also reflect a valence of the animal’s perceived fluency of cursor control—that is, whether it was successfully guiding the cursor toward its intended goal (Lee and Dan, 2012).

We present a novel task and imaging method for exploring the encoding of action-outcome assessments, which allowed us to simultaneously monitor—with both dorsal cortex-wide and cell-level resolution—what activity patterns support the causal control of external objects. This is a promising proof-of-principle for exploring the potential and limitations of future imaging- and population-activity-based BMIs. Imaging-based BMIs can also serve as a useful paradigm for studying the cortical dynamics involved in flexible learning, affording experimental control of what is learned and by what areas or molecularly defined cell types. This makes it an excellent system to probe how the brain adjusts to sensory and rule manipulations through altering activity patterns within and across areas. Future work could more systematically examine questions including how effectively animals can decorrelate different brain areas depending on underlying anatomical networks. Another promising avenue is credit assignment: BMI affords one less “translational” step in the mapping of neural activity and its sensory consequences than traditional motor learning paradigms. When animals learn a new motor pattern, this is accompanied by changes in neural activity that researchers must first identify (finding where the changes occur and what they are) and then show that this activity is necessary for the new behavior. With BMI learning, researchers can prescribe what neurons are learning what patterns, and also identify exactly when the new pattern emerges and when it is driving the rewarded behavior. For instance, rather than studying how neurons infer credit in limb movements, which result from brain activity across multiple areas, in BMI learning, defined activity patterns of a small group of cortical cells is directly rewarded, reducing the complexity of the problem.

While using wide-field imaging afforded us a view of the dorsal cortex as animals learned neuroprosthetic control, there are a number of limitations to this method. While wide-field fluorescence signals largely reflects neural firing, there is also a contribution from neuropil and hemodynamic effects that were uncorrected in this study, as in our previous work we found it did not significantly affect our findings (Clancy et al., 2019). However, there is clearly some component of the signal contributed by blood vessels (e.g., in Figure 3B), so this is a limitation of the study. Another limitation is that wide-field imaging is limited to recording from the dorsal cortex. Cortical neuroprosthetic control requires interactions with basal ganglia (Koralek et al., 2012; Neely et al., 2018), from which we cannot record using this method. Furthermore, we know from work in humans that the prefrontal cortex (PFC) is involved in the sense of control over external stimuli, but we cannot record signals from the PFC using this preparation due to its obscuration by the frontal sinus.

While we found increased evoked spiking to a visual cursor in the target location using this preparation, we do not know the exact cellular or neuromodulatory mechanisms giving rise to this difference. We found task-related increases in activity emerge over learning specifically in AM, PM, and RL, and are not present during passive visual playback or when visual feedback was randomized. However, as we only recorded neuronal responses in AM, we do not know whether the changes in neural tuning in AM are specific to this area, nor whether they are specifically required for task performance. Future work may address this by using mesoscale 2-photon imaging to record from or manipulate activity in molecularly defined neural subpopulations in parietal and control areas during task performance.

## STAR★methods

### Key resources table

**Table:** 

REAGENT or RESOURCE	SOURCE	IDENTIFIER
**Experimental Models: Organisms/Strains**		

Mouse: TRE-Gcamp6s	Jackson labs	JAX 024742; RRID: IMSR_JAX:024742
Mouse: B6.CBA-Tg(Camk2a-tTA)1Mmay/DboJ mice	Jackson labs	JAX 007004; RRID: IMSR_JAX:007004

**Software and Algorithms**		

Allen Brain Atlas API	Allen Institute	http://help.brain-map.org/display/api/Allen+Brain+Atlas+API
MATLAB 2015	MATLAB	https://uk.mathworks.com/help/matlab/release-notes-R2015b.html
Labview 2012	National Instruments	https://www.ni.com/en-gb/support/downloads/software-products.html
OpenEphys	Siegle et al., 2017	https://open-ephys.org/store
Klustakwik	Schmitzer-Torbert et al., 2005	https://github.com/klusta-team/klustakwik
Mapped Tensor (MATLAB)	Muir and Kampa, 2015	https://uk.mathworks.com/matlabcentral/fileexchange/29694-better-memory-mapped-files-in-matlab

### Resource availability

#### Lead contact

Further information and requests for resources and reagents should be directed to Kelly Clancy, the lead contact (k.clancy@ucl.ac.uk).

#### Materials availability

This study did not generate new unique reagents or mouse lines.

#### Data and code availability

The data and code used in this study are available from the lead contact upon reasonable request.

### Experimental model and subject details

#### Mice

All experimental procedures were carried out in accordance with institutional animal welfare guidelines and licensed by the Swiss cantonal veterinary office. TRE-Gcamp6s mice (Wekselblatt et al., 2016) (Jackson Laboratories, RRID: IMSR_JAX:024742) were crossed with B6.CBA-Tg(Camk2a-tTA)1Mmay/DboJ mice (Jackson Laboratories, RRID: IMSR_JAX:007004), to drive the expression of gCamp6s in CamKII+ pyramidal neurons. Animals were housed in a facility using a reversed light cycle, and recordings were taken during their active period. Eleven female mice were trained on the task, and we took electrophysiological recordings from seven of these, ranging between P55-P75. All animals were healthy, had never undergone previous procedures, and ranged in weight from 15-19 g. Sample sizes were not statistically determined, but were consistent with previous papers using related methodology (Clancy et al., 2019; Xiao et al., 2017). Animals were group housed with same-sex littermates in enriched environments (including running wheels, cardboard tubes and chewing toys).

### Method details

#### Surgery

A week before training, mice were prepared for imaging. Animals were anaesthetised with a mixture of fentanyl (0.05 mg per kg), midazolam (5.0 mg per kg), and medetomidine (0.5 mg per kg). The animal’s scalp was resected and a head plate was secured to the skull. Four stereotaxically placed marks were made to enable alignment of the imaged brain with the Allen Brain Atlas (http://mouse.brain-map.org/static/atlas) post hoc, using the Allen Brain API (http://help.brain-map.org/display/api/Allen+Brain+Atlas+API). The exposed skull was cleaned and covered with transparent dental cement to avoid infection, and to cover the cut scalp edges (C&B Metabond). This was polished to enhance the transparency of the preparation. A custom-made 3D printed light shield was cemented to the skull and head plate to avoid light leaks from the visual feedback presented on two computer monitors.

#### Behavioral setup and recordings

The recording chamber was sound-isolated and shielded from outside light. Mice were head-fixed under the microscope and free to run on a Styrofoam running wheel (diameter = 20 cm, width = 12 cm). The animals’ movements were recorded using a rotary encoder in the wheel axis (pulse rate 1000, Kubler). Two monitors were placed side by side in front of the mouse, angled toward one another (21.5” monitors, ∼20 cm from mouse, covering ∼100x70 degrees of visual space), similarly to the setup described in Poort et al. (2015). A reward port was place in front of the animal, where reward delivery was triggered via pinch solenoid one second after target hit (NResearch) and animal licks were detected using a custom piezo element coupled to the spout. All behavioral data were recorded using custom MATLAB software and a PCI-6320 acquisition board (National Instruments).

On electrophysiological recording days, pupil recordings were taken by illuminating the animal’s right eye with a custom IR-light source and imaging with a CMOS camera (DMK22BUC03, Imaging Source, 30 Hz) using custom MATLAB software. Pupil size was determined as described in Orsolic et al. (2019): images were first smoothed with a 2-D Gaussian filter and thresholded to low luminance areas. These thresholded regions were then filtered by circularity and size to automatically detect the pupil region. Pupil edges were detected using the canny method, and ellipses were iteratively fit to the region, tasked to minimize the geometric distance between the area outline and the fit ellipse using nonlinear least-squares (MATLAB function fitellipse, Richard Brown). The pupil diameter was taken to be the major axis of the ellipse, then normalized by animal. Pupil recordings from one animal had to be discarded, as the video was not sufficiently in focus.

#### Behavioral training

After recovery, mice were acclimatised to head fixation for a minimum of two days, and started on food restriction. Awake animals were head-fixed under the microscope and free to run on a Styrofoam wheel. A baseline of spontaneous activity was taken on every training day (10-20 minutes) in order to estimate spontaneous hit rates. The decoder was calibrated such that animals achieved ∼25% performance on their first day. Two small control regions were chosen for real-time read out. In the case of visual feedback task, these were all located in primary and secondary motor cortex, avoiding ALM. In the auditory feedback task, control regions were placed in posterior cortex, over visual and retrosplenial areas. The placement of the two control regions was usually ipsilateral but sometimes contralateral to each other. The same control regions were used for the first few days of training, then changed from day to day, or within sessions, so that animals did not learn a fixed control strategy (see Table S1).

Activity was imaged at 40 Hz and the mean fluorescence from each control region was transmuted to the cursor’s position on screen with a simple transform:

(Equation 1) $$p\left(t\right)=\;A_{1}F_{R1}-A_{2}F_{R2}+B$$

where p is the cursor position at time t, F_R1_ and F_R2_ are the instantaneous fluorescence (ΔF/F) of control regions one and two, respectively, and A_1_, A_2_ and B are coefficients set based on the daily spontaneous baseline recordings (minimum 10 minutes). P was rounded to the nearest integer to determine the discrete cursor location. A1 and A2 were determined by dividing the full dynamic range of each recorded area during the baseline by half the number of cursor positions:

(Equation 2) $$A_{1}=\;\left[max\left(F_{R1}\right)-min\left(F_{R1}\right)\right]/4;=\;\left[max\left(F_{R2}\right)-min\left(F_{R2}\right)\right]/4;$$

B represents the activity baseline of both areas. The chance performance was then assessed by running the baseline data through the decoder to estimate how often the animal would have achieved the target with spontaneous activity.

ΔF/F was calculated using a moving baseline, set as the tenth percentile of points from the preceding 20 s of data. The raw fluorescence was converted to ΔF/F using a moving baseline of 5 minutes of activity. The display updated at approximated 10 Hz, with a latency of 300 ms from camera to screen, measured using a photodiode placed on one of the monitors (Thorlabs, PDA100A-EC). Activity in R1 would cause the cursor to move toward the target location in the center of the animal’s visual field, while increased activity in R2 would cause the cursor to move away from the target zone. The cursor was presented on two monitors so that the animal could track the cursor with both eyes; its goal was to move the cursors presented on the two screens on either side to the middle of its visual field. These changes were binned, such that the cursor could take one of eight possible locations on the screen. The cursor had to be held at the target position for 0.3 s to count as a hit, at which point the cursor disappeared. When a target was hit, a MATLAB-controlled Data Acquisition board (National Instruments, Austin, TX) triggered the administration of a soyamilk reward following a 1 s delay. The next trial could be initiated within 5 s of reward delivery, but only when the activation of R1 relative to R2 returned to the mean value recording during spontaneous activity (to ensure enough time had passed for large transients to decay, given slow calcium dynamics). This was return to baseline condition was rarely triggered (∼5% of trials) and on average lasted under 2 s. If the animal did not bring the cursor to the target within a 30 s trial, the cursor disappeared, and the animal received a white noise tone and a 10 s ‘time out.’

We trained a separate cohort of four mice using an auditory, rather than visual, feedback cursor, where activity was transmuted to the pitch of a feedback tone (Clancy et al., 2014). As with the visual feedback task, a spontaneous baseline was recorded every day (10-20 minutes) to assess chance levels of performance and calibrate the decoder. Activity from two arbitrarily chosen regions was entered into an online transform algorithm that related neural activity to the pitch of an auditory cursor:

(Equation 3) $$f\left(t\right)=\;A_{1}e^{F_{R1}1}-A_{2}e^{RF_{R2}}+B$$

Where f is the cursor frequency, F_R1_ is the instantaneous ΔF/F of R1, F_R2_ the instantaneous ΔF/F of R2, and A1, A2, and B are coefficients set based on the daily baseline recording. As above, ΔF/F was calculated using a moving baseline, set as the tenth percentile of points from the preceding 20 s of data. Linear changes in firing rate resulted in exponential changes in cursor frequency, and frequency changes were binned in quarter-octave intervals to match rodent psychophysical discrimination thresholds. As with the visual task, a trial was marked incorrect if the target pitch was not achieved within 30 s of trial initiation. The auditory feedback was played using speakers mounted on 2 sides of the imaging platform.

#### Task and control conditions

In the task condition, the position of the presented cursor was determined by the control regions’ instantaneous activity, according to Equation 1 above. Rewards were given when the cursor hit the target zone (cursor position 8). Similarly, in the random reward condition, intended to test whether animal’s task engagement was goal-directed or habitual, the position of the presented cursor was determined by the control regions’ instantaneous activity, according to Equation 1 above. However, here rewards were not linked to the cursor position, but given out at random time intervals at a rate matched to an expertly performing animal (approximately 1.5 rewards/minute). In the random feedback condition, intended to confirm that the animal was using the visual feedback to inform its behavior, the position of the presented cursor was not linked to the control regions’ instantaneous activity, but instead was drawn randomly from a Gaussian distribution matching the mean and variance of a typical task condition. The animal could still receive rewards if they achieved the correct neural activity pattern, but their performance drop suggests they could not achieve that in the absence of appropriately linked sensory feedback. In the passive playback condition, the presented cursor position was no longer linked to the control regions’ activity, but was purely a replay of the cursor positions from a training session the animal had undergone previously. Thus, the cursor positions and timing of trials in the playback condition were matched to those of the task condition. Because the statistics of the sensory presentations between the task and playback conditions (e.g., cursor position identity, and likelihood of transition between different positions) were identical, this allowed a cleaner comparison of neural responses in these conditions.

#### Widefield imaging

Widefield imaging was performed through the intact skull using a custom-built epifluorescence macroscope with photographic lenses in a face-to-face configuration (85mm f/1.8D objective, 50mm f/1.4D tube lens; Ratzlaff and Grinvald, 1991). Data were recorded using a CMOS camera (Pco.edge 5.5, PCO, Germany) in global shutter mode. 16-bit images were acquired at a rate of 40 Hz and binned 2x2 online using custom-made LABVIEW software. A constant illumination at 470 nm was provided (M470L3, Thorlands, excitation filter FF02-447/60-25), with average power ∼0.05 mW/mm2 (emission filter 525/50-25, Semrock). The imaging site was shielded from light contamination using a 3D-printed blackout barrier glued to the animal’s skull. Signals from the two control regions were sent via UDP to a computer providing visual or auditory feedback to the mouse, using custom MATLAB software.

#### Electrophysiological recordings

The day before recording, mice were anesthetised with isofluorane and a small craniotomy was opened over AM, which was functionally identified during task performance, and stereotaxically confirmed. The craniotomy was kept damp with Ringer’s solution and sealed with KwikSil (World Precision Instruments). Recordings were taken on the following day to avoid residual effects of anesthesia.

On the recording day, animals were head-fixed under a custom-built widefield microscope, the skull and cortex were cleaned with Ringer’s solution, and the KwikSil plug removed from the craniotomy. A custom-designed silicon probe (64 channels, 2 shanks, Neuronexus, as described in Clancy et al., 2019) was inserted at an angle of ∼45 degrees from normal to cortex. The probe consisted of two shanks with 64 sites total, organized into 16 ‘tetrodes’, each consisting of 4 sites located 25 um apart from each other within-tetrode, and tetrodes spaced 130 um apart from each other. A small amount of KwikSil or agar was used to cover the exposed cortex after the probe was in place. After allowing the probe to settle for 20-30 minutes, neural activity was recorded using the OpenEphys recording system (Siegle et al., 2017). Behavioral and stimulation data, including pulses representing each camera frame, were recorded using OpenEphys, enabling the alignment of electrophysiological signals with imaging and behavioral data. Ephys recordings were filtered between 700 and 7000 Hz, and spikes detected using the Klustakwik suite (Schmitzer-Torbert et al., 2005). Clusters were assigned to individual units by manual inspection, excluding any units without a clear refractory period. Units were separated into fast and broad spiking units by their peak-to-trough time, using a cutoff of 0.66 ms (Barthó et al., 2004).

#### Data analysis

Raw imaging data were checked for dropped frames, spatially binned 2x2, and loaded into MATLAB as a mapped tensor (Muir and Kampa, 2015). The raw fluorescence was converted to ΔF/F using a moving baseline, calculated as the tenth percentile of points from the preceding 20 s of data. We did not perform hemodynamic correction as previous work indicates that hemodynamic and flavoprotein signals contribute minimally compared to the calcium responses (Vanni and Murphy, 2014; Xiao et al., 2017; Clancy et al., 2019).

Task-activation maps were calculated by taking the normalized average of fluorescence movies during the task, or visual cursor playback period, subtracted by periods when animals were not performing the task or viewing any visual stimuli (including periods of spontaneous activity, and reward collection). To ensure that differences between early and late in training were not influenced by possible differences in the statistics of the visual feedback cursor, we randomly excluded success trials on late training days in order to have comparable numbers of success and failure trials between early and late training, however, including or excluding these trials did not influence the result. To build the single-unit affiliation maps (Figure S6; see also Clancy et al., 2019), spike trains were binned to match imaging frames, and maps were calculated by taking the correlation of each unit’s spike train with each pixel’s ΔF/F.

Spectral entropy was calculated in 10 s windows, each overlapping by 5 s. The calcium signal of the control areas was transformed into power spectral density (PSD) during these windows (the magnitude squared of the signal’s Fourier transform). This was then used to calculate the spectral entropy for that time span:

$$SE=-\sum_{f=0}^{f=\frac{f_{s}}{2}}PSD_{n}\left(f\right)log_{2}\left[PSD_{n}\left(f\right)\right]$$

Where SE is the spectral entropy, PSD_n_ is the normalized PSD, and *f_s_* is the sampling frequency.

#### Classifier analysis

Spike data were binned into 50 ms bins, and split into 40 segments for training: 39 of these splits were used for training the classifier and 1 was used for testing. The data were z-score normalized so that high firing rate units didn’t bias the classifier results. This data was then used, along with the cursor position labels, to train the classifier: a mean vector was created for each cursor position class based on the training data, and predictions were made on the test data by choosing the label class with the maximum correlation between the test and training mean vectors. These predicted labels were compared with the true labels to generate an average classifier accuracy over each tested time bin. This process was repeated 20 times using different training/test splits to cross-validate the results. The final reported classification accuracy is the mean of these 20 runs.

### Quantification and statistical analysis

All data were analyzed using custom code in MATLAB. The statistical tests used in our analyses are indicated in the figure legends, which also includes the value of n, and whether it refers to animals or single units. Differences were tested using Student’s t test, and Bonferroni corrected where apropriate. P values are reported in the figures as well as legends, and significance is herein defined as p less than 0.05. The pupil video for one animal, taken on the final day of recording, had to be excluded as it was too out of focus to determine the pupil size. Sample sizes were not statistically determined, but were consistent with previous papers using related methodology (Clancy et al., 2019; Xiao et al., 2017). Data were assumed to be normal but this was not systematically tested. Animals were trained in three separate cohorts (the first, a cohort of four animals trained on the auditory feedback task, the second, a cohort of 5 animals trained on the visual feedback task, and the third, a cohort of 2 animals trained on the visual feedback test) as an internal replication.

## Supplemental information

Document S1. Figures S1–S6 and Table S1

Document S2. Article plus Supplemental Information
