# Supplementary material for: The sensory representation of causally controlled objects
Source: Neuron. 2021 Feb 17;109(4):677–689.e4. doi: 10.1016/j.neuron.2020.12.001 (PMC7889580; doi:10.1016/j.neuron.2020.12.001)
Supplement: Document S1. Figures S1–S6 and Table S1 [file mmc1.pdf]

Neuron, Volume *109*

Supplemental Information

The sensory representation of causally controlled objects

Kelly B. Clancy and Thomas D. Mrsic-Flogel

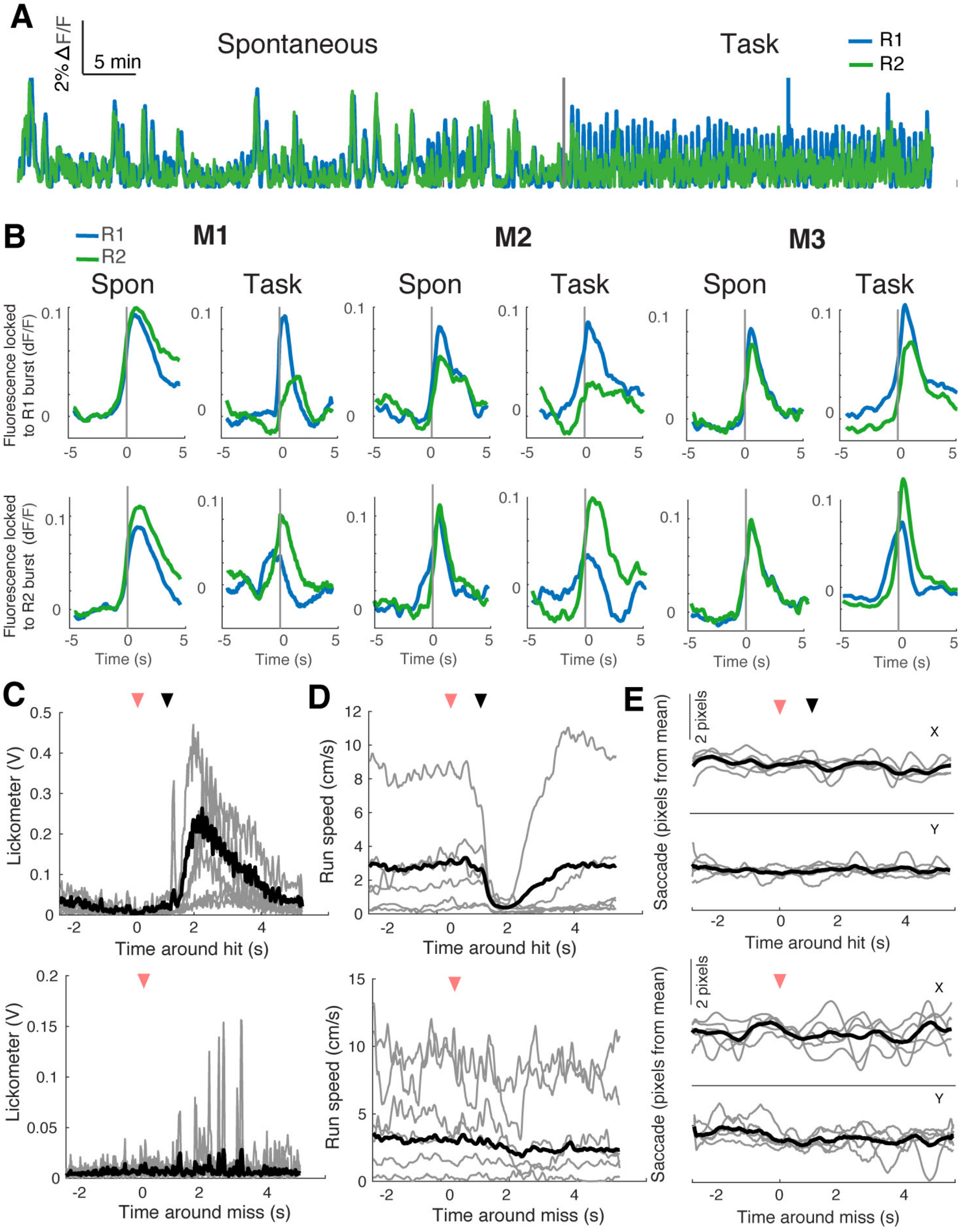

Supplemental Figure 1. Animals did not use gross motor movements to perform task (related to Figure 1)

A. Example fluorescence trace from R1 and R2 in pre-task spontaneous activity, and during task, indicating a clear change from spontaneous patterns. **B.** Analysis of activity throughout the task indicated that activity in the two control regions generally became decoupled during task performance, and not just around hits. Average ΔF/F in the control regions triggered by bursts in R1 (top row) or R2 (bottom row), for the same three example animals, for spontaneous activity (left) and during the training session (right). The burst threshold was taken as the z-scored value of ΔF/F > 3, in order to capture

large events. **C.** Lick averaged around hits (top panel) and misses (bottom panel) on a day in late training (n = 7 mice, grey traces indicate individual mice, black trace indicates mean). Pink arrow indicates target hit (top panel) or time out (bottom panel), black arrow indicates reward delivery (top panel). **D.** Animal's running speed, averaged around hits (top panel) and misses (bottom panel) on a day in late training (n = 7 mice, grey traces indicate individual mice, black trace indicates mean). Pink arrow indicates target hit (top panel) or time out (bottom panel), black arrow indicates reward delivery (top panel). **E.** Eye saccades averaged around hits (top panel) and misses (bottom panel) on final day of training (n = 6 mice). Average movement is shown for both x and y directions of pupil image. Pink arrow indicates target hit (top panel) or time out (bottom panel), black arrow indicates reward delivery (top panel).

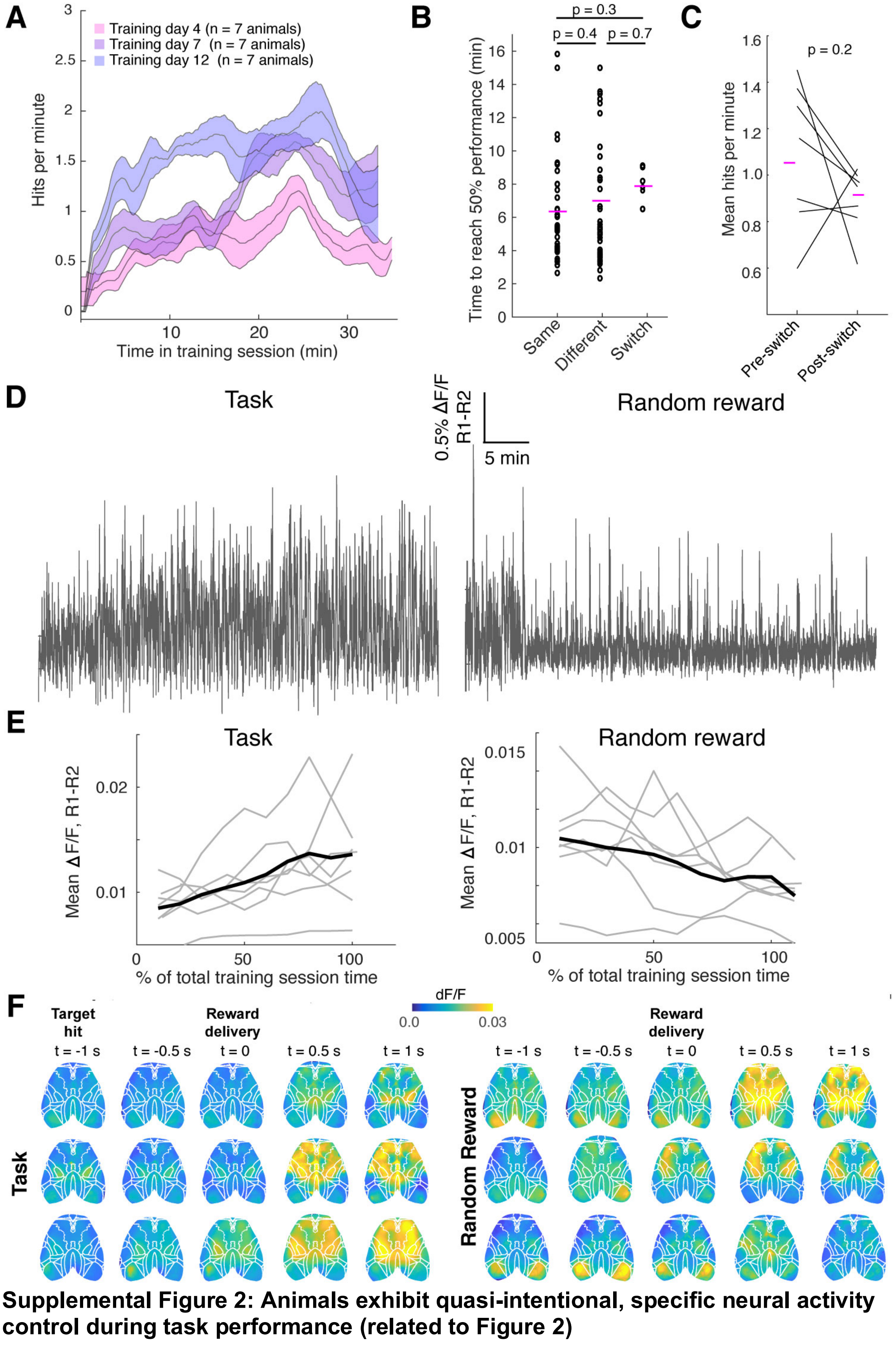

Supplemental Figure 2: Animals exhibit quasi-intentional, specific neural activity control during task performance (related to Figure 2)

A. Hit rate over the course of a training session averaged over 7 mice for three training days (shaded area indicates s.e.m., n = 7 animals for each day). **B.** Once animals were performing above chance (from day 4 on), there was no significant difference in the time it took animals to reach 50% performance whether they were using the same ROIs as the previous day of training, or different ones (t-test, p = 0.4). On day 6, when ROIs were switched mid-session, there was also no significant difference in the time it took, post-switch, for the animal to recover their performance to 50% compared to animals using the same or different control regions to the day before (t-test, p=0.3, 0.7 respectively, each dot indicates performance on one training day). **C.** On the day control regions were switched mid-session, there was no significant difference in performance between animals before their control regions were switched, and their recovery after the switch. Each line represents one mouse (n = 7 mice). **D.** Example trace of the difference between control areas' activity during task (R1-R2) for one mouse, indicating an increase in differential modulation over the course of a training session (left). These modulations decreased when reward was provided randomly (right). **E.** The slope of the difference in the two control regions fluorescence over training was positive during the normal task condition (left, $p < 0.01$, light-weighted lines each represent one of n = 7 mice, heavy line represents mean) and negative over the course of the randomised reward condition (right, $p < 0.01$, light-weighted lines each represent one of n = 7 mice, heavy line represents mean). **F.** (Left) Example activity maps locked around reward delivery for three animals performing the task. One second before the reward delivery marks the target hit, after which the cursor disappeared during the 1 second wait time. After reward delivery, strong activity is evident in frontal areas when animals are licking and touching the spout. (Right) Example activity maps locked around reward delivery for three animals in the random reward condition. Rewards were given randomly, unlinked to target hits, so the visual cursor was usually present throughout the reward collection period. After reward delivery, strong activity is evident in frontal areas.

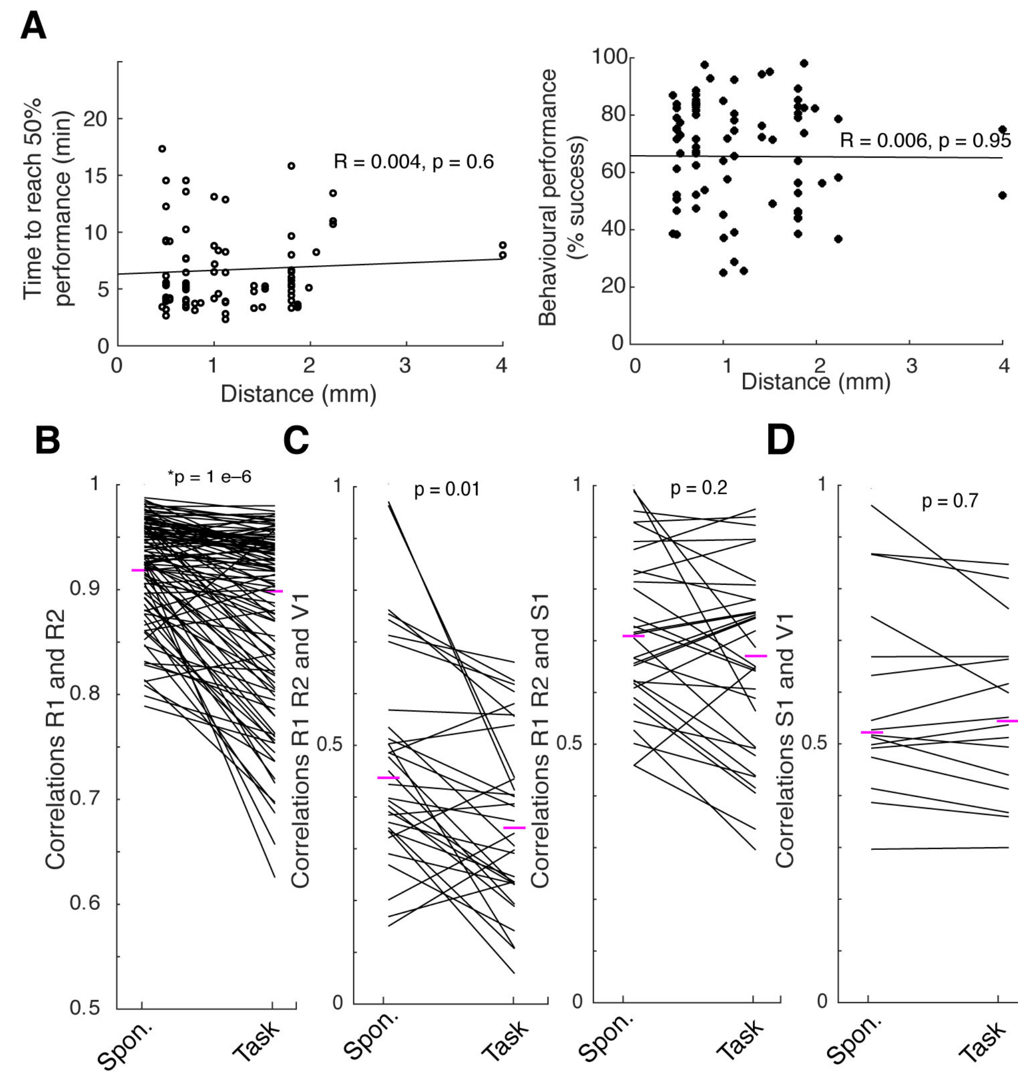

Supplemental Figure 3. Animals could modestly decorrelate areal brain activity during task performance (related to Figure 3)

A. (Left panel) The distance between control regions did not have an effect on the time it took animals to achieve 50% performance (for 83 training sessions, including all days of above chance performance from all 7 animals, e.g. from day 4 on). (Right panel) The distance between control regions did not have an effect on final performance (for 83 training sessions, including all days of above chance performance from all 7 animals, e.g. from day 4 on). **B.** Correlations between R1 and R2 dropped modestly from spontaneous levels during task performance (104 control region pairs from 7 animals, on all training days: on average 15 days of training per mouse, paired t-test, p = 1e-6). **C.** Left panel: correlations between control regions and activity in V1 dropped between spontaneous activity and task performance, but correlations between control regions and primary somatosensory cortex (S1) were not significantly different between conditions. Areas were identified by registering imaged brain surface with Allen Brain Atlas by stereotaxic marks; 7 animals, for expert level performance on days 9 and 14 of training paired t-test, p = 0.01, p=0.1, respectively for V1, S1). **D.** Correlations between primary visual cortex and primary somatosensory cortex did not change between spontaneous

activity and task performance (areas as identified by registering imaged brain surface with Allen Brain Atlas by stereotaxic marks; 7 animals, for expert level performance on days 9 and 14 of training paired t-test, $p = 0.02$).

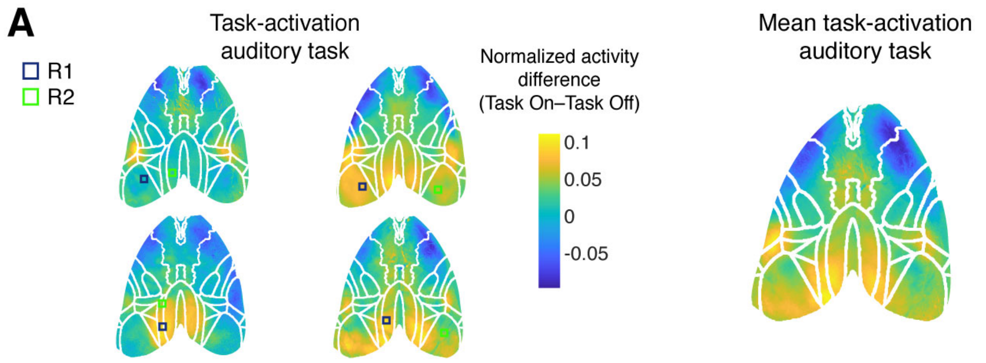

Supplemental Figure 4. Expert animals had increased activity in higher visual areas during task performance (related to Figure 4)

A. Left panel: example task-related activity maps from four animals trained on an auditory version of the task. Right panel: mean task-related activity map indicating involvement of RL, a lateral homolog of parietal cortex in mice and a multimodal associative area (mean from n = 4 mice). Visual areas appear task-activated (despite there being no visual feedback in this task) likely due to the fact that the control regions were placed over visual areas. Control regions are shown as slightly larger than they actually were for better visibility.

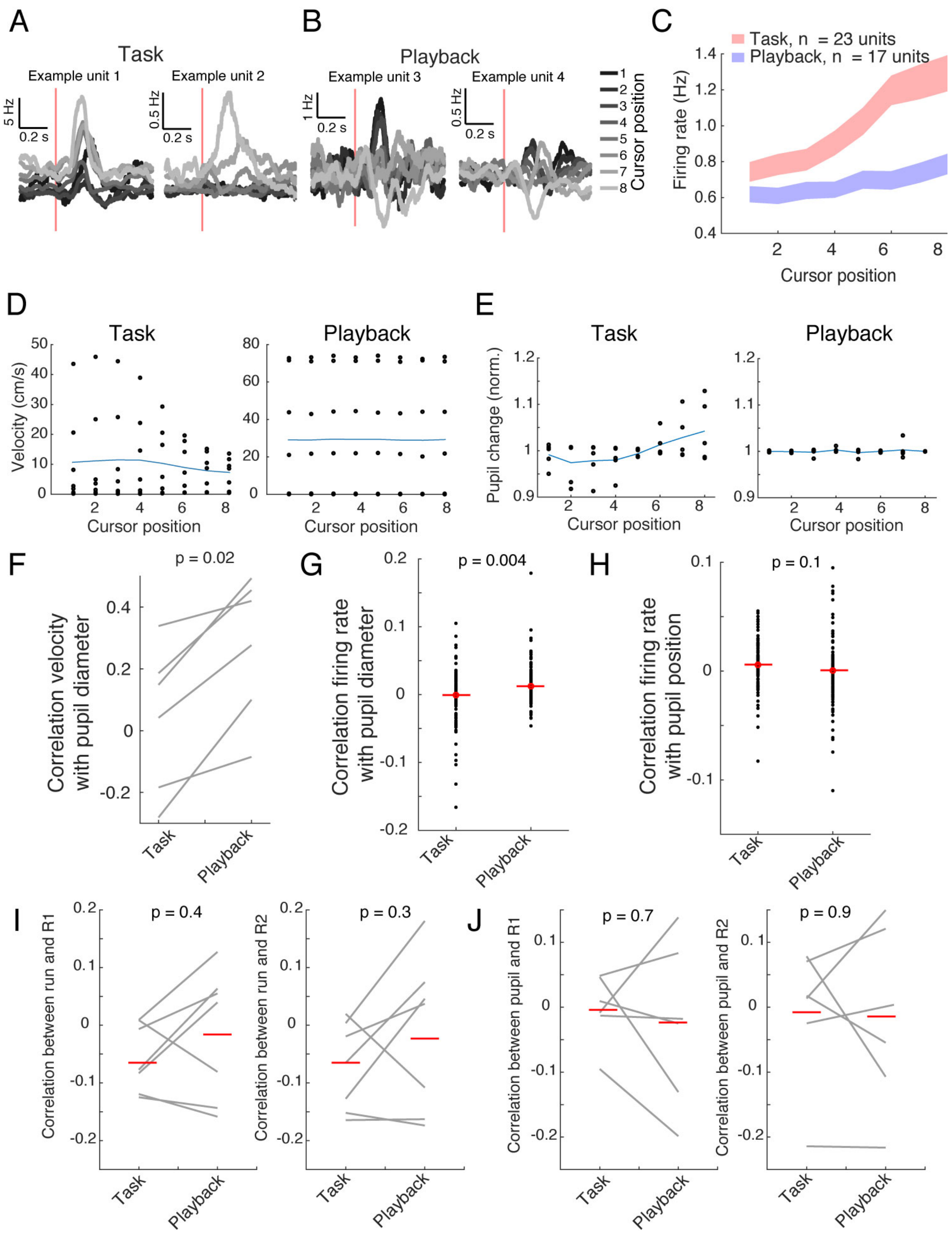

Supplemental Figure 5. Pupil and locomotion become uncoupled during task (related to Figure 5)

A. Responses to the visual cursor at the 8 monitor positions for two single units recorded during task performance. The red line denotes the time the cursor appeared; the trace colour denotes cursor position. Here the spiking responses have been smoothed by convolving with a gaussian kernel with standard deviation of 25 ms. **B.** Responses to the visual cursor at the 8 monitor positions for two single units recorded during passive

playback. The red line denotes the time the cursor appeared; the trace colour denotes cursor position. Here the spiking responses have been smoothed by convolving with a gaussian kernel with standard deviation of 25 ms. **C.** Firing rates for units recorded in 2 animals where the target cursor position was rewarded during passive playback (blue trace). The enhanced firing for the target-adjacent cursor positions during task performance (red trace) remained, suggesting the boosting was not simply reward expectation (95% confidence interval indicated, n = 2 mice, 23 units in the task condition and 17 units in the passive playback condition). **D.** Average running velocity at each cursor location during recordings during task (left) and playback (right), (n = 7 mice, final day of recording). **E**. Normalized pupil diameter at each cursor location during task (left) and playback (right), (n = 6 mice, final day of recording). **F.** Pupil diameter and running speed were significantly decorrelated during task performance compared to playback (reward collection periods and inter-trial intervals excluded, n = 6 mice, final day of recording, each line represents correlations for one mouse.) **G.** The correlation between single unit firing rate and pupil diameter was not different from zero during task, but slightly higher than zero during playback (reward collection periods and inter-trial intervals excluded, n = 6 mice, N = 105 units task, n= 122 units playback). **H.** The correlation between single unit firing rate and pupil position was not different from zero during task or playback conditions (reward collection periods and inter-trial intervals excluded, n = 6 mice, N = 105 units task, n= 122 units playback). **I.** R1 (left panel) and R2 (right panel) activity were not significantly correlated with running velocity, either during task performance or passive playback (reward collection periods and inter-trial intervals excluded, N = 7 mice, each line represents correlations for one mouse). The correlation between R1 or R2 and run velocity during task and passive playback were not significantly different from zero (t-test: for R1, p = 0.08, p=0.6, respectively for task and playback; for R2: p = 0.08, 0.7 respectively for task and playback), nor significantly different from each other (paired t-test, p = 0.4). **J.** R1 (left panel) and R2 (right panel) activity were not significantly correlated with pupil diameter, either during task performance or passive playback (reward collection periods and inter-trial intervals excluded, N = 6 mice, t-test; each line represents correlations for one mouse). The correlation between R1 or R2 and pupil diameter during task and passive playback were not significantly different than zero (t-test, for R1: p = 0.9, 0.7 for task, playback, respectively; for R2: p = 0.9, 0.8 for task, and playback, respectively), nor significantly different from each other (paired t-test, p = 0.4).

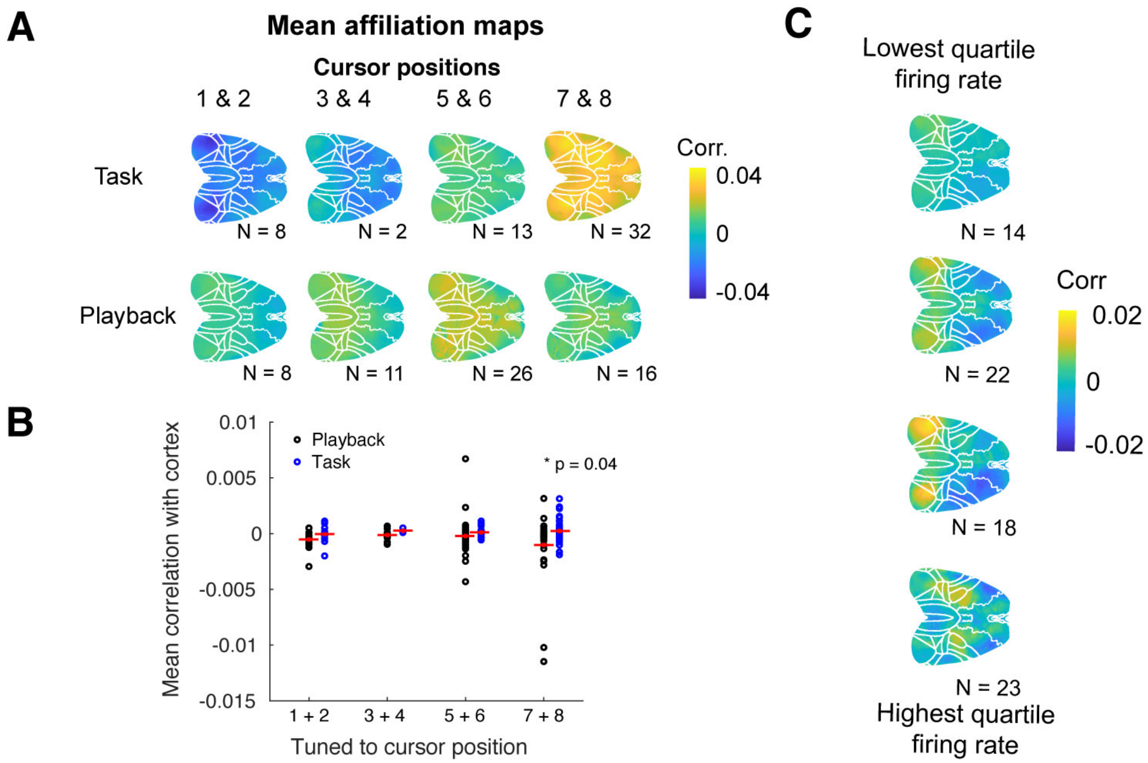

Supplemental Figure 6. Cortex-wide affiliations of units tuned to different cursor positions (related to Figures 5 & 6)

A. Spike trains for each unit were correlated with the activity of each pixel to build cortex-wide affiliation maps. These were sorted into bins based on which cursor position units were most responsive to. The average of these maps is shown in the top row for task performance, and the bottom row for passive playback. The reported N under each average map indicates the number of single units in that bin for that condition. Only units which had statistically significant tuning to a cursor location, and units with correlation maps that had >50% pixels with significant correlations (p values were Benjamini–Hochberg corrected for multiple comparisons, with a significance threshold set at a 5% false positive rate) were included. **B.** Mean correlation of spiking activity with calcium activity across dorsal cortex, sorted by units' cursor preference for task and playback. **C.** The trend evident in **A** is not due to higher firing rates of target cursor-tuned units, when units were organized into quartiles of mean firing rates rather than cursor preference. The reported N under each average map indicates the number of single units in that bin for that condition.

	[M/L, A/P] (mm)	**M1**	**M2**	**M3**	**M4**	**M5**	**M6**	**M7**
**Day 1**	**R1**	-1.5, 1.5	-2.5, 2	-2, 1.5	-2.5, 2	-2.5, 2.5	-2, 1.5	-1.5, 1
	**R2**	-2.5, 1.5	-2.6, 1	-2.2, 1	-2.5, 0.5	-2.5, 2	-2.5, 2.5	-2.5, 2
**Day 2**	**R1**	-1.5, 1.5	-2.5, 2	-2, 1.5	-2.5, 2	-2.5, 2.5	-2, 1.5	-1.5, 1
	**R2**	-2.5, 1.5	-2.6, 1	-2.2, 1	-2.5, 0.5	-2.5, 2	-2.5, 2.5	-2.5, 2
**Day 3**	**R1**	-1.5, 1.5	-2.5, 2	-2, 1.5	-2.5, 2	-2.5, 2.5	-2, 1.5	-1.5, 1
	**R2**	-2.5, 1.5	-2.6, 1	-2.2, 1	-2.5, 0.5	-2.5, 2	-2.5, 2.5	-2.5, 2
**Day 4**	**R1**	-1.5, 1.5	-2.5, 2	-2, 1.5	-1, 3	-2.5, 2.5	-2, 1.5	-1.5, 1
	**R2**	-2.5, 1.5	-2.6, 1	-2.2, 1	-2, 1.5	-2.5, 2	-2.5, 2.5	-2.5, 2
**Day 5**	**R1**	-1.5, 1.5	-2.5, 2.3	-2, 1.5	-2.5, 2	-2.5, 2.5	-1, 1	-2, 1.5
	**R2**	-2, 1.5	-2.5, 1.5	-2.2, 1	-2, 1.5	-2.5, 2	-2, 1.3	-1.5, 1.5
**Day 6****	**R1**	-1.5, 1.5	-2.5, 2.3	-2.5, 2.5	-2.5, 2	-2.5, 2.5	-1.5, 1	-2, 1.5
	**R2**	-2, 1.5	-2.5, 1.5	-2, 2	-2, 1.5	-2.5, 2	-2, 1.5	-1.5, 1.5
**Day 7**	**R1**	-1, 1.5	-2.5, 2.5	-2.5, 2.5	-2.5, 2	-2, 2.5	-2.5, 1.5	-2, 1.5
	**R2**	-1.2, 1.8	-0.5, 3	-2, 2	-1.5, 2.5	-1, 0.5	-2.5, 2	-1.5, 1
**Day 8**	**R1**	-0.8, 2.2	-1.7, 1.5	-1, 1.5	-2.5, 2	-2, 2.5	-2.5, 1.5	-2, 2
	**R2**	-1, 2	-1, 2	-1.5, 2	-1.5, 1.5	-1, 0.5	-2.5, 2	-2.5, 2
**Day 9**	**R1**	-1.2, 0.5	-2, 1.5	-1.5, 3	-2.5, 2	-2, 2.5	-2.5, 1.5	-1.5, 1.5
	**R2**	-2.2, 1	-1.5, 1	-1.5, 1.5	-1.5, 0.5	-1, 0.5	-2.5, 2	-2.5, 2
**Day 10**	**R1**	-1.5, 1	-2, 1.5	-2.5, 2.2	-2.5, 2	-2, 1.5	-2.5, 1.5	-1.5, 1.5
	**R2**	-1.2, 2	-1.5, 1	-1.5, 1.5	-1.5, 0.5	-1.5, 1.5	-2.5, 2	-1.7, 1.7
**Day 11**	**R1**	-1.5, 1	-2, 1.5	-2.5, 2	-2.5, 2	-2, 1	-2.5, 1.5	-1.5, 1.5
	**R2**	-1, +2	-1.5, 1	-1.5, 2	-1.5, 0.5	2, 1	-2.5, 2	-1.7, 1.7
**Day 12**	**R1**	-1.5, 1	-2.5, 2	-0.7, 0.5	-2.5, 2	-2, 1	-2, 1.5	-0.5, 0.5
	**R2**	-1, 1.5	-1.5, 1	-2, 2	-1.5, 0.5	2, 1	-2, 2.5	-1.5, 1.5
**Day 13**	**R1**	-1.5, 1	-1.2, 0.5	-1, 0.5	-1.5, 0.5	-2, 2	-1, 0.5	-0.5, 0.5
	**R2**	-1, 1.5	-1.5, 2	-2, 2	-2.5, 2	-1, 0.5	-2, 1	-1.5, 1.5
**Day 14**	**R1**	-1.5, 0.2	-1.2, 0.5	-1, 0.5	-1.5, 0.5	-1.5, 1.5		
	**R2**	-2, 2	-1.5, 2	-2, 2	-2.5, 2	-2, 2		
**Day 15**	**R1**	-1.5, 0.2	-1, 0.2	-1, 0.5	-1.5, 0.5	-1.5, 1.5		
	**R2**	-2, 2	-1.5, 2	-2, 2	-2.5, 2	-2, 2		
**Day 16**	**R1**		-1, 0.2	-1, 0.5	-1.5, 0.5	-1.5, 1.5		
	**R2**		-1.5, 2	-2, 2	-2.5, 2	-2, 2		
**Day 17**	**R1**					-1.5, 1.5		
	**R2**					-2, 2		

Supplemental Table 1. Control region coordinates (related to Figure 1)

Related to Figure 1B: approximate stereotaxic coordinates, in millimeters, of control regions on all days of training, reported in [medial/lateral, anterior posterior] pairs, with bregma = [0, 0], and coordinates to the left of, or posterior to, bregma reported as negative. Day 6, indicated with **, was the day that control regions were switched midway through the training session. The margin of error for each position is ~0.1mm.
