# Supplementary material for: The sensory representation of causally controlled objects
Source: Neuron. 2021 Feb 17;109(4):677–689.e4. doi: 10.1016/j.neuron.2020.12.001 (PMC7889580; doi:10.1016/j.neuron.2020.12.001)
Supplement: Document S2. Article plus Supplemental Information [file mmc5.pdf]

Neuron

Article

The sensory representation of causally controlled objects

Highlights

- Mice controlled a visual cursor using population neural activity in cortical areas
- Animals could decorrelate normally correlated cortical areas to perform the task
- Introduction of a new rule triggered stochastic exploration of activity patterns
- Controlled objects elicited more robust sensory responses in a goal-oriented manner

Authors

Kelly B. Clancy,
Thomas D. Mrsic-Flogel

Correspondence

k.clancy@ucl.ac.uk (K.B.C.),
t.mrsic-flogel@ucl.ac.uk (T.D.M.-F.)

In Brief

Clancy and Mrsic-Flogel investigate how the perception of a causally controlled object is different than one passively viewed. Mice learn to control a visual cursor using brain activity alone. Neural responses to the controlled cursor include more information about the present and future cursor positions than during passive cursor viewing.

Clancy & Mrsic-Flogel, 2021, Neuron *109*, 677–689
February 17, 2021 © 2020 The Author(s). Published by Elsevier Inc.
https://doi.org/10.1016/j.neuron.2020.12.001

CellPress

Neuron

CellPress
OPEN ACCESS

Article

The sensory representation of causally controlled objects

Kelly B. Clancy[1,2,3,*] and Thomas D. Mrsic-Flogel[1,2,*]
[1]Biozentrum, University of Basel, 70 Klingelbergstrasse, 4056 Basel, Switzerland
[2]Present address: Sainsbury Wellcome Centre, University College London, 25 Howland Street, London W1T 4JG, UK
[3]Lead Contact
*Correspondence: k.clancy@ucl.ac.uk (K.B.C.), t.mrsic-flogel@ucl.ac.uk (T.D.M.-F.)
https://doi.org/10.1016/j.neuron.2020.12.001

SUMMARY

Intentional control over external objects is informed by our sensory experience of them. To study how causal relationships are learned and effected, we devised a brain machine interface (BMI) task using wide-field calcium signals. Mice learned to entrain activity patterns in arbitrary pairs of cortical regions to guide a visual cursor to a target location for reward. Brain areas that were normally correlated could be rapidly reconfigured to exert control over the cursor in a sensory-feedback-dependent manner. Higher visual cortex was more engaged when expert but not naive animals controlled the cursor. Individual neurons in higher visual cortex responded more strongly to the cursor when mice controlled it than when they passively viewed it, with the greatest response boosting as the cursor approached the target location. Thus, representations of causally controlled objects are sensitive to intention and proximity to the subject's goal, potentially strengthening sensory feedback to allow more fluent control.

INTRODUCTION

How does the brain infer a causal relationship between its activity and the sensed world, and how does this affect the sensory encoding of controlled external objects? Actions and perceptions reciprocally affect one another in a mutual dialog (Dewey, 1896). The sense of control can be operationalized to inferring a causal relationship between a subject's internally generated actions or activity and their outcome in the external world (Haggard, 2017). In motor learning, for example, the relationship between an action and its outcome can be learned and relearned throughout adulthood as animals acquire new motor skills. Brain machine interfaces (BMIs) are a method for investigating how subjects learn arbitrary action-outcome relationships (Fetz, 1969; Bakay and Kennedy, 1998; Nicolelis, 2001; Donoghue, 2002; Carmena et al., 2003; Sitaram et al., 2017). When learning to control a BMI, rodents have been found to use the same mechanisms as implicated in motor learning (Koralek et al., 2012; Neely et al., 2018). However, unlike motor learning, wherein animals learn a task and researchers must search for correlates of the behavior in patterns of neural activity, BMIs allow the experimenter to precisely control sensory feedback and prescribe the requisite activity patterns necessary for successful task execution, which can then be changed day to day. Thus, animals learning neuroprosthetic control of external objects must engage in continuous self-monitoring to assess the contingency between their neural activity and its outcome, preventing them from executing a habitual or fixed motor pattern, and encouraging animals to learn arbitrary new sensorimotor mappings on the fly.

A key aspect of this self-monitoring is the sensory feedback from the object being controlled by the agent. However, little is known about how causally controlled objects are represented in the brain. Studies have implicated the parietal cortex in intention and in the subjective assessment of agency over outcome. In human subjects, disrupting activity in the parietal cortex temporarily ablates self-reported agency (Chambon et al., 2015). Parietal activity has been found to be involved in representing task rules, the value of competing actions, and visually guided real-time motor plan updating, both in humans (Pisella et al., 2000; Kahnt et al., 2014; Wisniewski et al., 2015; Zapparoli et al., 2020) and non-human primates (Andersen et al., 1997; Sugrue et al., 2004). Motor plans can be decoded from parietal activity, and its responses are task, expectation, and goal dependent, in humans, (Rushworth et al., 2001; Desmurget et al., 2009; Aflalo et al., 2015), non-human primates (Mountcastle et al., 1975; Gnadt and Andersen, 1988; Lacquaniti et al., 1995; Johnson et al., 1996; Churchland et al., 2008), and rodents (Licata et al., 2017; Pho et al., 2018; Mohan et al., 2018, 2019). All of this evidence suggests that, across multiple species, the parietal cortex plays a role in intentional, goal-directed behaviors (Rizzolatti et al., 1997; Andersen and Buneo, 2002; Andersen and Cui, 2009). However, previous studies of the role of the parietal cortex in intention have not examined how causal control affects sensory representations across different sensorimotor contingencies.

This is an open access article under the CC BY license (http://creativecommons.org/licenses/by/4.0/).

To address this, we devised a mouse model of adaptive causal control. Control is traditionally studied through the lens of motor actions, which makes comparing sensory responses across control and passive conditions difficult, given that the former involves movement-related signals. By using a BMI task, we minimized movement-related differences in the neural responses between the conditions. Animals learned to guide a visual feedback cursor to a target location to obtain a reward using neural activity in experimenter-defined cortical areas, recorded with wide-field imaging. This had the added benefit of acting as an unbiased screen to identify dorsal cortical areas involved in learning the task. We found that higher visual areas, including the anteromedial cortex (AM), were more engaged when expert animals controlled the BMI. These higher areas are considered by some to be a putative homolog of parietal cortex in mice (Harvey et al., 2012; Licata et al., 2017; Mohan et al., 2018; Pho et al., 2018; Lyamzin and Benucci, 2019; Gilissen et al., 2020). To gain insight into what this task-related activity looks like on an individual neuron level, we targeted single-cell recordings to the functionally identified task-relevant region AM, and found that the visual cursor elicited larger responses when an animal was controlling it in a closed-loop configuration than when passively viewing it in an open-loop configuration (Bagur et al., 2018). Responses were highest when the cursor was closest to the target zone and were sensitive to the cursor's instantaneous trajectory: they were greater when the cursor was moving toward the target than away from it. Thus, the sensory representation of the visual object was sensitive to the subject's intention and its perception of the object's instantaneous trajectory with respect to its goal. Given that animals had to relearn a changing sensorimotor contingency on the fly, we surmised that the heightened sensory representation of the cursor may serve to strengthen the signal to adjudicating areas for informing fluent control over external objects. The neural activity in AM during the task condition carried significantly more information about the cursor identity than during the passive playback condition.

RESULTS

Goal-directed control of a visual cursor using areal signals

To investigate how causal control over external objects is effected and encoded in mammalian cortex, we trained mice to control a visual feedback cursor using real-time calcium signals recorded with wide-field imaging (largely reflecting the summed spiking of local cells; Makino et al., 2017; Clancy et al., 2019). We imaged the dorsal cortex in transgenic mice expressing the calcium indicator GCamp6s in CaMKII^{+} pyramidal neurons (Wekselblatt et al., 2016), assigning two small frontal regions to control the cursor (Figures 1A and 1B; Videos S1, S2, and S3), similar to a task described previously (Clancy et al., 2014; Koralek et al., 2012). Animals were head-fixed under the wide-field microscope, and free to run on a styrofoam wheel. The animal's goal was to bring the cursor (a copy of which was presented to both eyes on two separate monitors flanking each side of the mouse) to a target position in the center of its visual field (Figure 1C). Animals could achieve this by increasing activity in control region 1 (R1) relative to control region 2 (R2). If activity in R2 was greater than R1, then the cursor moved toward the back of the animal's visual field. Thus, in this design, animals could not hit the target simply by generally increasing or decreasing activity across the cortex, but had to differentially modulate the activity of these specific regions (Figures 1D–1F, S1A, and S1B). The control regions were usually placed over ipsilateral motor areas and were changed from day to day (Figure 1B; Table S1). Control regions were deliberately small (~0.1 mm^2) as we reasoned that it would be easier for animals to control a smaller area, but this was not systematically tested. We avoided using the anterior lateral motor cortex (ALM) in the control regions, to minimize the effect of licking.

The visual feedback cursor could take one of eight positions on the monitors (Figure 1C), and the cursor had to be held at the target for 300 ms to count as a hit. When animals succeeded in holding the cursor at the target position, a soya milk reward was delivered after a 1-s delay. If animals failed to bring the cursor to the target position within 30 s, then the trial was a miss, and a white noise miss-cue was followed by a time out. Chance performance was assessed using spontaneous activity recorded before the task began and represented the estimated hit rate that the animal would have achieved using spontaneous fluctuations of neural signals alone. Mice improved their performance over training (Figure 2A; n = 7 mice) and took less time to reach a criterion performance of 50% hit trials over days (Figure 2B).

Animals could perform the task without overt movements, licking, or eye saccades (Figures S1C–S1E). Several recent papers have shown that many motor activities can influence cortical activity (Stringer et al., 2019; Musall et al., 2019; Orsolic et al., 2019; Salkoff et al., 2020), raising the possibility that mice may adopt a motor strategy to control the activation of cortical regions used for BMI. Our analyses show that animals could perform the task without gross overt movements that were detected by monitoring licking, eye saccades, or running speed (Figures S1C–S1E), although it is possible that subtler movement were not detected. This is a question common to all BMI studies that is ultimately unanswerable without recordings from every muscle in the body. We did not take body videos of the animals performing the task, but future work that included video monitoring would offer insights on subtler motor outputs. Nevertheless, animals in this study could discover arbitrary activation patterns of different brain areas within and between training sessions and use these activations to control a visual cursor in a manner dependent on sensory feedback: that is, they could flexibly work out the arbitrary coupling of action patterns (whether purely confined to the brain, or involving peripheral muscle contractions) with sensory feedback to achieve a goal. The flexibility with which the animals could readjust their control of arbitrary brain areas suggests some degree of adaptability in reducing correlations of otherwise highly correlated brain areas (see below), although this varied for different regions.

Control regions could be changed from day to day or within a session: hit rates improved over time within a session and recovered after control regions were changed, indicative of learning (Figures 2C, S2A, and S2B). After recovery, the animals' hit rates before and after the switch were unchanged (Figure S2C). The

CellPress
OPEN ACCESS

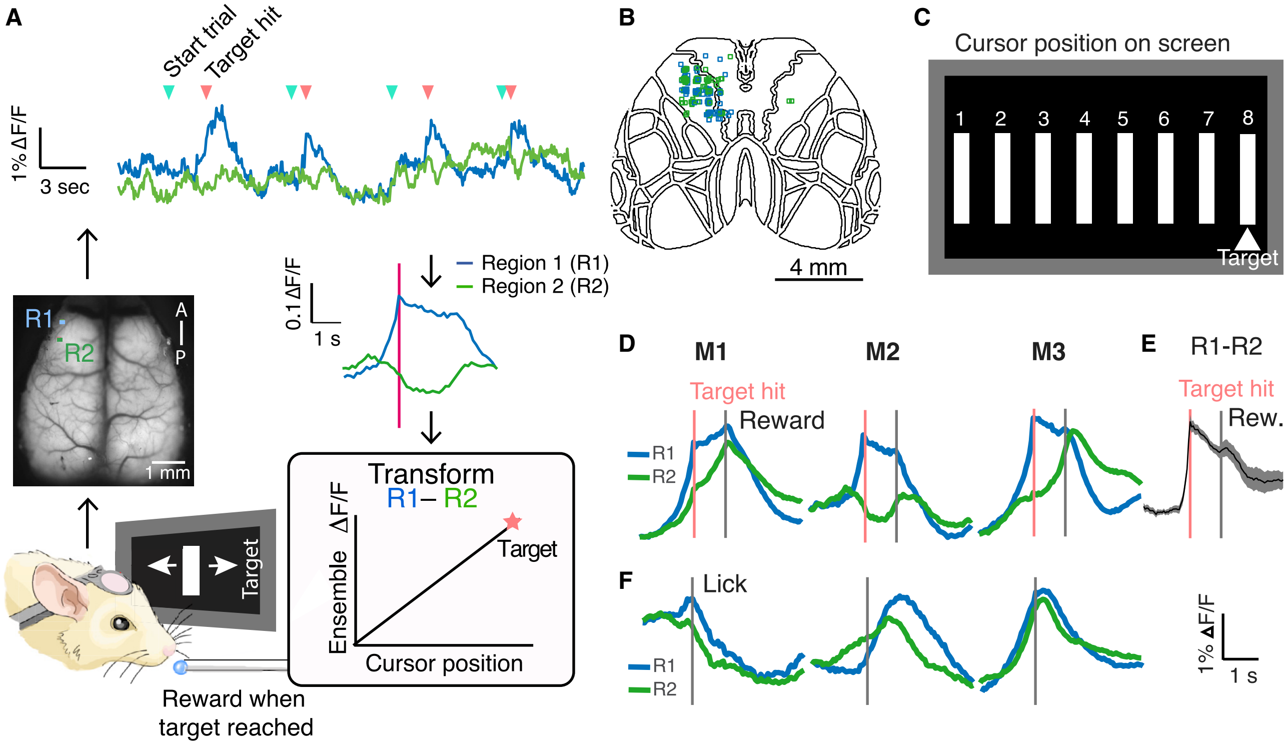

Figure 1. A widefield-imaging-based brain machine interface

(A) Task schematic. Clockwise starting from illustration of mouse: wide-field signals were imaged from head-fixed animals in real time and transmuted into the position of a visual cursor. Two small regions (R1 and R2) were used for controlling the cursor, and activity recorded from these areas was fed into a decoder such that their activity opposed one another. Example dF/F for the 2 regions is shown at top, with blue arrows denoting trial starts, and pink arrows denoting target hits. Activity averaged around hits for 1 example animal, 1 day, shown for R1 and R2. Animals had to increase activity in R1 relative to R2 to bring the cursor to a rewarded position at the center of their visual field, at which point they could collect a reward after a 1-s delay.

(B) Positions of control ROIs (R1 in blue, R2 in green) for all 7 animals over the course of training (averaging 15 days each), superimposed on the Allen Brain Atlas (totaling 104 pairs).

(C) Feedback schematic: the cursor could take 1 of 8 potential positions on screen, with position 8, the target, rewarded.

(D) ΔF/F in control regions triggered around hits for 3 example animals on 1 day of training, indicating different strategies that animals use to achieve reward. Pink line indicates the time of target hit and gray line indicates reward delivery.

(E) Activity in R1 subtracted by activity in R2, averaged around target hits for all mice on a day of training (n = 7 mice, shading represents SEM).

(F) Animals could not control cursor using lick alone. ΔF/F triggered around lick bouts in spontaneous activity for the same 3 example animals on 1 day of training, indicating animals could not achieve the differential activation of R1 and R2 using lick alone. Gray line indicates time of lick.

decoder transformation for both the pre- and post-switch conditions was calculated using the same spontaneous baseline taken before the training session. To test whether the behavior was goal directed, we dissociated the reward from the target position. On day 8 of training, animals were allowed to perform the task as usual, but the training session was constrained to 30 min. Thereafter, the visual feedback was coupled to the animal's neural activity as before, but rewards were given randomly, at the same rate as an expert animal engaged in the task ($\sim$1.3 hits/min). Given random rewards, the animals' target hit rate dropped to chance, indicating that target hits were goal oriented (Figure 2D). The fluorescence signals representing the difference between R1 and R2 increased over the normal training session, indicative of the increased efficacy of control and decreased when the reward was randomized, again suggesting that animals were effecting the requisite neural patterns in a goal-directed manner (Figures S2D and S2E). Animals were able to recover their performance following reinstatement of the normal task on the next day of training (Figure 2D). For a subset of animals, the visual feedback was then randomized: the visual cursor was presented at random positions, although animals could still achieve the target with the appropriate neural activity patterns. The animals' ability to bring the cursor to the target dropped to chance levels without meaningful visual feedback (Figure 2D). Activity leading to hits did not resemble activity induced by licking around reward collection, as evidenced in both the normal task and random-reward conditions (Figure S2F). This suggests that animals performed the task in a flexible, goal-directed, visual feedback-dependent manner.

Exploration and exploitation in neural activity space

As control regions were changed day to day, the activity patterns necessary for successful BMI control had to be re-learned each session. Example fluorescence traces from control regions

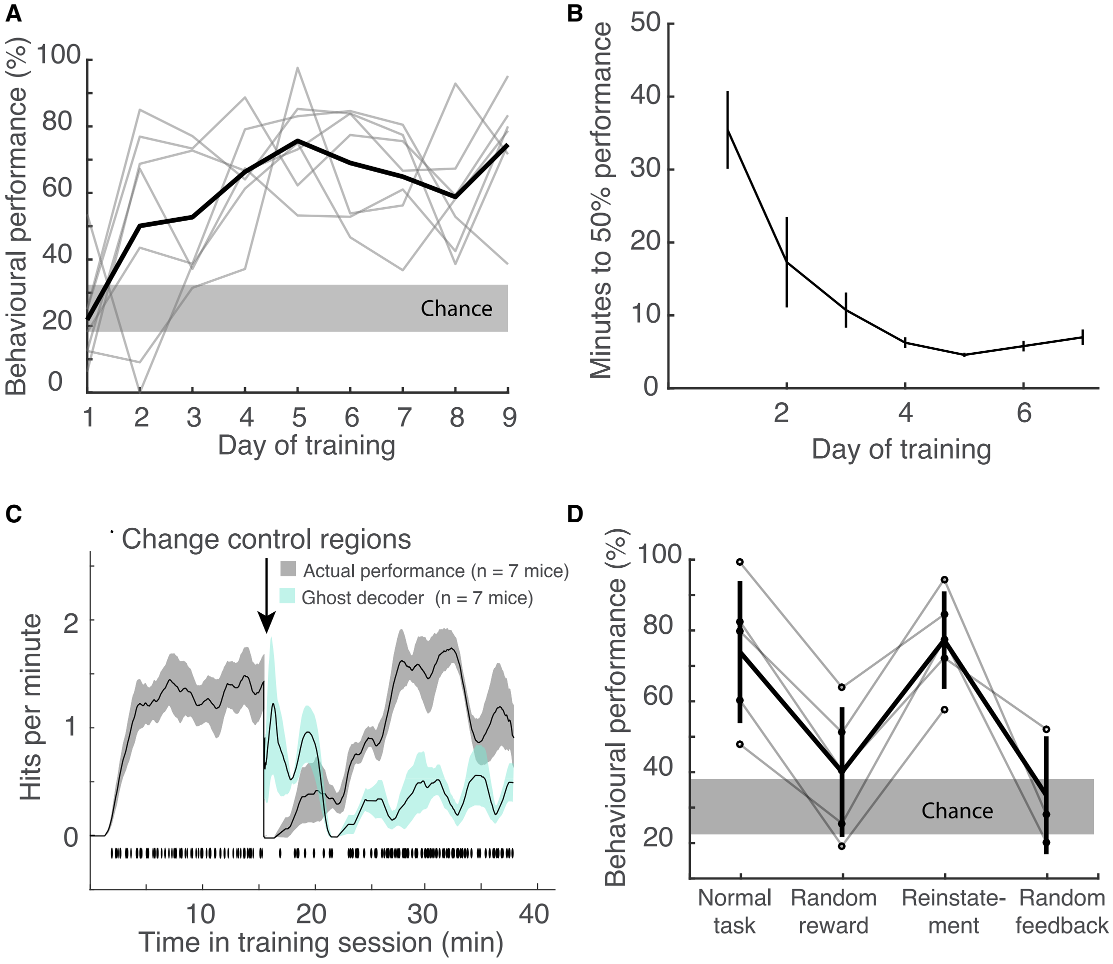

Figure 2. Animals learn to control a visual cursor using areal neural activity

(A) Behavioral performance (percentage of trials the animal successfully reached the target within the 30-s trial window) increased above chance over the course of days. Shaded region denotes chance SEM, assessed as how often spontaneous activity would achieve hits, averaged across 7 mice.

(B) Animals achieve 50% performance faster over the course of days of training, calculated by taking a moving average of the number of times the animal successfully reached the target per trial. Error bars denote SEM averaged across 7 mice.

(C) Average hits per minute (gray) increased over the course of a training session and recovered within minutes when control regions were changed (n = 7 mice on day 6 of training; shading denotes 95% confidence interval, see Table S1). Individual hit times, pooled for all animals, shown below rate curves. At chance baseline, animals should perform ~0.3 hits/min (here, animals started at slightly worse than chance, likely due to the fact that some of the control region positions had been changed from the previous day). After R1 and R2 were swapped mid-session, the estimate of how well the animals would have done using the original control regions is shown in teal: the performance of the previous ("ghost") decoder starts near the hit rate prior to the switch, suggesting that mice persist with their original strategy, but this drops off as animals discover the new rewarded contingency and recover their performance.

(D) Performance dropped to near chance when rewards were given randomly but recovered when target and reward was again coupled on a subsequent day (n = 5 mice). Performance also dropped to chance when the visual feedback cursor was uncoupled from neural activity and presented at random (n = 3 mice), even though animals could still achieve reward using the previously learned neural activity pattern (i.e., reward was no longer linked to cursor position). Error bars denote standard deviation. Shaded region denotes chance SEM, assessed as how often spontaneous activity would achieve hits (n = 7 mice).

indicate that the areas were initially highly spontaneously correlated (Figures 3A [top trace], S1A, and S1B; Video S3). Early in the training session, hits were preceded by diverse activity patterns (Figure 3A, center trace). By late in the training session, activity patterns leading to hits were more consistent (Figure 3A, bottom trace). Animals found different ways to achieve this consistency, sometimes by sweeping activity through R1 toward R2, or by depressing R2 while activating R1 (Figure 1D; Videos S1, S2, and S3).

To achieve the prescribed activity pattern, animals had to functionally decorrelate the two control regions, which were usually spontaneously correlated. Activity in the dorsal cortex was globally correlated pre-task, as indicated by correlation maps using R1 and R2 as seed pixels (Figures 3B and S1B). Correlations between these areas decreased during task performance (Figures 3B and S3B), as did the correlations between the control regions and the primary visual cortex, V1 (Figure S3C). Correlations between the control

CellPress
OPEN ACCESS

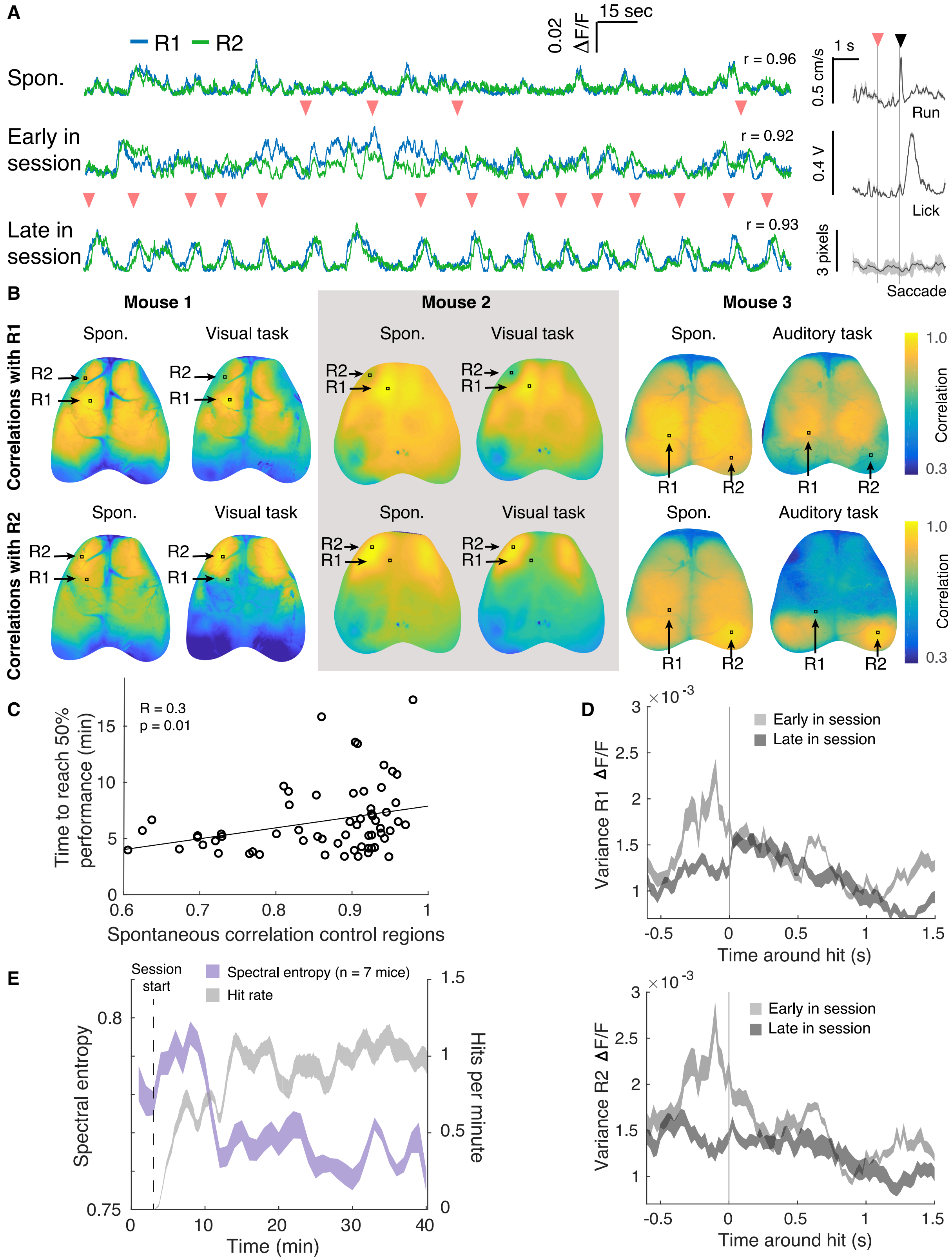

(legend on next page)

regions and the primary somatosensory cortex, S1, as well as between S1 and V1, were unchanged between spontaneous activity and the task (Figures S3C and S3D). Only periods of task performance, and not reward collection or inter-trial waiting periods, were included in these analyses. Animals could arbitrarily reduce correlations between different regions sufficient to perform the task. In a separate cohort of animals trained using auditory feedback instead of visual feedback, animals could also decorrelate visual control areas (Figures 3B, rightmost panel, and S4). Interestingly, the task-induced correlation patterns were invariably bilateral, even when control regions were ipsilateral to each other. Not every training session resulted in the kind of decorrelated maps evident in Figure 3B; reflecting that some areas are harder for the animals to decorrelate than others. Animals took longer to reach criterion 50% performance when control regions were spontaneously highly correlated (Figure 3C). The distance between control regions did not affect the time animals required to reach 50% performance (Figure S3A), suggesting that areas that were the most spontaneously correlated, irrespective of their distance from one another, were the hardest to use to perform the task.

The variance in R1 and R2 activity peaked around hits early in a training session as animals explored strategies that would yield reward, as has been shown previously for the activity of individual cells learning to control a BMI device using a fixed decoder (Zacksenhouse et al., 2007; Athalye et al., 2017). This variance decreased later in the session as animals discovered and exploited reliable, reproducible strategies (Figure 3D). Early in the session, the spectral entropy of activity (a measure of the spectral power distribution of a signal, and a proxy for its complexity; see Method details) around the control regions increased as animals explored activity patterns that would yield reward, then dramatically dropped as they discovered successful patterns and reliably exploited them (Figure 3E). At the start of a session, or when control regions were changed, animals faced with uncertain task rules "explored" activity space stochastically, and gradually switched to effecting stereotyped patterns they could reliably exploit after having probed the rules of their environment (Tervo et al., 2014).

Expert performance correlated with increased activity in higher visual areas

We sought to determine what cortical areas were most active during task performance, and how this changed over learning. On the first day of training, the primary visual cortex was most active during the task, but as animals became expert in the task, higher visual areas were recruited (Figures 4A–4C): in particular, AM, posteromedial cortex (PM), and rostrolateral visual cortex (RL), similar to previous studies of learning visually guided tasks in mice (Wekselblatt et al., 2016; Orsolic et al., 2019). Areas AM and RL are considered by some to be parietal cortex homologs in the mouse cortex, although there is no widespread agreement on this point (Harvey et al., 2012; Licata et al., 2017; Mohan et al., 2018; Pho et al., 2018; Lyamzin and Benucci, 2019; Gilissen et al., 2020). After the final day of training, animals were shown the playback of the cursor positions using their previous task performance (hereafter referred to as the passive playback condition). Activity in these higher areas was not evident in animals passively watching the cursor, suggesting that their recruitment was specific to goal-oriented task engagement (Figure 4). A separate cohort of animals trained using an auditory feedback cursor had variable task-active areas (Figure S4A, n = 4 mice), but, as with the visual task, higher activity was also evident in RL, which is a multimodal associative area with neurons responsive to visual, touch, and auditory stimuli (Mohan et al., 2018).

Population tuning of neurons shifted toward target position

Having identified brain areas implicated in BMI control, we recorded spiking from individual units while animals performed the task, to investigate the task-dependent increase in calcium signals with cellular resolution. We chose to record from functionally identified area AM, due to its recruitment over learning, and used multi-channel silicon probes to record spikes from individual neurons while simultaneously imaging the rest of the dorsal cortex (Figure 5A; see Method Details; Xiao et al., 2017; Clancy et al., 2019; Barson et al., 2020; Peters et al., 2019). We obtained 16–49 single units per recording, spanning all cortical layers. Units could be classified as regular-spiking (RS) or fast-spiking, putative interneurons (FS), depending on spike width (see Method Details; Figure 5A). We recorded from 131 units in

Figure 3. Exploration and exploitation of neural activity patterns

(A) Areal signals were highly spontaneously correlated before the training session (top trace). Animals explored different activation patterns early in the training session (center trace) to discover and exploit successful patterns by the end of the session (bottom trace). Pink arrows denote target hits. Pearson's correlation between R1 and R2 indicated on the right of each trace. Right: run, lick, and saccade averaged around hits for this training session; gray shading denotes 95% confidence interval around mean. Pink arrow denotes target hits, black arrow denotes reward delivery.

(B) Correlation map across cortex for 3 animals using activity in R1 (top row) and R2 (bottom row) as seeds, during spontaneous activity and during the BMI task. Mouse 3 was part of a separate cohort trained on an auditory task (see Method Details). Animals could decorrelate normally correlated areas for task performance.

(C) Animals took longer to reach criterion performance (50% hits/attempt) if control regions were highly spontaneously correlated (linear regression on data from 7 mice, 9 days of training starting on day 4).

(D) The average variance of activity for R1 (top panel) and R2 (bottom panel) was greater around hits early in a training session than late in the session (n = 7 mice, day 8 of training; shading indicates 95% confidence interval around mean), indicating that mice honed in on more reliable and reproducible control strategies over the course of a single training session.

(E) Early in the session, neural activity around the control regions had high spectral entropy (a proxy of signal randomness) as animals used stochastic bursts of activity to explore the neural patterns that would yield reward. By late in the session, animals had discovered a successful activity pattern to exploit, and spectral entropy in control area activity decreased. Shaded area indicates 95% confidence interval around mean.

CellPress
OPEN ACCESS

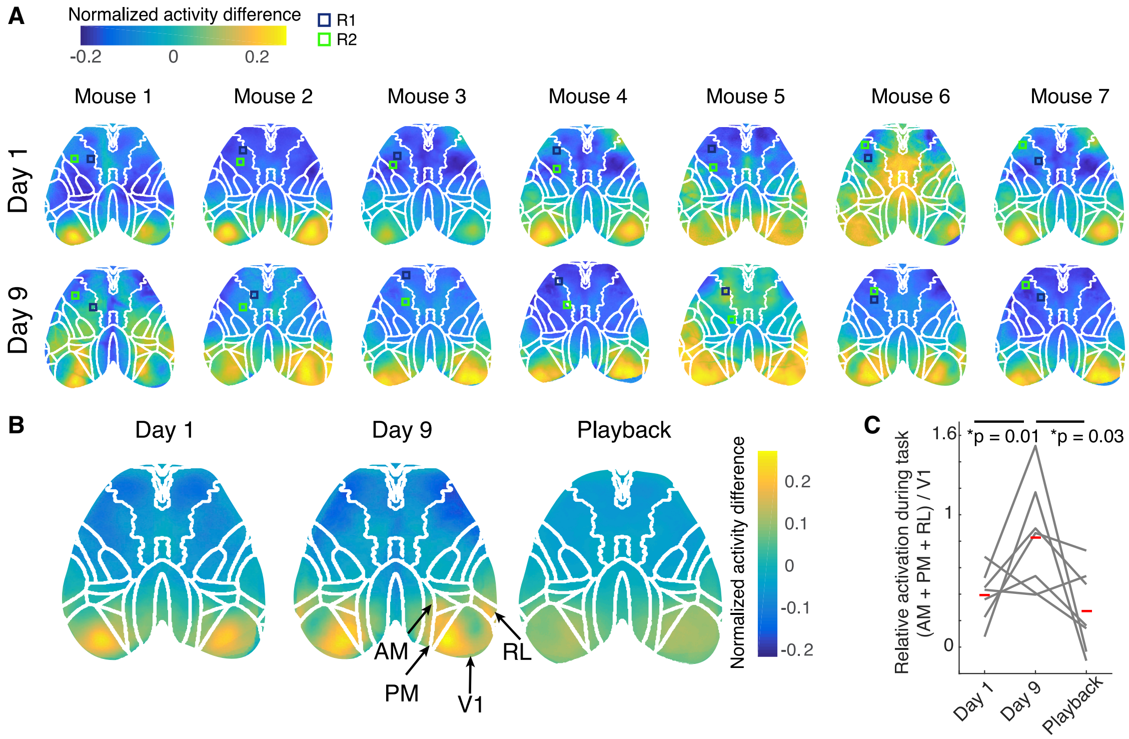

Figure 4. Higher visual areas were more active during expert task performance

(A) Activation maps for individual animals on day 1 of training (top row), when animals were naive, versus day 9 of training (bottom row), when animals performed the task expertly, calculated using the normalized activity difference for task-on minus task-off periods. Each map has been registered to the Allen Brain Atlas (overlaid) using stereotaxic marks. Control regions are shown as slightly larger than they actually were for better visibility.

(B) Activation maps during task performance on day 1, day 9, and during passive playback of a previous session (representing the normalized activity difference for task-on minus task-off periods). Each map has been registered to the Allen Brain Atlas by stereotaxic marks and then averaged across 7 mice.

(C) The relative ratio of task activation in higher visual areas versus V1 increased over training. When animals passively viewed playback of the same session, higher visual areas were less active. Red bars indicate mean ratios (n = 7 mice, paired t test, Bonferroni corrected).

7 mice performing the task (example recording shown in Figure 5B), and 128 units from the same animals passively viewing a playback of the previous training session's cursor positions. Due to electrode shift over the recording sessions, we do not have recordings of the same units across both conditions, thus our analyses are limited to population response differences. During the task, population firing was significantly increased for cursor positions closer to the target, and this was true both of FS and RS units (Figures 5C–5E and S5A–S5C). The target-dependent increased spiking could not be explained by reward expectation, as a subset of animals were also given rewards at the target position during passive playback (Figure S5C). Responses were dependent on the preceding cursor position—firing to the cursor positions closest to the target was higher if the cursor swept toward the target, but lower if the cursor swept away from it. The opposite was true for the cursor positions farther from the target: firing was higher if the cursor swept away from the target (Figures 6A and 6B).

We surmised that the heightened neural responses around the target location could reflect a strengthening of relevant sensory feedback, disambiguating neural activity representing different cursor positions, and making the cursor identify more interpretable-by-recipient areas. This may allow downstream areas to more effectively decode the cursor position for better behavioral performance. To test this idea, we performed a classifier analysis on the population neural responses to confirm whether this was the case (Meyers, 2013). The cursor identity was much more effectively decoded from AM neural responses during task performance versus playback (Figure 6C). During the task, the classifier performed above chance (12.5%) even before the cursor was present, suggesting that the neural responses also encode intention or expectation. In the passive playback condition, the classifier only performed above chance after the cursor had been presented. However, the classifier failed to decode the travel direction of the cursor (toward or away from the target) in either the task or playback conditions (Figure 6D). Thus, the cursor direction-dependent difference in neural activity may reflect a role other than improving the decodability of the cursor movement direction—reflecting some interplay of the animal's expectations and goal-direction (e.g., whether the cursor was moving in the direction the animal expected and/or intended).

Pupil diameter and running velocity were significantly decorrelated during task performance compared to playback (Figure S5F), suggesting that distinct mechanisms underlie pupil size modulation in the two conditions. At the population level, firing rates were uncorrelated with both pupil diameter and pupil

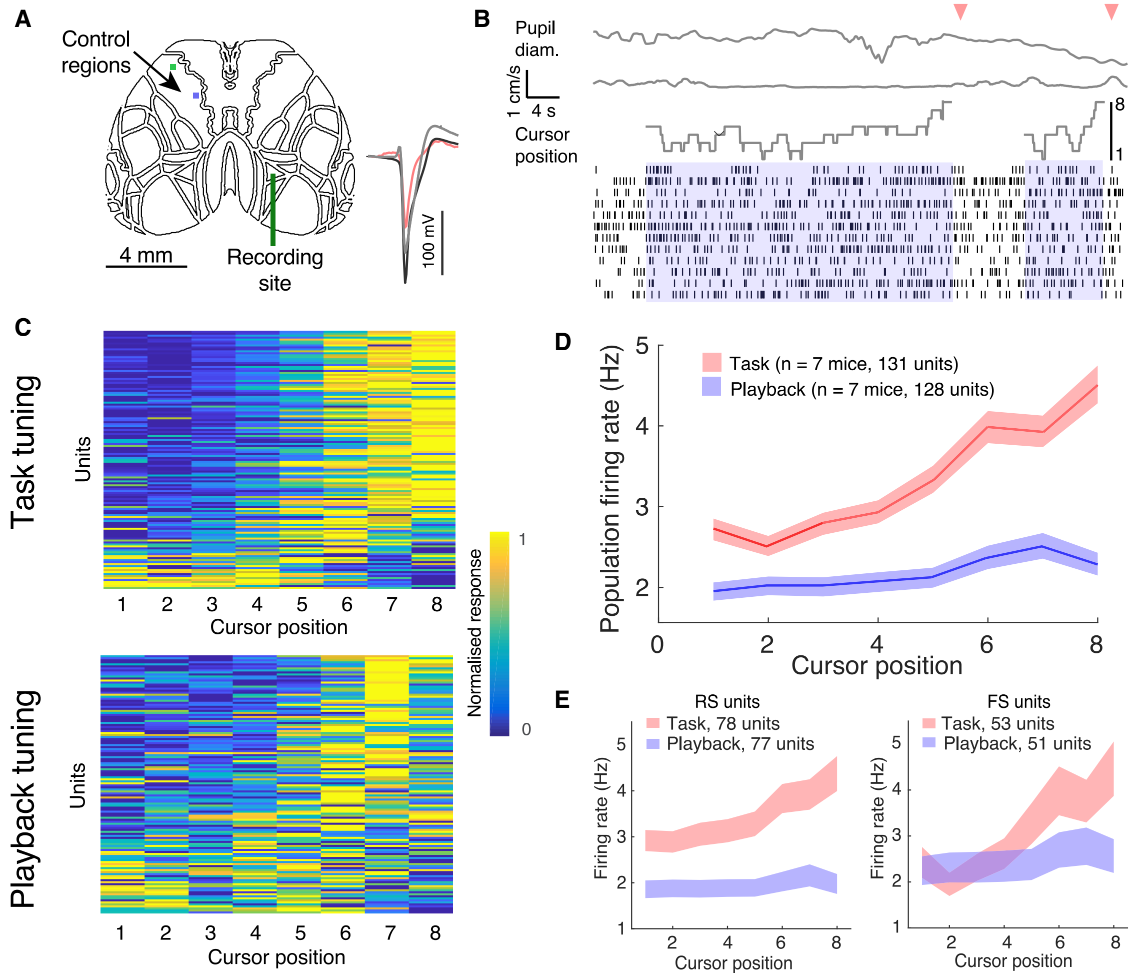

Figure 5. Cursor tuning shifts to target location

(A) Electrophysiological recordings were targeted to AM while animals were performing the imaging-based BMI task, with control regions in the anterior motor cortex. Inset shows example waveforms from 3 isolated units (fast spiking unit in red).

(B) Example spiking during 2 successful trials (trials denoted in blue, hits denoted with pink arrows). At top, traces, from top to bottom, are pupil diameter, running velocity, and visual cursor position.

(C) Normalized tuning to cursor positions for all single units. Each row represents the normalized firing responses to each of 8 cursor positions for every recorded unit in the task (top, N = 131 units) and playback (bottom, N = 128 units) conditions. Firing responses were taken as the average firing rate for a period from 80 to 200 ms from the onset of the cursor presentation.

(D) Average population firing rates for each cursor position during task performance (red) and passive playback (blue). Shaded regions indicate 95% confidence levels.

(E) Left: mean firing rate for regular spiking (RS) units to different cursor positions during task performance (95% confidence interval indicated by shading, n = 7 mice). Right: mean firing rate for fast spiking (FS) units to different cursor positions during task performance (95% confidence interval indicated by shading, n = 7 mice).

position during the task, while firing rates were weakly correlated with pupil diameter during playback (Figures S5G and S5H). The fluorescence activity of the control regions was also uncorrelated with running velocity and pupil diameter (Figures S5I and S5J).

To understand the relationship between the firing rate of individual neurons and dorsal cortex-wide activity, we correlated the spike trains of individual units with fluorescence activity across the brain to build affiliation maps for each unit (Xiao et al., 2017; Clancy et al., 2019; Barson et al., 2020; Peters et al., 2019). We aligned these maps to the common coordinate framework of the Allen Brain Atlas using stereotaxic marks on the skull and sorted these maps by units' preference for different cursor locations. During task performance, the cells most responsive to the target and target-adjacent cursor position were significantly more correlated with activity across the dorsal cortex (Figure S6A). This could mean that the boosting around the target may be the result of a cortex-wide signal, or that units tuned to the target correlate more strongly with the rest of dorsal cortex during task performance, but not playback (Figures S6B and S6C).

CellPress
OPEN ACCESS

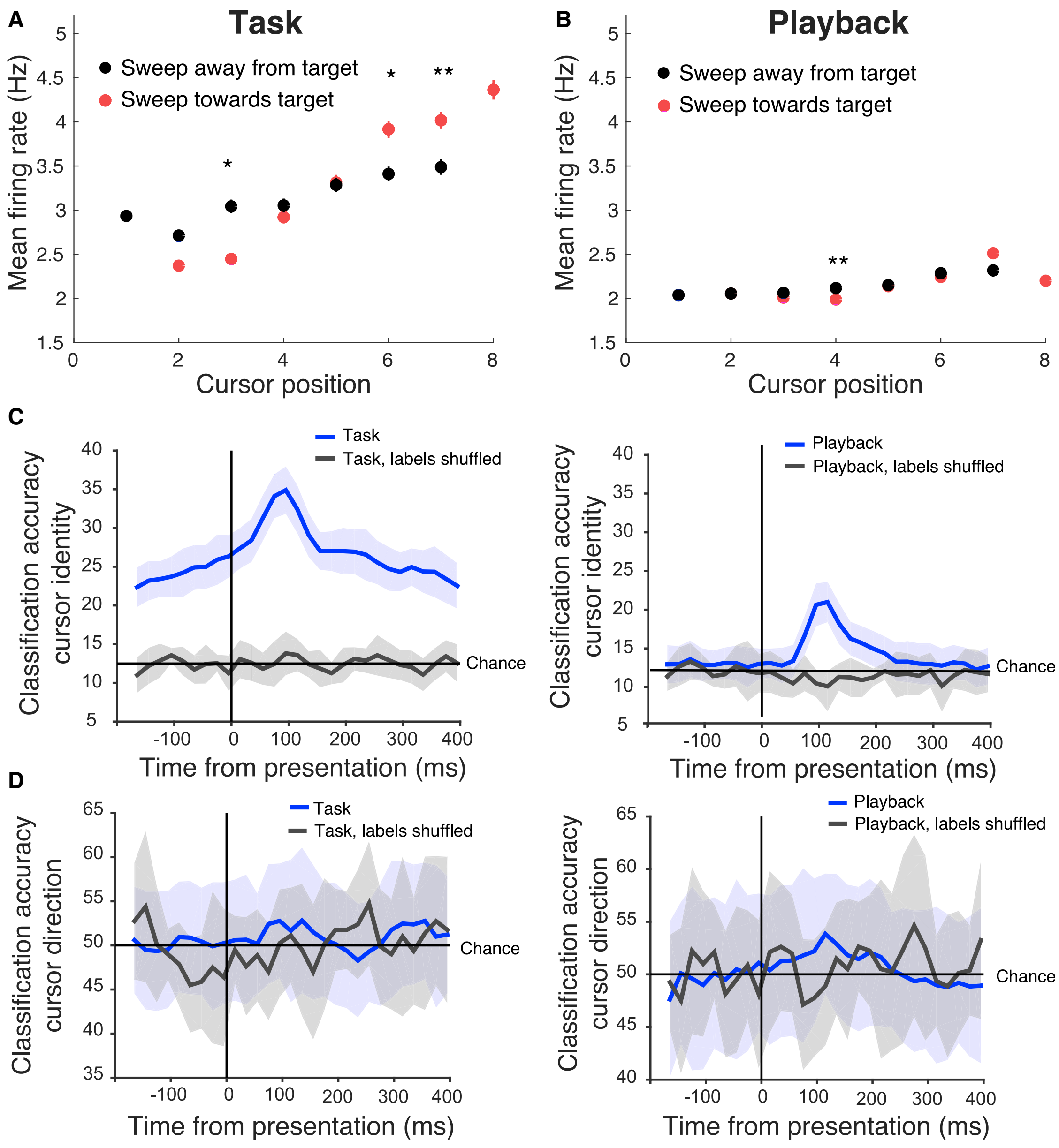

Figure 6. Cursor identity was more decodable from neural activity during task performance

(A) Mean firing rate to different cursor positions depended on whether the preceding cursor position was sweeping toward (red) or away (black) from the direction of the target during task (t test, Bonferroni corrected, *p < 0.05 and **p < 0.01; error bars indicate SEM; n = 131 units).

(B) Cursor sweep direction had little effect on firing rates when animals were passively viewing playback (t test, Bonferroni corrected, **p < 0.01; error bars indicate SEM; n = 128 units.)

(C) Classification accuracy for cursor identity, from population responses trained on real (blue) and shuffled data (gray), for neural responses during task performance (left panel) and passive playback (right panel). Shaded regions denote 95% confidence interval (n = 7 mice). During task performance, the classifier could infer the upcoming cursor position even before presentation (chance level at 12.5%), and rose higher after presentation, suggesting that neural responses encode expectation. This was not true of the passive playback condition, in which case the classifier only performed above chance during the cursor presentation period.

(D) Classification accuracy for cursor direction, from population responses trained on real (blue) and shuffled labels data (gray), for neural responses during task performance (left panel) and passive playback (right panel). Shaded regions denote 95% confidence interval (n = 7 mice). A trained classifier could not perform above chance (50%) in predicting, from firing alone, the direction that the visual cursor was moving, in either the task or playback conditions.

DISCUSSION

The idea of representation is fundamental to the idea of computation, and the neocortex appears to use hierarchies of transformed representations. It has become increasingly clear that cortical sensory representations are not strictly veridical reproductions of the outside world but are shaped by a subject's internal states and goals. We sought to understand how having causal control over an external object affects the cortical sensory representation of that object, given that fluent control must be informed by a constant dialog between action and sensation (Haggard, 2017). Animals learned to causally control an external object using neural calcium signals recorded by widefield imaging and did so by discovering and exploiting experimentally defined mappings between their neural activity and the visual feedback that led to reward. This technique enabled us to identify cortical areas involved in task performance and then to target recordings from individual cells in these areas while animals were engaged in the task. We found that higher visual cortical areas, including area AM, were recruited during expert BMI control, and that single units in AM encoded the same visual cursor differently, depending on whether the animal was causally controlling it or passively viewing it. These results lend evidence to the idea that activity in AM, a potential homolog of the parietal cortex, encodes a subject's intention and self-monitoring of sensorimotor transformations (Andersen and Buneo, 2002; Andersen and Cui, 2009; Desmurget et al., 2009; Aflalo et al., 2015; Cui, 2016).

Previous work indicates that subjects can learn to control neuroprosthetic devices using single cells or bulk electrophysiological signals (Fetz, 1969; Bakay and Kennedy, 1998; Nicolelis, 2001; Serruya et al., 2002; Carmena et al., 2003; Weiskopf et al., 2003; Sitaram et al., 2007; Koralek et al., 2012; Hochberg et al., 2012; Collinger et al., 2013; Clancy et al., 2014; Sadtler et al., 2014; Prsa et al., 2017; Sitaram et al., 2017; Trautmann et al., 2019), but this is the first work, to our knowledge, to apply control using population calcium signals. This technique allowed us to monitor much of the dorsal cortical network as animals learned neuroprosthetic control, whereas previous BMI work has been limited to recording from neighboring neurons. This lends evidence to the idea that manipulating neuroprosthetic devices using aggregate population signals (e.g., from infrared imaging or ultrasound; Shevelev, 1998; Abdelnour and Huppert, 2009), rather than electrophysiological recordings from individual neurons, may afford more stable and minimally invasive control that is robust to losing signals from or damage to individual control cells.

To learn the arbitrary action-outcome relationships required to perform BMI tasks, animals must match internally generated actions or activity with their sensory consequences. To probe how animals learned these contingencies, we changed the regions that controlled the BMI between and within training sessions, meaning that animals could not rely on a habitual activity pattern or strategy, but had to continually explore different neural patterns to achieve reward on different training days. Animals did so by "exploring" with variable neural activity patterns early in a training session, until they discovered a successful activity pattern to exploit (Figure 3). Target hit rates dropped when the reward was dispensed randomly, unlinked to the target zone, indicating that the animals' task performance was goal directed and not habitual.

The spontaneous activity correlations between the control regions were predictive of the animals' fluency of performance, which is instructive for BMI design considerations (Figure 3C; Sadtler et al., 2014; Clancy et al., 2014; Oby et al., 2019). Animals could modestly decorrelate normally correlated brain areas during task execution, although spontaneous activity was widely correlated across the cortex, as seen in prior work (Ledochowitsch et al., 2013). We found this to be true of both anterior and posterior cortical areas—in mice trained to control an auditory cursor, for example, posterior visual control areas could also be decorrelated during the task (Figure 3B). Pupil and locomotion also became decorrelated during task performance, indicating that task engagement and locomotion may engage distinct arousal mechanisms (Figure S5; Vinck et al., 2015; Reimer et al., 2016; Clancy et al., 2019).

By imaging dorsal cortex as animals performed this task, we were able to screen for cortical areas engaged during expert BMI control. On the first day of training, V1 was most active during the task, but as animals learned the task over days, higher visual areas, including the AM, PM, and RL, became more active as animals controlled the cursor. When the same cursors were played back to animals in an open-loop fashion (e.g., not controlled by animals), activation was again mainly evident in V1, suggesting that these higher areas were involved in the goal-directed aspect of task performance. AM and RL are considered by some to be rodent homologs of the parietal cortex (Glickfeld and Olsen, 2017; Wang et al., 2011), which has been shown in humans to be involved in intention and monitoring the mapping between action and outcome (Andersen and Buneo, 2002; Desmurget et al., 2009; Andersen and Cui, 2009; Aflalo et al., 2015; Cui, 2016). However, it was unclear whether the recruitment of these higher visual areas over learning was related to the fact that they are involved in sensorimotor transformations generally or because they are involved more specifically in planning and intentional control.

We targeted extracellular recordings in one of the functionally identified task-active areas, AM, to probe task-related changes in the spiking of single units. We found that units were more active during task performance than passive cursor playback of the animal's recent performance. In particular, units were more responsive to the cursor when it was at the target and target-adjacent positions during the BMI task compared to passive playback, similar to the spatial attention boosting evident in previous work (Moran and Desimone, 1985; Engel et al., 2016), and in accordance with evidence that attention can reshape stimulus representations in a manner that more effectively guides decisions (Ruff and Cohen, 2019). A classifier trained on neural responses to the different cursor positions could effectively classify responses to the visual cursor and could do so with better accuracy when the animal was actively engaged in the task versus passively viewing a playback of the same cursor. In the task condition, the classifier ramped up in accuracy even before the cursor was presented, suggesting that neural responses were prospective, but whether this represents an intention (e.g., a control signal) or an expectation (e.g., a passive

CellPress
OPEN ACCESS

prediction) remains to be determined in future work. This boosting did not reflect reward expectation, and the animal did not use saccades or overt movements to perform the task. This boosting was also sensitive to the task goal: if the cursor was positioned close to the target and sweeping toward it, responses were boosted relative to when it was sweeping away. If the cursor was far from the target and sweeping away from it, then responses were also boosted relative to the cursor at the same position sweeping toward the target. This suggests that firing rates reflect intention and may also reflect a valence of the animal's perceived fluency of cursor control—that is, whether it was successfully guiding the cursor toward its intended goal (Lee and Dan, 2012).

We present a novel task and imaging method for exploring the encoding of action-outcome assessments, which allowed us to simultaneously monitor—with both dorsal cortex-wide and cell-level resolution—what activity patterns support the causal control of external objects. This is a promising proof-of-principle for exploring the potential and limitations of future imaging- and population-activity-based BMIs. Imaging-based BMIs can also serve as a useful paradigm for studying the cortical dynamics involved in flexible learning, affording experimental control of what is learned and by what areas or molecularly defined cell types. This makes it an excellent system to probe how the brain adjusts to sensory and rule manipulations through altering activity patterns within and across areas. Future work could more systematically examine questions including how effectively animals can decorrelate different brain areas depending on underlying anatomical networks. Another promising avenue is credit assignment: BMI affords one less "translational" step in the mapping of neural activity and its sensory consequences than traditional motor learning paradigms. When animals learn a new motor pattern, this is accompanied by changes in neural activity that researchers must first identify (finding where the changes occur and what they are) and then show that this activity is necessary for the new behavior. With BMI learning, researchers can prescribe what neurons are learning what patterns, and also identify exactly when the new pattern emerges and when it is driving the rewarded behavior. For instance, rather than studying how neurons infer credit in limb movements, which result from brain activity across multiple areas, in BMI learning, defined activity patterns of a small group of cortical cells is directly rewarded, reducing the complexity of the problem.

While using wide-field imaging afforded us a view of the dorsal cortex as animals learned neuroprosthetic control, there are a number of limitations to this method. While wide-field fluorescence signals largely reflects neural firing, there is also a contribution from neuropil and hemodynamic effects that were uncorrected in this study, as in our previous work we found it did not significantly affect our findings (Clancy et al., 2019). However, there is clearly some component of the signal contributed by blood vessels (e.g., in Figure 3B), so this is a limitation of the study. Another limitation is that wide-field imaging is limited to recording from the dorsal cortex. Cortical neuroprosthetic control requires interactions with basal ganglia (Koralek et al., 2012; Neely et al., 2018), from which we cannot record using this method. Furthermore, we know from work in humans that the prefrontal cortex (PFC) is involved in the sense of control over external stimuli, but we cannot record signals from the PFC using this preparation due to its obscuration by the frontal sinus.

While we found increased evoked spiking to a visual cursor in the target location using this preparation, we do not know the exact cellular or neuromodulatory mechanisms giving rise to this difference. We found task-related increases in activity emerge over learning specifically in AM, PM, and RL, and are not present during passive visual playback or when visual feedback was randomized. However, as we only recorded neuronal responses in AM, we do not know whether the changes in neural tuning in AM are specific to this area, nor whether they are specifically required for task performance. Future work may address this by using mesoscale 2-photon imaging to record from or manipulate activity in molecularly defined neural subpopulations in parietal and control areas during task performance.

STAR★METHODS

Detailed methods are provided in the online version of this paper and include the following:

- KEY RESOURCES TABLE
- RESOURCE AVAILABILITY
  - Lead contact
  - Materials availability
  - Data and code availability
- EXPERIMENTAL MODEL AND SUBJECT DETAILS
  - Mice
- METHOD DETAILS
  - Surgery
  - Behavioral setup and recordings
  - Behavioral training
  - Task and control conditions
  - Widefield imaging
  - Electrophysiological recordings
  - Data analysis
  - Classifier analysis
- QUANTIFICATION AND STATISTICAL ANALYSIS

SUPPLEMENTAL INFORMATION

Supplemental Information can be found online at https://doi.org/10.1016/j.neuron.2020.12.001.

ACKNOWLEDGMENTS

The authors would like to thank Adrianne Zhong for help with the camera control code; Ivana Orsolic for help with the wide-field imaging setup; Lisa Hoermann for performing surgeries for this project; and Sonja Hofer, Petr Znamenskiy, Spencer Wilson, Alex Naka, and Andrei Khilkevich for manuscript feedback and discussions of this work. This work was supported by the European Research Council (NeuroV1sion 616509 to T.D.M-F.), Swiss National Science Foundation (SNSF 31003A_169802 to T.D.M.-F.), the Wellcome Trust (090843/E/09/Z core grant to the Sainsbury Wellcome Centre) and the Gatsby Charitable Foundation (GAT3361), the EMBO Long-term Fellowship (ALTF 1481-2014 to K.B.C.), the HFSP Postdoctoral Fellowship (LT000414/2015-L to K.B.C.), and the Branco Weiss-Society in Science grant (K.B.C.).

AUTHOR CONTRIBUTIONS

K.B.C. planned and executed the experiments and analyzed the data. K.B.C. and T.D.M.-F. wrote the manuscript.

DECLARATION OF INTERESTS

The authors declare no competing interests.

Received: November 2, 2019
Revised: August 17, 2020
Accepted: December 2, 2020
Published: December 22, 2020

REFERENCES

Abdelnour, A.F., and Huppert, T. (2009). Real-time imaging of human brain function by near-infrared spectroscopy using an adaptive general linear model. Neuroimage *46*, 133–143.

Aflalo, T., Kellis, S., Klaes, C., Lee, B., Shi, Y., Pejsa, K., Shanfield, K., Hayes-Jackson, S., Aisen, M., Heck, C., et al. (2015). Neurophysiology. Decoding motor imagery from the posterior parietal cortex of a tetraplegic human. Science *348*, 906–910.

Andersen, R.A., and Buneo, C.A. (2002). Intentional maps in posterior parietal cortex. Annu. Rev. Neurosci. *25*, 189–220.

Andersen, R.A., and Cui, H. (2009). Intention, action planning, and decision making in parietal-frontal circuits. Neuron *63*, 568–583.

Andersen, R.A., Snyder, L.H., Bradley, D.C., and Xing, J. (1997). Multimodal representation of space in the posterior parietal cortex and its use in planning movements. Annu. Rev. Neurosci. *20*, 303–330.

Athalye, V.R., Ganguly, K., Costa, R.M., and Carmena, J.M. (2017). Emergence of Coordinated Neural Dynamics Underlies Neuroprosthetic Learning and Skillful Control. Neuron *93*, 955–970.e5.

Bagur, S., Averseng, M., Elgueda, D., David, S., Fritz, J., Yin, P., Shamma, S., Boubenec, Y., and Ostojic, S. (2018). Go/No-Go task engagement enhances population representation of target stimuli in primary auditory cortex. Nat. Commun. *9*, 2529.

Bakay, R.A.E., and Kennedy, P.R. (1998). Cognitive Engineering: Proof of Principle. Neurosurgery *43*, 706.

Barson, D., Hamodi, A.S., Shen, X., Lur, G., Constable, R.T., Cardin, J., Crair, M., and Higley, M. (2020). Simultaneous mesoscopic and two-photon imaging of neuronal activity in cortical circuits. Nat. Methods *17*, 107–113.

Barthó, P., Hirase, H., Monconduit, L., Zugaro, M., Harris, K.D., and Buzsáki, G. (2004). Characterization of neocortical principal cells and interneurons by network interactions and extracellular features. J. Neurophysiol. *92*, 600–608.

Carmena, J.M., Lebedev, M.A., Crist, R.E., O'Doherty, J.E., Santucci, D.M., Dimitrov, D.F., Patil, P.G., Henriquez, C.S., and Nicolelis, M.A.L. (2003). Learning to control a brain-machine interface for reaching and grasping by primates. PLOS Biol. *1*, E42.

Chambon, V., Moore, J.W., and Haggard, P. (2015). TMS stimulation over the inferior parietal cortex disrupts prospective sense of agency. Brain Struct. Funct. *220*, 3627–3639.

Churchland, A.K., Kiani, R., and Shadlen, M.N. (2008). Decision-making with multiple alternatives. Nat. Neurosci. *11*, 693–702.

Clancy, K.B., Koralek, A.C., Costa, R.M., Feldman, D.E., and Carmena, J.M. (2014). Volitional modulation of optically recorded calcium signals during neuroprosthetic learning. Nat. Neurosci. *17*, 807–809.

Clancy, K.B., Orsolic, I., and Mrsic-Flogel, T.D. (2019). Locomotion-dependent remapping of distributed cortical networks. Nat. Neurosci. *22*, 778–786.

Collinger, J.L., Wodlinger, B., Downey, J.E., Wang, W., Tyler-Kabara, E.C., Weber, D.J., McMorland, A.J., Velliste, M., Boninger, M.L., and Schwartz, A.B. (2013). High-performance neuroprosthetic control by an individual with tetraplegia. Lancet *381*, 557–564.

Cui, H. (2016). Forward Prediction in the Posterior Parietal Cortex and Dynamic Brain-Machine Interface. Front. Integr. Neurosci. *10*, 35.

Desmurget, M., Reilly, K.T., Richard, N., Szathmari, A., Mottolese, C., and Sirigu, A. (2009). Movement intention after parietal cortex stimulation in humans. Science *324*, 811–813.

Dewey, J. (1896). The reflex arc concept in psychology. Psychol. Rev. *3*, 357–370.

Donoghue, J.P. (2002). Connecting cortex to machines: recent advances in brain interfaces. Nat. Neurosci. *5* (*Suppl*), 1085–1088.

Engel, T.A., Steinmetz, N.A., Gieselmann, M.A., Thiele, A., Moore, T., and Boahen, K. (2016). Selective modulation of cortical state during spatial attention. Science *354*, 1140–1144.

Fetz, E.E. (1969). Operant conditioning of cortical unit activity. Science *163*, 955–958.

Gilissen, S.R.J., Farrow, K., Bonin, V., and Arckens, L. (2020). Reconsidering the Border between the Visual and Posterior Parietal Cortex of Mice. Cereb. Cortex. https://doi.org/10.1101/2020.03.24.005462.

Glickfeld, L.L., and Olsen, S.R. (2017). Higher-Order Areas of the Mouse Visual Cortex. Annu. Rev. Vis. Sci. *3*, 251–273.

Gnadt, J.W., and Andersen, R.A. (1988). Memory related motor planning activity in posterior parietal cortex of macaque. Exp. Brain Res. *70*, 216–220.

Haggard, P. (2017). Sense of agency in the human brain. Nat. Rev. Neurosci. *18*, 196–207.

Harvey, C.D., Coen, P., and Tank, D.W. (2012). Choice-specific sequences in parietal cortex during a virtual-navigation decision task. Nature *484*, 62–68.

Hochberg, L.R., Bacher, D., Jarosiewicz, B., Masse, N.Y., Simeral, J.D., Vogel, J., Haddadin, S., Liu, J., Cash, S.S., van der Smagt, P., and Donoghue, J.P. (2012). Reach and grasp by people with tetraplegia using a neurally controlled robotic arm. Nature *485*, 372–375.

Johnson, P.B., Ferraina, S., Bianchi, L., and Caminiti, R. (1996). Cortical networks for visual reaching: physiological and anatomical organization of frontal and parietal lobe arm regions. Cereb. Cortex *6*, 102–119.

Kahnt, T., Park, S.Q., Haynes, J.-D., and Tobler, P.N. (2014). Disentangling neural representations of value and salience in the human brain. Proc. Natl. Acad. Sci. USA *111*, 5000–5005.

Koralek, A.C., Jin, X., Long, J.D., 2nd, Costa, R.M., and Carmena, J.M. (2012). Corticostriatal plasticity is necessary for learning intentional neuroprosthetic skills. Nature *483*, 331–335.

Lacquaniti, F., Guigon, E., Bianchi, L., Ferraina, S., and Caminiti, R. (1995). Representing spatial information for limb movement: role of area 5 in the monkey. Cereb. Cortex *5*, 391–409.

Ledochowitsch, P., Koralek, A.C., Moses, D., Carmena, J.M., and Maharbiz, M.M. (2013). Sub-mm functional decoupling of electrocortical signals through closed-loop BMI learning. In Proceedings of the 35th Annual International Conference of the IEEE Engineering in Medicine and Biology Society (IEEE), pp. 5622–5625.

Lee, S.-H., and Dan, Y. (2012). Neuromodulation of brain states. Neuron *76*, 209–222.

Licata, A.M., Kaufman, M.T., Raposo, D., Ryan, M.B., Sheppard, J.P., and Churchland, A.K. (2017). Posterior Parietal Cortex Guides Visual Decisions in Rats. J. Neurosci. *37*, 4954–4966.

Lyamzin, D., and Benucci, A. (2019). The mouse posterior parietal cortex: anatomy and functions. Neurosci. Res. *140*, 14–22.

Makino, H., Ren, C., Liu, H., Kim, A.N., Kondapaneni, N., Liu, X., Kuzum, D., and Komiyama, T. (2017). Transformation of Cortex-wide Emergent Properties during Motor Learning. Neuron *94*, 880–890.e8.

Meyers, E. (2013). The neural decoding toolbox. Front. Neuroinform. *7*, 8.

Mohan, H., Gallero-Salas, Y., Carta, S., Sacramento, J., Laurenczy, B., Sumanovski, L.T., de Kock, C.P.J., Helmchen, F., and Sachidhanandam, S. (2018). Sensory representation of an auditory cued tactile stimulus in the posterior parietal cortex of the mouse. Sci. Rep. *8*, 7739.

CellPress
OPEN ACCESS

Mohan, H., de Haan, R., Broersen, R., Pieneman, A.W., Helmchen, F., Staiger, J.F., Mansvelder, H.D., and de Kock, C.P.J. (2019). Functional Architecture and Encoding of Tactile Sensorimotor Behavior in Rat Posterior Parietal Cortex. J. Neurosci. *39*, 7332–7343.

Moran, J., and Desimone, R. (1985). Selective attention gates visual processing in the extrastriate cortex. Science *229*, 782–784.

Mountcastle, V.B., Lynch, J.C., Georgopoulos, A., Sakata, H., and Acuna, C. (1975). Posterior parietal association cortex of the monkey: command functions for operations within extrapersonal space. J. Neurophysiol. *38*, 871–908.

Muir, D.R., and Kampa, B. (2015). FocusStack and StimServer: a new open source MATLAB toolchain for visual stimulation and analysis of two-photon calcium neuronal imaging data. Front. Neuroinform. *8*, 85.

Musall, S., Kaufman, M.T., Juavinett, A.L., Gluf, S., and Churchland, A.K. (2019). Single-trial neural dynamics are dominated by richly varied movements. Nat. Neurosci. *22*, 1677–1686.

Neely, R.M., Koralek, A.C., Athalye, V.R., Costa, R.M., and Carmena, J.M. (2018). Volitional Modulation of Primary Visual Cortex Activity Requires the Basal Ganglia. Neuron *97*, 1356–1368.e4.

Nicolelis, M.A. (2001). Actions from thoughts. Nature *409*, 403–407.

Oby, E.R., Golub, M.D., Hennig, J.A., Degenhart, A.D., Tyler-Kabara, E.C., Yu, B.M., Chase, S.M., and Batista, A.P. (2019). New neural activity patterns emerge with long-term learning. Proc. Natl. Acad. Sci. USA *116*, 15210–15215.

Orsolic, I., Rio, M., Mrsic-Flogel, T.D., and Znamenskiy, P. (2019). Mesoscale cortical dynamics reflect the interaction of sensory evidence and temporal expectation during perceptual decision-making. BioRxiv. https://doi.org/10.1101/552026.

Peters, A.J., Steinmetz, N.A., Harris, K.D., and Carandini, M. (2019). Striatal activity reflects cortical activity patterns. BioRxiv. https://doi.org/10.1101/703710.

Pho, G.N., Goard, M.J., Woodson, J., Crawford, B., and Sur, M. (2018). Task-dependent representations of stimulus and choice in mouse parietal cortex. Nat. Commun. *9*, 2596.

Pisella, L., Gréa, H., Tilikete, C., Vighetto, A., Desmurget, M., Rode, G., Boisson, D., and Rossetti, Y. (2000). An 'automatic pilot' for the hand in human posterior parietal cortex: toward reinterpreting optic ataxia. Nat. Neurosci. *3*, 729–736.

Poort, J., Khan, A.G., Pachitariu, M., Nemri, A., Orsolic, I., Krupic, J., Bauza, M., Sahani, M., Keller, G.B., Mrsic-Flogel, T.D., and Hofer, S.B. (2015). Learning Enhances Sensory and Multiple Non-sensory Representations in Primary Visual Cortex. Neuron *86*, 1478–1490.

Prsa, M., Galiñanes, G.L., and Huber, D. (2017). Rapid Integration of Artificial Sensory Feedback during Operant Conditioning of Motor Cortex Neurons. Neuron *93*, 929–939.e6.

Ratzlaff, E.H., and Grinvald, A. (1991). A tandem-lens epifluorescence macroscope: hundred-fold brightness advantage for wide-field imaging. J. Neurosci. Methods *36*, 127–137.

Reimer, J., McGinley, M.J., Liu, Y., Rodenkirch, C., Wang, Q., McCormick, D.A., and Tolias, A.S. (2016). Pupil fluctuations track rapid changes in adrenergic and cholinergic activity in cortex. Nat. Commun. *7*, 13289.

Rizzolatti, G., Fogassi, L., and Gallese, V. (1997). Parietal cortex: from sight to action. Curr. Opin. Neurobiol. *7*, 562–567.

Ruff, D.A., and Cohen, M.R. (2019). Simultaneous multi-area recordings suggest that attention improves performance by reshaping stimulus representations. Nat. Neurosci. *22*, 1669–1676.

Rushworth, M.F., Ellison, A., and Walsh, V. (2001). Complementary localization and lateralization of orienting and motor attention. Nat. Neurosci. *4*, 656–661.

Sadtler, P.T., Quick, K.M., Golub, M.D., Chase, S.M., Ryu, S.I., Tyler-Kabara, E.C., Yu, B.M., and Batista, A.P. (2014). Neural constraints on learning. Nature *512*, 423–426.

Salkoff, D.B., Zagha, E., McCarthy, E., and McCormick, D.A. (2020). Movement and Performance Explain Widespread Cortical Activity in a Visual Detection Task. Cereb. Cortex *30*, 421–437.

Schmitzer-Torbert, N., Jackson, J., Henze, D., Harris, K., and Redish, A.D. (2005). Quantitative measures of cluster quality for use in extracellular recordings. Neuroscience *131*, 1–11.

Serruya, M.D., Hatsopoulos, N.G., Paninski, L., Fellows, M.R., and Donoghue, J.P. (2002). Instant neural control of a movement signal. Nature *416*, 141–142.

Shevelev, I.A. (1998). Functional imaging of the brain by infrared radiation (thermoencephaloscopy). Prog. Neurobiol. *56*, 269–305.

Siegle, J.H., López, A.C., Patel, Y.A., Abramov, K., Ohayon, S., and Voigts, J. (2017). Open Ephys: an open-source, plugin-based platform for multichannel electrophysiology. J. Neural Eng. *14*, 045003.

Sitaram, R., Caria, A., Veit, R., Gaber, T., Rota, G., Kuebler, A., and Birbaumer, N. (2007). FMRI brain-computer interface: a tool for neuroscientific research and treatment. Comput. Intell. Neurosci. *2007*, 25487.

Sitaram, R., Ros, T., Stoeckel, L., Haller, S., Scharnowski, F., Lewis-Peacock, J., Weiskopf, N., Blefari, M.L., Rana, M., Oblak, E., et al. (2017). Closed-loop brain training: the science of neurofeedback. Nat. Rev. Neurosci. *18*, 86–100.

Stringer, C., Pachitariu, M., Steinmetz, N., Reddy, C.B., Carandini, M., and Harris, K.D. (2019). Spontaneous behaviors drive multidimensional, brainwide activity. Science *364*, 255.

Sugrue, L.P., Corrado, G.S., and Newsome, W.T. (2004). Matching behavior and the representation of value in the parietal cortex. Science *304*, 1782–1787.

Tervo, D.G.R., Proskurin, M., Manakov, M., Kabra, M., Vollmer, A., Branson, K., and Karpova, A.Y. (2014). Behavioral variability through stochastic choice and its gating by anterior cingulate cortex. Cell *159*, 21–32.

Trautmann, E.M., O'Shea, D.J., Sun, X., Marshel, J.H., Crow, A., Hsueh, B., Vesuna, S., Cofer, L., Bohner, G., Allen, W., et al. (2019). Dendritic calcium signals in rhesus macaque motor cortex drive an optical brain-computer interface. BioRxiv. https://doi.org/10.1101/780486.

Vanni, M.P., and Murphy, T.H. (2014). Mesoscale transcranial spontaneous activity mapping in GCaMP3 transgenic mice reveals extensive reciprocal connections between areas of somatomotor cortex. J. Neurosci. *34*, 15931–15946.

Vinck, M., Batista-Brito, R., Knoblich, U., and Cardin, J.A. (2015). Arousal and locomotion make distinct contributions to cortical activity patterns and visual encoding. Neuron *86*, 740–754.

Wang, Q., Gao, E., and Burkhalter, A. (2011). Gateways of ventral and dorsal streams in mouse visual cortex. J. Neurosci. *31*, 1905–1918.

Weiskopf, N., Veit, R., Erb, M., Mathiak, K., Grodd, W., Goebel, R., and Birbaumer, N. (2003). Physiological self-regulation of regional brain activity using real-time functional magnetic resonance imaging (fMRI): methodology and exemplary data. Neuroimage *19*, 577–586.

Wekselblatt, J.B., Flister, E.D., Piscopo, D.M., and Niell, C.M. (2016). Large-scale imaging of cortical dynamics during sensory perception and behavior. J. Neurophysiol. *115*, 2852–2866.

Wisniewski, D., Reverberi, C., Momennejad, I., Kahnt, T., and Haynes, J.-D. (2015). The Role of the Parietal Cortex in the Representation of Task-Reward Associations. J. Neurosci. *35*, 12355–12365.

Xiao, D., Vanni, M.P., Mitelut, C.C., Chan, A.W., LeDue, J.M., Xie, Y., Chen, A.C., Swindale, N.V., and Murphy, T.H. (2017). Mapping cortical mesoscopic networks of single spiking cortical or sub-cortical neurons. eLife *6*, e19976.

Zacksenhouse, M., Lebedev, M.A., Carmena, J.M., O'Doherty, J.E., Henriquez, C., and Nicolelis, M.A.L. (2007). Cortical modulations increase in early sessions with brain-machine interface. PLOS ONE *2*, e619.

Zapparoli, L., Seghezzi, S., Zirone, E., Guidali, G., Tettamanti, M., Banfi, G., Bolognini, N., and Paulesu, E. (2020). How the effects of actions become our own. Sci. Adv. *6*, eaay8301.

STAR★METHODS

KEY RESOURCES TABLE

REAGENT or RESOURCE	SOURCE	IDENTIFIER
Experimental Models: Organisms/Strains		
Mouse: TRE-Gcamp6s	Jackson labs	JAX 024742; RRID: IMSR_JAX:024742
Mouse: B6.CBA-Tg(Camk2a-tTA)1Mmay/DboJ mice	Jackson labs	JAX 007004; RRID: IMSR_JAX:007004
Software and Algorithms		
Allen Brain Atlas API	Allen Institute	http://help.brain-map.org/display/api/Allen+Brain+Atlas+API
MATLAB 2015	MATLAB	https://uk.mathworks.com/help/matlab/release-notes-R2015b.html
Labview 2012	National Instruments	https://www.ni.com/en-gb/support/downloads/software-products.html
OpenEphys	Siegle et al., 2017	https://open-ephys.org/store
Klustakwik	Schmitzer-Torbert et al., 2005	https://github.com/klusta-team/klustakwik
Mapped Tensor (MATLAB)	Muir and Kampa, 2015	https://uk.mathworks.com/matlabcentral/fileexchange/29694-better-memory-mapped-files-in-matlab

RESOURCE AVAILABILITY

Lead contact

Further information and requests for resources and reagents should be directed to Kelly Clancy, the lead contact (k.clancy@ucl.ac.uk).

Materials availability

This study did not generate new unique reagents or mouse lines.

Data and code availability

The data and code used in this study are available from the lead contact upon reasonable request.

EXPERIMENTAL MODEL AND SUBJECT DETAILS

Mice

All experimental procedures were carried out in accordance with institutional animal welfare guidelines and licensed by the Swiss cantonal veterinary office. TRE-Gcamp6s mice (Wekselblatt et al., 2016) (Jackson Laboratories, RRID: IMSR_JAX:024742) were crossed with B6.CBA-Tg(Camk2a-tTA)1Mmay/DboJ mice (Jackson Laboratories, RRID: IMSR_JAX:007004), to drive the expression of gCamp6s in CamKII+ pyramidal neurons. Animals were housed in a facility using a reversed light cycle, and recordings were taken during their active period. Eleven female mice were trained on the task, and we took electrophysiological recordings from seven of these, ranging between P55-P75. All animals were healthy, had never undergone previous procedures, and ranged in weight from 15-19 g. Sample sizes were not statistically determined, but were consistent with previous papers using related methodology (Clancy et al., 2019; Xiao et al., 2017). Animals were group housed with same-sex littermates in enriched environments (including running wheels, cardboard tubes and chewing toys).

METHOD DETAILS

Surgery

A week before training, mice were prepared for imaging. Animals were anaesthetised with a mixture of fentanyl (0.05 mg per kg), midazolam (5.0 mg per kg), and medetomidine (0.5 mg per kg). The animal's scalp was resected and a head plate was secured to the skull. Four stereotaxically placed marks were made to enable alignment of the imaged brain with the Allen Brain Atlas (http://mouse.brain-map.org/static/atlas) post hoc, using the Allen Brain API (http://help.brain-map.org/display/api/Allen+Brain+Atlas+API). The exposed skull was cleaned and covered with transparent dental cement to avoid infection, and to cover the cut scalp edges (C&B Metabond). This was polished to enhance the transparency of the preparation. A custom-made 3D printed light shield was cemented to the skull and head plate to avoid light leaks from the visual feedback presented on two computer monitors.

Behavioral setup and recordings

The recording chamber was sound-isolated and shielded from outside light. Mice were head-fixed under the microscope and free to run on a Styrofoam running wheel (diameter = 20 cm, width = 12 cm). The animals' movements were recorded using a rotary encoder in the wheel axis (pulse rate 1000, Kubler). Two monitors were placed side by side in front of the mouse, angled toward one another (21.5'' monitors, ~20 cm from mouse, covering ~100x70 degrees of visual space), similarly to the setup described in Poort et al. (2015). A reward port was place in front of the animal, where reward delivery was triggered via pinch solenoid one second after target hit (NResearch) and animal licks were detected using a custom piezo element coupled to the spout. All behavioral data were recorded using custom MATLAB software and a PCI-6320 acquisition board (National Instruments).

On electrophysiological recording days, pupil recordings were taken by illuminating the animal's right eye with a custom IR-light source and imaging with a CMOS camera (DMK22BUC03, Imaging Source, 30 Hz) using custom MATLAB software. Pupil size was determined as described in Orsolic et al. (2019): images were first smoothed with a 2-D Gaussian filter and thresholded to low luminance areas. These thresholded regions were then filtered by circularity and size to automatically detect the pupil region. Pupil edges were detected using the canny method, and ellipses were iteratively fit to the region, tasked to minimize the geometric distance between the area outline and the fit ellipse using nonlinear least-squares (MATLAB function fitellipse, Richard Brown). The pupil diameter was taken to be the major axis of the ellipse, then normalized by animal. Pupil recordings from one animal had to be discarded, as the video was not sufficiently in focus.

Behavioral training

After recovery, mice were acclimatised to head fixation for a minimum of two days, and started on food restriction. Awake animals were head-fixed under the microscope and free to run on a Styrofoam wheel. A baseline of spontaneous activity was taken on every training day (10-20 minutes) in order to estimate spontaneous hit rates. The decoder was calibrated such that animals achieved ~25% performance on their first day. Two small control regions were chosen for real-time read out. In the case of visual feedback task, these were all located in primary and secondary motor cortex, avoiding ALM. In the auditory feedback task, control regions were placed in posterior cortex, over visual and retrosplenial areas. The placement of the two control regions was usually ipsilateral but sometimes contralateral to each other. The same control regions were used for the first few days of training, then changed from day to day, or within sessions, so that animals did not learn a fixed control strategy (see Table S1).

Activity was imaged at 40 Hz and the mean fluorescence from each control region was transmuted to the cursor's position on screen with a simple transform:

$$p(t) = A_1 F_{R1} - A_2 F_{R2} + B \quad \text{(Equation 1)}$$

where p is the cursor position at time t, F_{R1} and F_{R2} are the instantaneous fluorescence ($\Delta F/F$) of control regions one and two, respectively, and A_1, A_2 and B are coefficients set based on the daily spontaneous baseline recordings (minimum 10 minutes). P was rounded to the nearest integer to determine the discrete cursor location. A1 and A2 were determined by dividing the full dynamic range of each recorded area during the baseline by half the number of cursor positions:

$$A_1 = [max(F_{R1}) - min(F_{R1})]/4; = [max(F_{R2}) - min(F_{R2})]/4; \quad \text{(Equation 2)}$$

B represents the activity baseline of both areas. The chance performance was then assessed by running the baseline data through the decoder to estimate how often the animal would have achieved the target with spontaneous activity.

$\Delta F/F$ was calculated using a moving baseline, set as the tenth percentile of points from the preceding 20 s of data. The raw fluorescence was converted to $\Delta F/F$ using a moving baseline of 5 minutes of activity. The display updated at approximated 10 Hz, with a latency of 300 ms from camera to screen, measured using a photodiode placed on one of the monitors (Thorlabs, PDA100A-EC). Activity in R1 would cause the cursor to move toward the target location in the center of the animal's visual field, while increased activity in R2 would cause the cursor to move away from the target zone. The cursor was presented on two monitors so that the animal could track the cursor with both eyes; its goal was to move the cursors presented on the two screens on either side to the middle of its visual field. These changes were binned, such that the cursor could take one of eight possible locations on the screen. The cursor had to be held at the target position for 0.3 s to count as a hit, at which point the cursor disappeared. When a target was hit, a MATLAB-controlled Data Acquisition board (National Instruments, Austin, TX) triggered the administration of a soyamilk reward

following a 1 s delay. The next trial could be initiated within 5 s of reward delivery, but only when the activation of R1 relative to R2 returned to the mean value recording during spontaneous activity (to ensure enough time had passed for large transients to decay, given slow calcium dynamics). This was return to baseline condition was rarely triggered (~5% of trials) and on average lasted under 2 s. If the animal did not bring the cursor to the target within a 30 s trial, the cursor disappeared, and the animal received a white noise tone and a 10 s 'time out.'

We trained a separate cohort of four mice using an auditory, rather than visual, feedback cursor, where activity was transmuted to the pitch of a feedback tone (Clancy et al., 2014). As with the visual feedback task, a spontaneous baseline was recorded every day (10-20 minutes) to assess chance levels of performance and calibrate the decoder. Activity from two arbitrarily chosen regions was entered into an online transform algorithm that related neural activity to the pitch of an auditory cursor:

$$f(t) = A_1 e^{F_{R1} 1} - A_2 e^{RF_{R2}} + B \quad \text{(Equation 3)}$$

Where f is the cursor frequency, F_{R1} is the instantaneous ΔF/F of R1, F_{R2} the instantaneous ΔF/F of R2, and A1, A2, and B are coefficients set based on the daily baseline recording. As above, ΔF/F was calculated using a moving baseline, set as the tenth percentile of points from the preceding 20 s of data. Linear changes in firing rate resulted in exponential changes in cursor frequency, and frequency changes were binned in quarter-octave intervals to match rodent psychophysical discrimination thresholds. As with the visual task, a trial was marked incorrect if the target pitch was not achieved within 30 s of trial initiation. The auditory feedback was played using speakers mounted on 2 sides of the imaging platform.

Task and control conditions

In the task condition, the position of the presented cursor was determined by the control regions' instantaneous activity, according to Equation 1 above. Rewards were given when the cursor hit the target zone (cursor position 8). Similarly, in the random reward condition, intended to test whether animal's task engagement was goal-directed or habitual, the position of the presented cursor was determined by the control regions' instantaneous activity, according to Equation 1 above. However, here rewards were not linked to the cursor position, but given out at random time intervals at a rate matched to an expertly performing animal (approximately 1.5 rewards/minute). In the random feedback condition, intended to confirm that the animal was using the visual feedback to inform its behavior, the position of the presented cursor was not linked to the control regions' instantaneous activity, but instead was drawn randomly from a Gaussian distribution matching the mean and variance of a typical task condition. The animal could still receive rewards if they achieved the correct neural activity pattern, but their performance drop suggests they could not achieve that in the absence of appropriately linked sensory feedback. In the passive playback condition, the presented cursor position was no longer linked to the control regions' activity, but was purely a replay of the cursor positions from a training session the animal had undergone previously. Thus, the cursor positions and timing of trials in the playback condition were matched to those of the task condition. Because the statistics of the sensory presentations between the task and playback conditions (e.g., cursor position identity, and likelihood of transition between different positions) were identical, this allowed a cleaner comparison of neural responses in these conditions.

Widefield imaging

Widefield imaging was performed through the intact skull using a custom-built epifluorescence macroscope with photographic lenses in a face-to-face configuration (85mm f/1.8D objective, 50mm f/1.4D tube lens; Ratzlaff and Grinvald, 1991). Data were recorded using a CMOS camera (Pco.edge 5.5, PCO, Germany) in global shutter mode. 16-bit images were acquired at a rate of 40 Hz and binned 2x2 online using custom-made LABVIEW software. A constant illumination at 470 nm was provided (M470L3, Thorlands, excitation filter FF02-447/60-25), with average power ~0.05 mW/mm2 (emission filter 525/50-25, Semrock). The imaging site was shielded from light contamination using a 3D-printed blackout barrier glued to the animal's skull. Signals from the two control regions were sent via UDP to a computer providing visual or auditory feedback to the mouse, using custom MATLAB software.

Electrophysiological recordings

The day before recording, mice were anesthetised with isofluorane and a small craniotomy was opened over AM, which was functionally identified during task performance, and stereotaxically confirmed. The craniotomy was kept damp with Ringer's solution and sealed with KwikSil (World Precision Instruments). Recordings were taken on the following day to avoid residual effects of anesthesia.

On the recording day, animals were head-fixed under a custom-built widefield microscope, the skull and cortex were cleaned with Ringer's solution, and the KwikSil plug removed from the craniotomy. A custom-designed silicon probe (64 channels, 2 shanks, Neuronexus, as described in Clancy et al., 2019) was inserted at an angle of ~45 degrees from normal to cortex. The probe consisted of two shanks with 64 sites total, organized into 16 'tetrodes', each consisting of 4 sites located 25 um apart from each other within-tetrode, and tetrodes spaced 130 um apart from each other. A small amount of KwikSil or agar was used to cover the exposed cortex after the probe was in place. After allowing the probe to settle for 20-30 minutes, neural activity was recorded using the OpenEphys recording system (Siegle et al., 2017). Behavioral and stimulation data, including pulses representing each camera frame, were recorded using OpenEphys, enabling the alignment of electrophysiological signals with imaging and behavioral data. Ephys recordings were filtered between 700 and 7000 Hz, and spikes detected using the Klustakwik suite (Schmitzer-Torbert et al., 2005). Clusters

were assigned to individual units by manual inspection, excluding any units without a clear refractory period. Units were separated into fast and broad spiking units by their peak-to-trough time, using a cutoff of 0.66 ms (Barthó et al., 2004).

Data analysis

Raw imaging data were checked for dropped frames, spatially binned 2x2, and loaded into MATLAB as a mapped tensor (Muir and Kampa, 2015). The raw fluorescence was converted to ΔF/F using a moving baseline, calculated as the tenth percentile of points from the preceding 20 s of data. We did not perform hemodynamic correction as previous work indicates that hemodynamic and flavoprotein signals contribute minimally compared to the calcium responses (Vanni and Murphy, 2014; Xiao et al., 2017; Clancy et al., 2019).

Task-activation maps were calculated by taking the normalized average of fluorescence movies during the task, or visual cursor playback period, subtracted by periods when animals were not performing the task or viewing any visual stimuli (including periods of spontaneous activity, and reward collection). To ensure that differences between early and late in training were not influenced by possible differences in the statistics of the visual feedback cursor, we randomly excluded success trials on late training days in order to have comparable numbers of success and failure trials between early and late training, however, including or excluding these trials did not influence the result. To build the single-unit affiliation maps (Figure S6; see also Clancy et al., 2019), spike trains were binned to match imaging frames, and maps were calculated by taking the correlation of each unit's spike train with each pixel's ΔF/F.

Spectral entropy was calculated in 10 s windows, each overlapping by 5 s. The calcium signal of the control areas was transformed into power spectral density (PSD) during these windows (the magnitude squared of the signal's Fourier transform). This was then used to calculate the spectral entropy for that time span:

$$SE = -\sum_{f=0}^{f=\frac{f_s}{2}} PSD_n(f)log_2[PSD_n(f)]$$

Where SE is the spectral entropy, PSD_n is the normalized PSD, and f_s is the sampling frequency.

Classifier analysis

Spike data were binned into 50 ms bins, and split into 40 segments for training: 39 of these splits were used for training the classifier and 1 was used for testing. The data were z-score normalized so that high firing rate units didn't bias the classifier results. This data was then used, along with the cursor position labels, to train the classifier: a mean vector was created for each cursor position class based on the training data, and predictions were made on the test data by choosing the label class with the maximum correlation between the test and training mean vectors. These predicted labels were compared with the true labels to generate an average classifier accuracy over each tested time bin. This process was repeated 20 times using different training/test splits to cross-validate the results. The final reported classification accuracy is the mean of these 20 runs.

QUANTIFICATION AND STATISTICAL ANALYSIS

All data were analyzed using custom code in MATLAB. The statistical tests used in our analyses are indicated in the figure legends, which also includes the value of n, and whether it refers to animals or single units. Differences were tested using Student's t test, and Bonferroni corrected where apropriate. P values are reported in the figures as well as legends, and significance is herein defined as p less than 0.05. The pupil video for one animal, taken on the final day of recording, had to be excluded as it was too out of focus to determine the pupil size. Sample sizes were not statistically determined, but were consistent with previous papers using related methodology (Clancy et al., 2019; Xiao et al., 2017). Data were assumed to be normal but this was not systematically tested. Animals were trained in three separate cohorts (the first, a cohort of four animals trained on the auditory feedback task, the second, a cohort of 5 animals trained on the visual feedback task, and the third, a cohort of 2 animals trained on the visual feedback test) as an internal replication.

Neuron, Volume *109*

Supplemental Information

The sensory representation of causally controlled objects

Kelly B. Clancy and Thomas D. Mrsic-Flogel

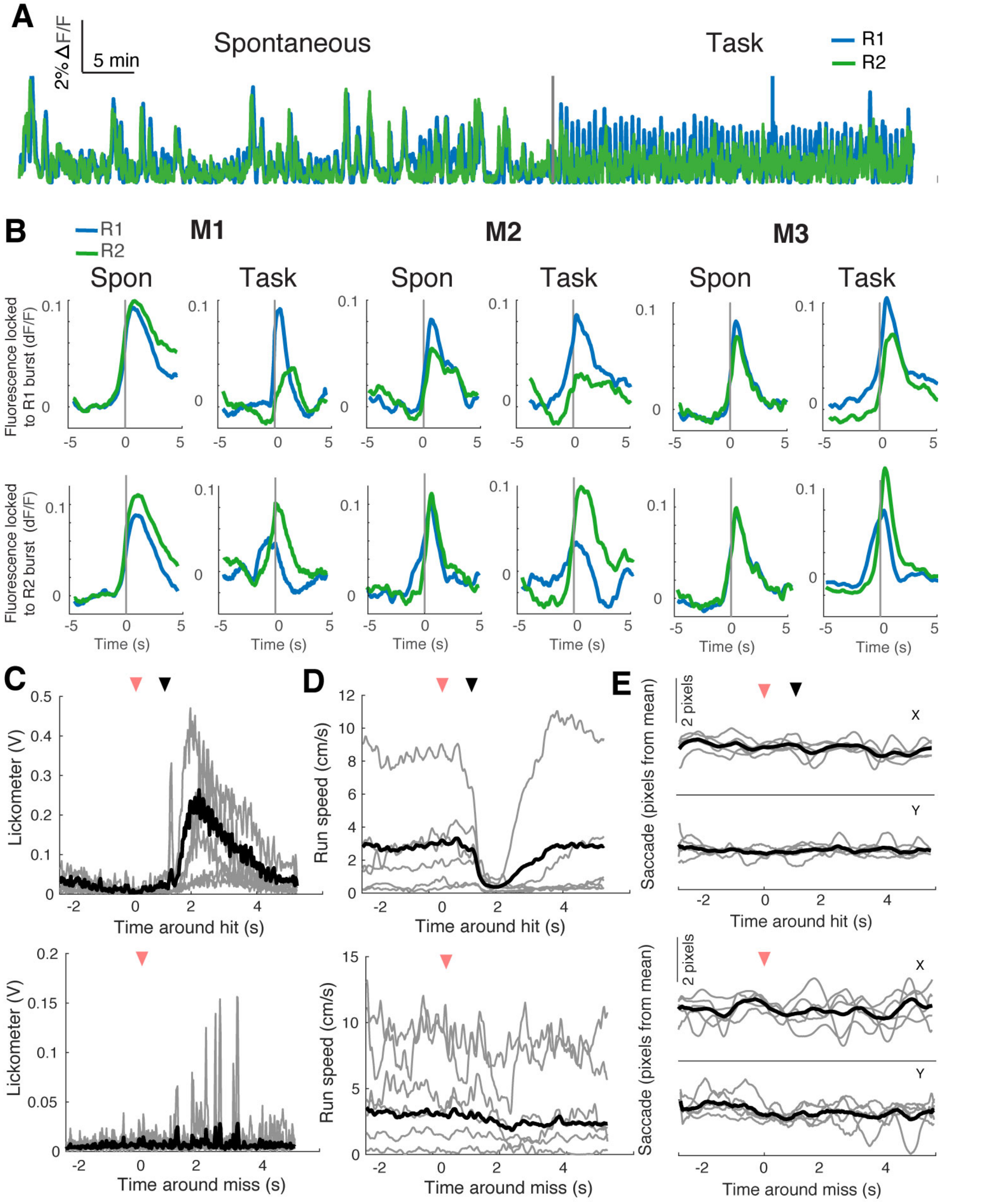

Supplemental Figure 1. Animals did not use gross motor movements to perform task (related to Figure 1)

A. Example fluorescence trace from R1 and R2 in pre-task spontaneous activity, and during task, indicating a clear change from spontaneous patterns. **B.** Analysis of activity throughout the task indicated that activity in the two control regions generally became decoupled during task performance, and not just around hits. Average ΔF/F in the control regions triggered by bursts in R1 (top row) or R2 (bottom row), for the same three example animals, for spontaneous activity (left) and during the training session (right). The burst threshold was taken as the z-scored value of ΔF/F > 3, in order to capture

large events. **C.** Lick averaged around hits (top panel) and misses (bottom panel) on a day in late training (n = 7 mice, grey traces indicate individual mice, black trace indicates mean). Pink arrow indicates target hit (top panel) or time out (bottom panel), black arrow indicates reward delivery (top panel). **D.** Animal's running speed, averaged around hits (top panel) and misses (bottom panel) on a day in late training (n = 7 mice, grey traces indicate individual mice, black trace indicates mean). Pink arrow indicates target hit (top panel) or time out (bottom panel), black arrow indicates reward delivery (top panel). **E.** Eye saccades averaged around hits (top panel) and misses (bottom panel) on final day of training (n = 6 mice). Average movement is shown for both x and y directions of pupil image. Pink arrow indicates target hit (top panel) or time out (bottom panel), black arrow indicates reward delivery (top panel).

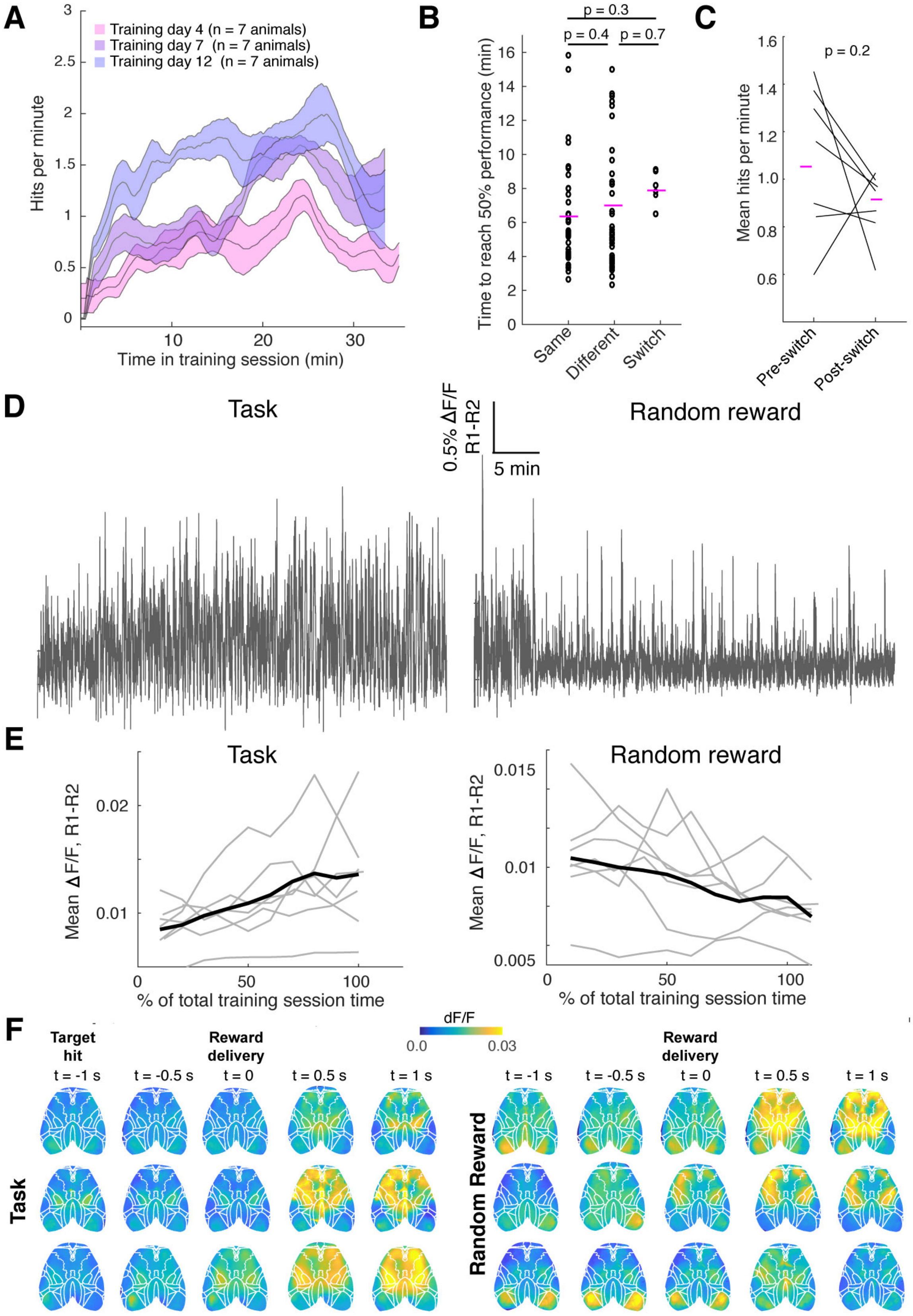

Supplemental Figure 2: Animals exhibit quasi-intentional, specific neural activity control during task performance (related to Figure 2)

A. Hit rate over the course of a training session averaged over 7 mice for three training days (shaded area indicates s.e.m., n = 7 animals for each day). **B.** Once animals were performing above chance (from day 4 on), there was no significant difference in the time it took animals to reach 50% performance whether they were using the same ROIs as the previous day of training, or different ones (t-test, $p = 0.4$). On day 6, when ROIs were switched mid-session, there was also no significant difference in the time it took, post-switch, for the animal to recover their performance to 50% compared to animals using the same or different control regions to the day before (t-test, $p=0.3$, 0.7 respectively, each dot indicates performance on one training day). **C.** On the day control regions were switched mid-session, there was no significant difference in performance between animals before their control regions were switched, and their recovery after the switch. Each line represents one mouse (n = 7 mice). **D.** Example trace of the difference between control areas' activity during task (R1-R2) for one mouse, indicating an increase in differential modulation over the course of a training session (left). These modulations decreased when reward was provided randomly (right). **E.** The slope of the difference in the two control regions fluorescence over training was positive during the normal task condition (left, $p < 0.01$, light-weighted lines each represent one of n = 7 mice, heavy line represents mean) and negative over the course of the randomised reward condition (right, $p < 0.01$, light-weighted lines each represent one of n = 7 mice, heavy line represents mean). **F.** (Left) Example activity maps locked around reward delivery for three animals performing the task. One second before the reward delivery marks the target hit, after which the cursor disappeared during the 1 second wait time. After reward delivery, strong activity is evident in frontal areas when animals are licking and touching the spout. (Right) Example activity maps locked around reward delivery for three animals in the random reward condition. Rewards were given randomly, unlinked to target hits, so the visual cursor was usually present throughout the reward collection period. After reward delivery, strong activity is evident in frontal areas.

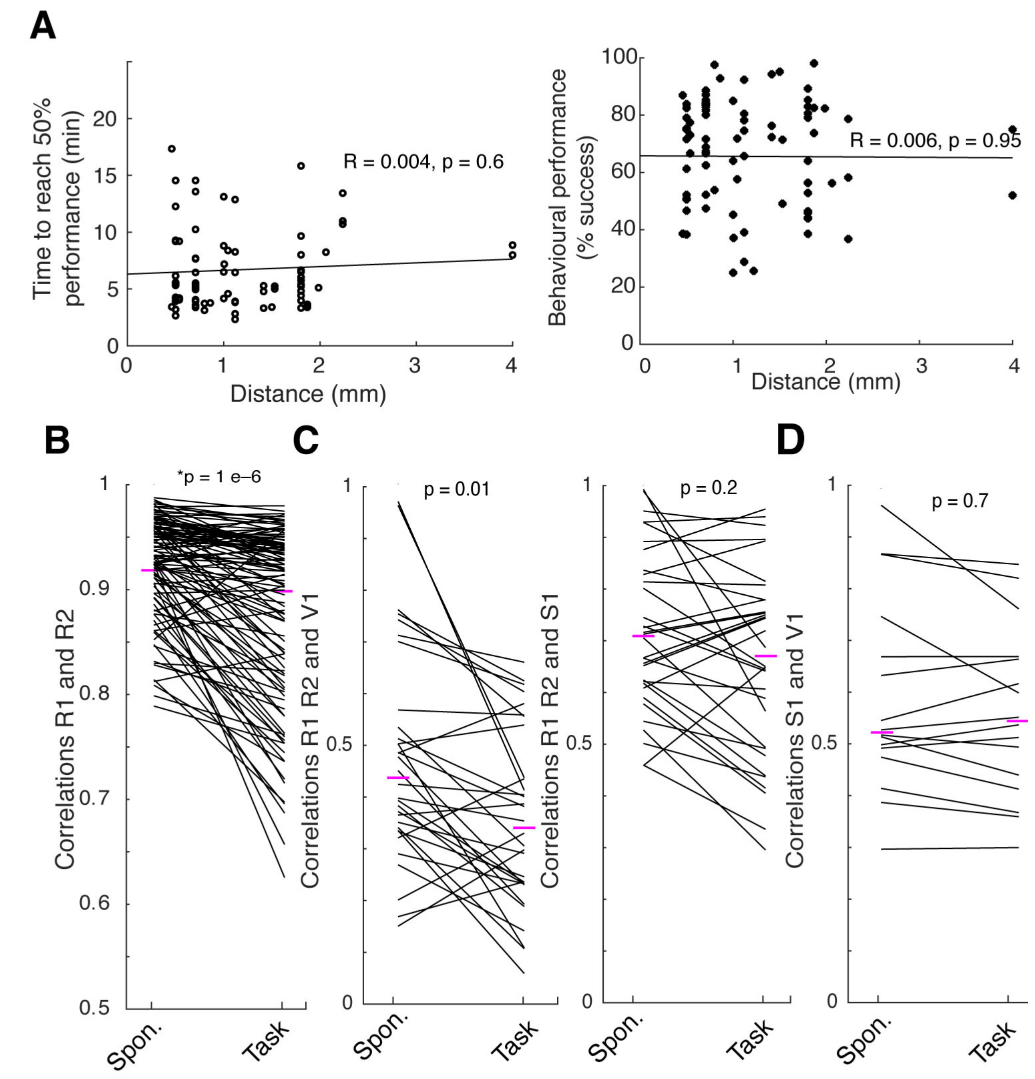

Supplemental Figure 3. Animals could modestly decorrelate areal brain activity during task performance (related to Figure 3)

A. (Left panel) The distance between control regions did not have an effect on the time it took animals to achieve 50% performance (for 83 training sessions, including all days of above chance performance from all 7 animals, e.g. from day 4 on). (Right panel) The distance between control regions did not have an effect on final performance (for 83 training sessions, including all days of above chance performance from all 7 animals, e.g. from day 4 on). **B.** Correlations between R1 and R2 dropped modestly from spontaneous levels during task performance (104 control region pairs from 7 animals, on all training days: on average 15 days of training per mouse, paired t-test, p = 1e-6). **C.** Left panel: correlations between control regions and activity in V1 dropped between spontaneous activity and task performance, but correlations between control regions and primary somatosensory cortex (S1) were not significantly different between conditions. Areas were identified by registering imaged brain surface with Allen Brain Atlas by stereotaxic marks; 7 animals, for expert level performance on days 9 and 14 of training paired t-test, p = 0.01, p=0.1, respectively for V1, S1). **D.** Correlations between primary visual cortex and primary somatosensory cortex did not change between spontaneous

activity and task performance (areas as identified by registering imaged brain surface with Allen Brain Atlas by stereotaxic marks; 7 animals, for expert level performance on days 9 and 14 of training paired t-test, $p = 0.02$).

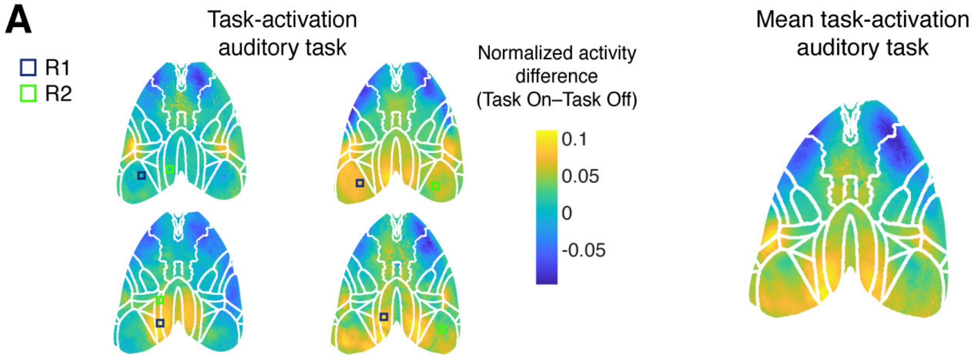

Supplemental Figure 4. Expert animals had increased activity in higher visual areas during task performance (related to Figure 4)

A. Left panel: example task-related activity maps from four animals trained on an auditory version of the task. Right panel: mean task-related activity map indicating involvement of RL, a lateral homolog of parietal cortex in mice and a multimodal associative area (mean from n = 4 mice). Visual areas appear task-activated (despite there being no visual feedback in this task) likely due to the fact that the control regions were placed over visual areas. Control regions are shown as slightly larger than they actually were for better visibility.

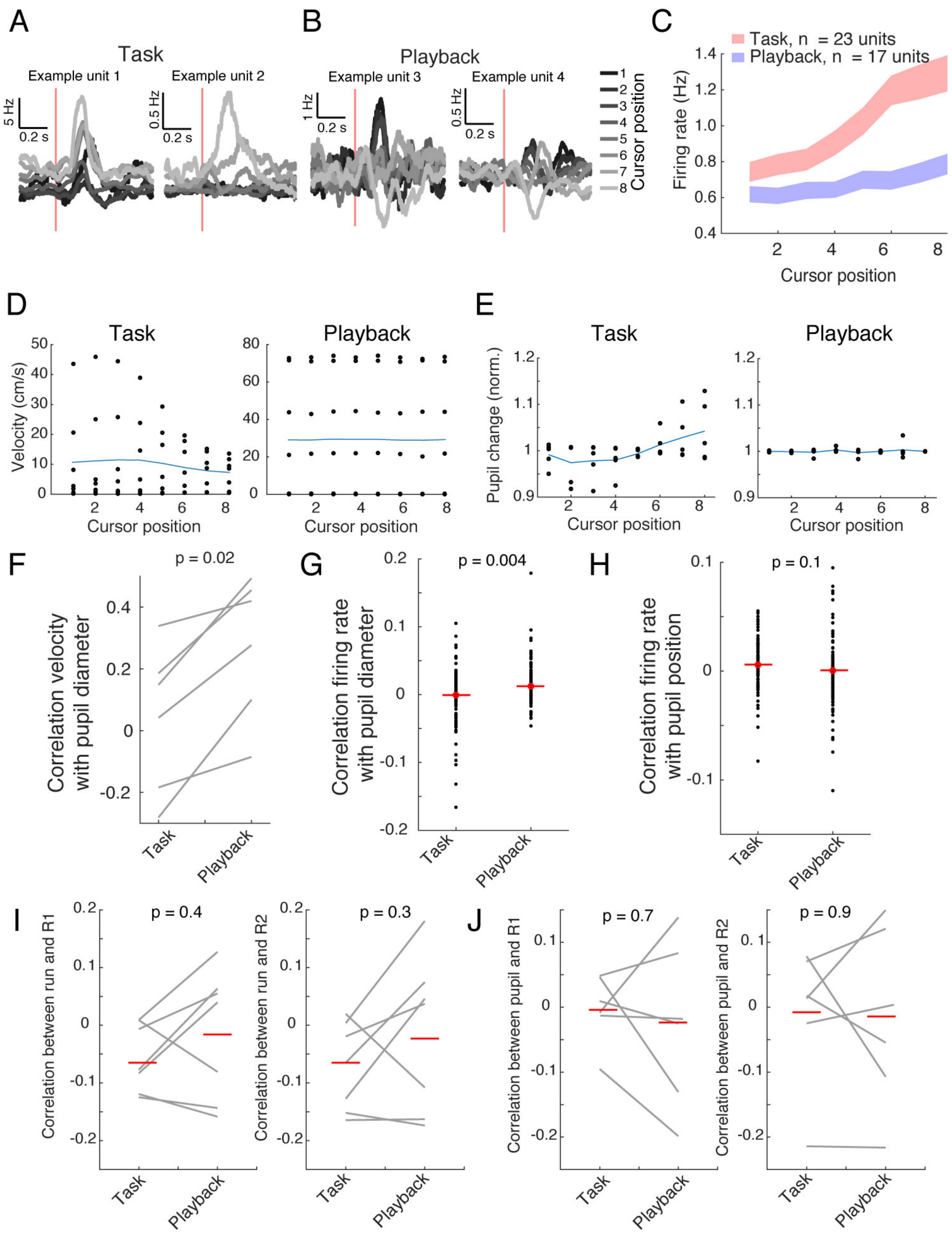

Supplemental Figure 5. Pupil and locomotion become uncoupled during task (related to Figure 5)

A. Responses to the visual cursor at the 8 monitor positions for two single units recorded during task performance. The red line denotes the time the cursor appeared; the trace colour denotes cursor position. Here the spiking responses have been smoothed by convolving with a gaussian kernel with standard deviation of 25 ms. **B.** Responses to the visual cursor at the 8 monitor positions for two single units recorded during passive

playback. The red line denotes the time the cursor appeared; the trace colour denotes cursor position. Here the spiking responses have been smoothed by convolving with a gaussian kernel with standard deviation of 25 ms. **C.** Firing rates for units recorded in 2 animals where the target cursor position was rewarded during passive playback (blue trace). The enhanced firing for the target-adjacent cursor positions during task performance (red trace) remained, suggesting the boosting was not simply reward expectation (95% confidence interval indicated, n = 2 mice, 23 units in the task condition and 17 units in the passive playback condition). **D.** Average running velocity at each cursor location during recordings during task (left) and playback (right), (n = 7 mice, final day of recording). **E**. Normalized pupil diameter at each cursor location during task (left) and playback (right), (n = 6 mice, final day of recording). **F.** Pupil diameter and running speed were significantly decorrelated during task performance compared to playback (reward collection periods and inter-trial intervals excluded, n = 6 mice, final day of recording, each line represents correlations for one mouse.) **G.** The correlation between single unit firing rate and pupil diameter was not different from zero during task, but slightly higher than zero during playback (reward collection periods and inter-trial intervals excluded, n = 6 mice, N = 105 units task, n= 122 units playback). **H.** The correlation between single unit firing rate and pupil position was not different from zero during task or playback conditions (reward collection periods and inter-trial intervals excluded, n = 6 mice, N = 105 units task, n= 122 units playback). **I.** R1 (left panel) and R2 (right panel) activity were not significantly correlated with running velocity, either during task performance or passive playback (reward collection periods and inter-trial intervals excluded, N = 7 mice, each line represents correlations for one mouse). The correlation between R1 or R2 and run velocity during task and passive playback were not significantly different from zero (t-test: for R1, p = 0.08, p=0.6, respectively for task and playback; for R2: p = 0.08, 0.7 respectively for task and playback), nor significantly different from each other (paired t-test, p = 0.4). **J.** R1 (left panel) and R2 (right panel) activity were not significantly correlated with pupil diameter, either during task performance or passive playback (reward collection periods and inter-trial intervals excluded, N = 6 mice, t-test; each line represents correlations for one mouse). The correlation between R1 or R2 and pupil diameter during task and passive playback were not significantly different than zero (t-test, for R1: p = 0.9, 0.7 for task, playback, respectively; for R2: p = 0.9, 0.8 for task, and playback, respectively), nor significantly different from each other (paired t-test, p = 0.4).

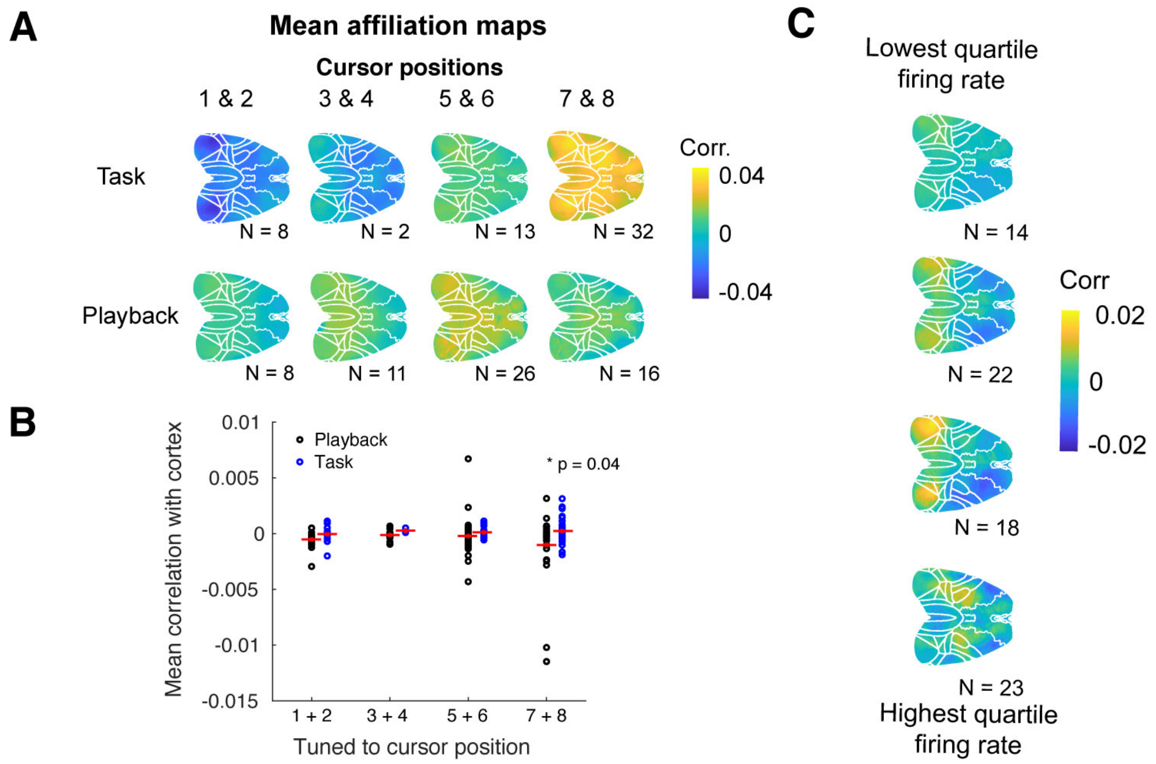

Supplemental Figure 6. Cortex-wide affiliations of units tuned to different cursor positions (related to Figures 5 & 6)
A. Spike trains for each unit were correlated with the activity of each pixel to build cortex-wide affiliation maps. These were sorted into bins based on which cursor position units were most responsive to. The average of these maps is shown in the top row for task performance, and the bottom row for passive playback. The reported N under each average map indicates the number of single units in that bin for that condition. Only units which had statistically significant tuning to a cursor location, and units with correlation maps that had >50% pixels with significant correlations (p values were Benjamini–Hochberg corrected for multiple comparisons, with a significance threshold set at a 5% false positive rate) were included. **B.** Mean correlation of spiking activity with calcium activity across dorsal cortex, sorted by units' cursor preference for task and playback. **C.** The trend evident in **A** is not due to higher firing rates of target cursor-tuned units, when units were organized into quartiles of mean firing rates rather than cursor preference. The reported N under each average map indicates the number of single units in that bin for that condition.

	[M/L, A/P] (mm)	M1	M2	M3	M4	M5	M6	M7
**Day 1**	**R1**	-1.5, 1.5	-2.5, 2	-2, 1.5	-2.5, 2	-2.5, 2.5	-2, 1.5	-1.5, 1
	**R2**	-2.5, 1.5	-2.6, 1	-2.2, 1	-2.5, 0.5	-2.5, 2	-2.5, 2.5	-2.5, 2
**Day 2**	**R1**	-1.5, 1.5	-2.5, 2	-2, 1.5	-2.5, 2	-2.5, 2.5	-2, 1.5	-1.5, 1
	**R2**	-2.5, 1.5	-2.6, 1	-2.2, 1	-2.5, 0.5	-2.5, 2	-2.5, 2.5	-2.5, 2
**Day 3**	**R1**	-1.5, 1.5	-2.5, 2	-2, 1.5	-2.5, 2	-2.5, 2.5	-2, 1.5	-1.5, 1
	**R2**	-2.5, 1.5	-2.6, 1	-2.2, 1	-2.5, 0.5	-2.5, 2	-2.5, 2.5	-2.5, 2
**Day 4**	**R1**	-1.5, 1.5	-2.5, 2	-2, 1.5	-1, 3	-2.5, 2.5	-2, 1.5	-1.5, 1
	**R2**	-2.5, 1.5	-2.6, 1	-2.2, 1	-2, 1.5	-2.5, 2	-2.5, 2.5	-2.5, 2
**Day 5**	**R1**	-1.5, 1.5	-2.5, 2.3	-2, 1.5	-2.5, 2	-2.5, 2.5	-1, 1	-2, 1.5
	**R2**	-2, 1.5	-2.5, 1.5	-2.2, 1	-2, 1.5	-2.5, 2	-2, 1.3	-1.5, 1.5
**Day 6****	**R1**	-1.5, 1.5	-2.5, 2.3	-2.5, 2.5	-2.5, 2	-2.5, 2.5	-1.5, 1	-2, 1.5
	**R2**	-2, 1.5	-2.5, 1.5	-2, 2	-2, 1.5	-2.5, 2	-2, 1.5	-1.5, 1.5
**Day 7**	**R1**	-1, 1.5	-2.5, 2.5	-2.5, 2.5	-2.5, 2	-2, 2.5	-2.5, 1.5	-2, 1.5
	**R2**	-1.2, 1.8	-0.5, 3	-2, 2	-1.5, 2.5	-1, 0.5	-2.5, 2	-1.5, 1
**Day 8**	**R1**	-0.8, 2.2	-1.7, 1.5	-1, 1.5	-2.5, 2	-2, 2.5	-2.5, 1.5	-2, 2
	**R2**	-1, 2	-1, 2	-1.5, 2	-1.5, 1.5	-1, 0.5	-2.5, 2	-2.5, 2
**Day 9**	**R1**	-1.2, 0.5	-2, 1.5	-1.5, 3	-2.5, 2	-2, 2.5	-2.5, 1.5	-1.5, 1.5
	**R2**	-2.2, 1	-1.5, 1	-1.5, 1.5	-1.5, 0.5	-1, 0.5	-2.5, 2	-2.5, 2
**Day 10**	**R1**	-1.5, 1	-2, 1.5	-2.5, 2.2	-2.5, 2	-2, 1.5	-2.5, 1.5	-1.5, 1.5
	**R2**	-1.2, 2	-1.5, 1	-1.5, 1.5	-1.5, 0.5	-1.5, 1.5	-2.5, 2	-1.7, 1.7
**Day 11**	**R1**	-1.5, 1	-2, 1.5	-2.5, 2	-2.5, 2	-2, 1	-2.5, 1.5	-1.5, 1.5
	**R2**	-1, +2	-1.5, 1	-1.5, 2	-1.5, 0.5	2, 1	-2.5, 2	-1.7, 1.7
**Day 12**	**R1**	-1.5, 1	-2.5, 2	-0.7, 0.5	-2.5, 2	-2, 1	-2, 1.5	-0.5, 0.5
	**R2**	-1, 1.5	-1.5, 1	-2, 2	-1.5, 0.5	2, 1	-2, 2.5	-1.5, 1.5
**Day 13**	**R1**	-1.5, 1	-1.2, 0.5	-1, 0.5	-1.5, 0.5	-2, 2	-1, 0.5	-0.5, 0.5
	**R2**	-1, 1.5	-1.5, 2	-2, 2	-2.5, 2	-1, 0.5	-2, 1	-1.5, 1.5
**Day 14**	**R1**	-1.5, 0.2	-1.2, 0.5	-1, 0.5	-1.5, 0.5	-1.5, 1.5		
	**R2**	-2, 2	-1.5, 2	-2, 2	-2.5, 2	-2, 2		
**Day 15**	**R1**	-1.5, 0.2	-1, 0.2	-1, 0.5	-1.5, 0.5	-1.5, 1.5		
	**R2**	-2, 2	-1.5, 2	-2, 2	-2.5, 2	-2, 2		
**Day 16**	**R1**		-1, 0.2	-1, 0.5	-1.5, 0.5	-1.5, 1.5		
	**R2**		-1.5, 2	-2, 2	-2.5, 2	-2, 2		
**Day 17**	**R1**					-1.5, 1.5		
	**R2**					-2, 2		

Supplemental Table 1. Control region coordinates (related to Figure 1)

Related to Figure 1B: approximate stereotaxic coordinates, in millimeters, of control regions on all days of training, reported in [medial/lateral, anterior posterior] pairs, with bregma = [0, 0], and coordinates to the left of, or posterior to, bregma reported as negative. Day 6, indicated with **, was the day that control regions were switched midway through the training session. The margin of error for each position is ~0.1mm.
